# iSeq: A New Double-Barcode Method for Detecting Dynamic Genetic Interactions in Yeast

**DOI:** 10.1534/g3.116.034207

**Published:** 2016-11-07

**Authors:** Mia Jaffe, Gavin Sherlock, Sasha F. Levy

**Affiliations:** * Department of Genetics, Stanford School of Medicine, California 94305; † The Laufer Center for Physical and Quantitative Biology, Stony Brook University, New York 11794; ‡ Department of Biochemistry & Cell Biology, Stony Brook University, New York 11794

**Keywords:** Saccharomyces cerevisiae, DNA barcoding, genetic interactions, systems biology, whole genome sequencing

## Abstract

Systematic screens for genetic interactions are a cornerstone of both network and systems biology. However, most screens have been limited to characterizing interaction networks in a single environment. Moving beyond this static view of the cell requires a major technological advance to increase the throughput and ease of replication in these assays. Here, we introduce iSeq—a platform to build large double barcode libraries and rapidly assay genetic interactions across environments. We use iSeq in yeast to measure fitness in three conditions of nearly 400 clonal strains, representing 45 possible single or double gene deletions, including multiple replicate strains per genotype. We show that iSeq fitness and interaction scores are highly reproducible for the same clonal strain across replicate cultures. However, consistent with previous work, we find that replicates with the same putative genotype have highly variable genetic interaction scores. By whole-genome sequencing 102 of our strains, we find that segregating variation and *de novo* mutations, including aneuploidy, occur frequently during strain construction, and can have large effects on genetic interaction scores. Additionally, we uncover several new environment-dependent genetic interactions, suggesting that barcode-based genetic interaction assays have the potential to significantly expand our knowledge of genetic interaction networks.

Genetic interaction (GI) studies are vital for our understanding of cellular wiring, and have contributed insights into biological function, genetic robustness, and network topology, and ultimately may help explain the heritability of complex traits and define new drug targets (Tong *et al.* 2001; Ooi *et al.* 2003; Wong *et al.* 2004; Ye *et al.* 2005; Kelley and Ideker 2005; Davierwala *et al.* 2005; Albert 2005; Schuldiner *et al.* 2005; Pan *et al.* 2006; Lehner *et al.* 2006; St Onge *et al.* 2007; Collins *et al.* 2007; Musso *et al.* 2008; Roguev *et al.* 2008; Costanzo *et al.* 2010, 2016; Zuk *et al.* 2012; Dutkowski *et al.* 2013; Martin *et al.* 2015). A GI between two mutations is defined as a deviation in the double mutant fitness from the multiplicative fitness of the two corresponding single mutants (Phillips *et al.* 2000; Segrè *et al.* 2005; St Onge *et al.* 2007; Costanzo *et al.* 2010). The current systematic and genome-wide GI studies performed in the eukaryotic model *Saccharomyces cerevisiae* are rooted in early synthetic lethal screens (Bender and Pringle 1991). These systematic studies—*e.g.*, SGA (synthetic genetic array; Tong *et al.* 2001), dSLAM (heterozygote diploid-based synthetic lethality analyzed by microarray; Pan *et al.* 2004), E-MAPs (epistatic miniarrays; Collins *et al.* 2006; Schuldiner *et al.* 2006), and GIM (GI mapping; Decourty *et al.* 2008)—were enabled by the yeast deletion collection (Winzeler *et al.* 1999), and measure interactions between complete gene knockouts. Together, these techniques have cataloged > 200,000 GIs between the nearly 6000 genes in *S. cerevisiae*, with the majority being measured by SGA under a single standard growth condition (Stark *et al.* 2006).

Despite these advances, continued progress toward a complete and accurate genetic interactome faces several challenges. One is the relative paucity of GI data measured in nonstandard growth conditions. To date, only a handful of genetic interactions have been characterized across different environmental conditions (*i.e.*, differential interactions). Yet, these studies have yielded additional biological discoveries including new genes involved in DNA repair (Bandyopadhyay *et al.* 2010; Guénolé *et al.* 2013) and cell wall integrity (Martin *et al.* 2015). Some evidence suggests that environment-dependent GIs constitute a sizable fraction of all GIs. For example, a study across >1000 environments found that ∼2/3 of single mutant fitness defects are not observed on the standard yeast medium, YPD (Hillenmeyer *et al.* 2008). Furthermore, systems-level flux balance analysis predicts a similar fraction of GIs would be undetectable in YPD (Harrison *et al.* 2007). Thus, environment-dependent GIs likely remain largely undiscovered. Also unknown is how GIs change across environments, a property that could be fundamental for descriptions of cellular physiology, or for assigning biological function to uncharacterized proteins.

A second challenge is the relative difficulty of performing experimental replicates using current GI screening technologies. Because double deletion strains must be rearrayed, or replica plated, for each experimental replicate, screens typically generate only two to four measurements per genotype. Correlation values reported between replicate screens range from 0.2 to 0.8 (Schuldiner *et al.* 2005; Baryshnikova *et al.* 2010; Costanzo *et al.* 2010; Dodgson *et al.* 2016). Screens with higher correlations (*e.g.*, ∼0.8 in Baryshnikova *et al.* 2010) generally measure the same double deletion segregant pool in duplicate, whereas lower correlations are observed in screens that compare double deletion segregant pools that have been constructed independently (*e.g.*, comparisons between double deletions in which the selectable markers at the two deletion loci are swapped). It has been suggested that there is segregating genetic variation underlying these observations (Jasnos and Korona 2007), but data in support of this claim have been lacking. Furthermore, comparison of results between different studies can be confounded due to factors such as the low reproducibility of single deletion fitness estimates (*r*^2^ = 0.14–0.7 when comparing seven studies, Baryshnikova *et al.* 2010), the definition of a GI itself (Mani *et al.* 2008), and the fitness differences that are observed on solid media *vs.* those measured in liquid (Musso *et al.* 2008).

The challenges above highlight two major limitations of current GI technologies. First, each strain that tests an individual GI must remain physically isolated from other strains, making it impractical to construct and store large GI libraries that contain many replicates per double mutant for retesting across multiple growth environments. Second, double deletions are typically constructed by sporulating small pools of diploids, and selecting for all cells that pass selection, resulting in an unknown number individual haploid segregants in each double deletion strain. Thus, it remains unknown how much genetic variation exists between clonal segregants with the same presumptive genotype derived from either the same or different crosses, some of which could be contributing to variability in fitness measurements and interaction scores.

Technologies that rely on recognizing or sequencing DNA barcodes might be more suited to testing GIs at high replication and across different experimental conditions, because individual strains can be stored and tested as pools (Pan *et al.* 2004; Decourty *et al.* 2008; Smith *et al.* 2009; Gresham *et al.* 2011; Robinson *et al.* 2014). Indeed, we have recently shown that half a million barcodes can be tracked simultaneously by barcode sequencing (Levy *et al.* 2015). Each strain in the yeast gene deletion collection contains a pair of unique barcodes at the deletion locus (Winzeler *et al.* 1999; Giaever *et al.* 2002), which can be used to measure relative fitness across conditions by monitoring the relative frequency of each barcode in a mixed pool by microarrays or amplicon sequencing (Pan *et al.* 2004; Decourty *et al.* 2008; Smith *et al.* 2009; Gresham *et al.* 2011; Robinson *et al.* 2014). In some applications of this approach, a second deletion is introduced into the barcoded deletion library either by mating with, or transformation of, a common deletion construct, with all resulting pairwise combinations assayed in a single pool (Pan *et al.* 2004; Decourty *et al.* 2008). In theory, barcoded double deletion pools such as these could be used to examine genetic heterogeneity within a double deletion by subjecting them to severe bottlenecks such that each barcode in a post-bottleneck pool derives from a single segregant. Repeated bottlenecks would likely select different segregant representatives for each barcode, allowing one to assay the impact of genetic heterogeneity on each GI. Once a pool is constructed, it can be screened repeatedly in multiple environments to quickly and cheaply detect dynamic GIs (Darby *et al.* 2012; Huang *et al.* 2012). However, it is difficult to apply these methods more widely, assaying deletion combinations beyond a handful of query genes against a large array of target genes.

One potential solution would be to barcode both the query and the target strains and use *double barcodes* to uniquely identify each double deletion combination. The major technical challenge to this approach lies in identifying which two barcodes reside within the same cell. If barcodes are located in different locations in the genome, then performing a pooled DNA preparation, or a pooled PCR of barcode libraries will destroy the association of the two barcodes that exist within the same cell. One possible solution is to use a PCR protocol that stitches the two barcode PCR products into a single amplicon before sequencing (Yu *et al.* 2011). However, individual genotypes must be PCRd independently in multiwell plates prior to sequencing. Emulsion PCR (Margulies *et al.* 2005; Williams *et al.* 2006), whereby barcode amplification of DNA from individual cells can occur in small water droplets in oil, could be combined with stitched PCR to reduce costs and improve throughput: both plastic consumables and PCR reagents would be minimized. However, it is currently unclear if emulsion PCR can be effective for large numbers of droplets within which PCR products can be stitched together for subsequent sequencing.

Here, we introduce a novel **i**nteraction **Seq**uencing platform (iSeq), and apply it to measuring GIs. The key innovation of iSeq is a system that recombines two barcodes that exist on homologous chromosomes such that they are brought into close proximity on the *same* physical chromosome *in vivo* to form a double barcode (Figure 1A). iSeq accurately assays the fitness of each uniquely marked strain in the pool by monitoring double-barcode frequencies over several growth bottleneck cycles using a quantitative double-barcode amplicon sequencing and counting protocol. In this pilot study, we demonstrate the utility of iSeq, by using it to measure the GIs between all pairwise combinations of nine deletions across three environments at high replication. For any given clonally derived double-barcode strain, we show that fitness measurements and iSeq interaction scores are highly reproducible across biological replicates, and find several new environment-dependent GIs. However, we find low reproducibility between different double-barcode strains ostensibly carrying the same double deletions, which cannot be explained by measurement error. By whole-genome sequencing 102 of our experimental strains, we find that segregating variation and *de novo* mutations that occur during strain construction can have large effects on GI scores, suggesting that validation of GIs may require multiple replicate measurements.

**Figure 1:**
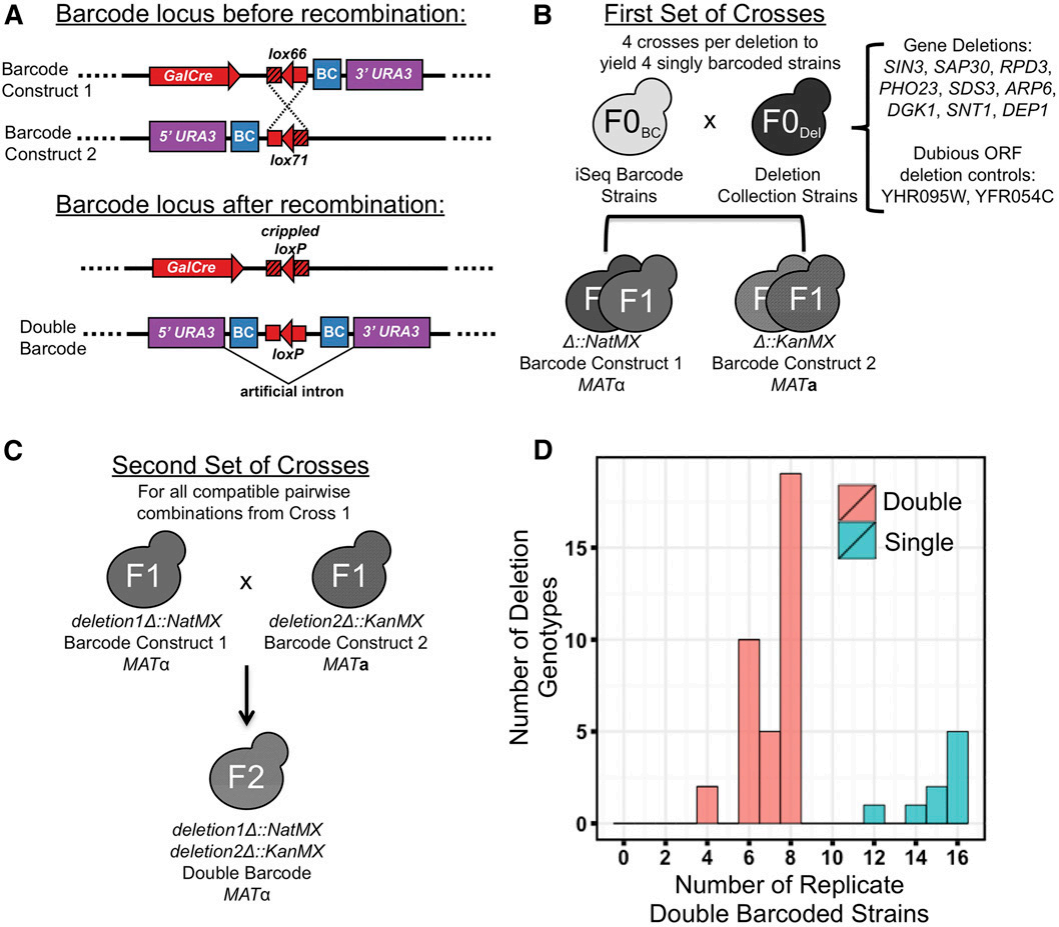
The iSeq platform. (A) Schematic of the iSeq barcode locus before and after Cre-mediated recombination. Two complementary barcode constructs are introduced to the same cell on homologous chromosomes via mating. Galactose-induced Cre recombination results in the two barcodes being on the same physical chromosome. Recombination events are selected for via a split *URA3* marker that is only functional after recombination. (B) First set of crosses to generate F1 strains. Two versions of each of the listed systematic deletion strains (*NatMX* and *KanMX*) are each mated to two strains with unique iSeq-compatible barcode constructs. The magic marker system is used to select for haploids of a specific mating type that contain a gene deletion and an iSeq barcode. (C) Second set of crosses to generate F2 experimental strains. All pairwise combinations of barcoded deletion strains are next mated together, recombination at the barcode locus is induced, and double-barcode double-deletion haploids are selected following sporulation. (D) Histograms of experimental replication. For our pilot of nine genes, 12–16 uniquely double barcoded strains were constructed for each of the nine possible single gene deletions (turquoise), and four to eight strains were constructed for each of the 36 possible double gene deletions (pink).

## Materials and Methods

Extended *Materials and Methods* and a detailed protocol for iSeq can be found in Supplemental Material, File S1.

### Yeast barcode library construction

Two complementary barcode libraries, consisting of 288 clones each, were generated in a MATalpha starting strain derived from BY4742 (*MAT*α *ura3*Δ0 *leu2*Δ0 *his3*Δ1 *lys2*Δ0) (Brachmann *et al.* 1998). This starting strain also carries the magic marker construct (Tong *et al.* 2004), which allows for selection of either *MATa* or *MAT*α haploids via growth on synthetic complete (SC) medium containing canavanine and lacking either histidine or leucine, respectively. The barcode construct in each strain of each library sits at the dubious open reading frame (ORF) YBR209W, and consists of a DNA barcode with 20 random nucleotides, a *HygMX* selectable marker, and either the 5′ half of the *URA3* selectable marker and lox71 in the 5′ library, or the 3′ half of the *URA3* selectable marker and lox66 in the 3′ library.

### Double-barcoded double-deletion yeast strain generation

Haploid gene deletion strains, carrying either *KanMX* or *NatMX* marked deletions, were derived from the diploid heterozygous deletion collection (Tong *et al.* 2001; Pan *et al.* 2004) for the following genes and dubious ORFs: *ARP6*, *SAP30*, *SDS3*, *PHO23*, *SIN3*, *DGK1*, *SNT1*, *DEP1*, *RPD3*, YHR095W, and YFR054C. Each of the 11 deletion strains marked with *KanMX* was mated to two unique strains from the 5′-barcode-construct-carrying yeast library. *NatMX* marked deletion strains were each mated to two strains from the 3′-barcode-construct-carrying yeast library. Resulting diploid strains from each cross, and carrying a deletion and the barcode construct, were sporulated and plated for haploid single colonies.

To obtain strains carrying two gene deletions and both complementary barcode constructs, all pairwise combinations of singly barcoded deletion strain were mated. In each resulting diploid, Cre-mediated recombination was induced at the barcode locus by growing on SC + 2% Galactose –Ura at 30° for 2 d. Cells were sporulated, and unsporulated diploids were digested using zymolyase as described (Herman and Rine 1997) before selecting single haploid colonies.

### Pooled growth

The 393 barcoded single and double gene deletion strains were frogged from frozen glycerol stocks to 1 ml liquid YPD in 2 ml 96-well plates, and placed at 30°. After 3 d of growth, all strains were pooled, glycerol was added to a final concentration of 17%, and aliquots were stored at −80° for future inoculations. The eight barcoded wild type (WT) control strains, generated from the matings of two dubious ORF barcoded deletion strains, were grown overnight in liquid YPD, pooled, glycerol added, and aliquots were stored at −80° for future inoculations.

The pooled fitness assay was carried out in three growth conditions: YPD, YPD 37° and YPEG (YP + 2% EtOH, 2% glycerol). The alternate conditions were chosen because in the *Saccharomyces* Genome Database, seven of nine of the single gene deletions are annotated as heat sensitive, and four of nine have decreased respiratory growth.

For pooled growth fitness estimates, the double-barcoded WT and double-barcoded mutant pools were mixed at a 50:50 cellular ratio. For YPD, YPD 37°, and YPEG cultures, 1.5625 × 10^9^, 6.25 × 10^8^, 6.78 × 10^9^ cells of this mixture, respectively, were used to inoculate 100 ml liquid of medium in a 500 ml flask, in triplicate. The cells were cultured shaking at 230 rpm at 30° or 37°. Every 24 hr, for a total of eight time points, 12.5 ml culture were transferred to 87.5 ml fresh medium, and placed back in the incubator. At each transfer, the remaining overnight cultures were split into two 50 ml tubes, spun down and resuspended in a 5 ml solution of 0.9 M sorbitol, 0.1 M EDTA, 0.1 M Tris-HCl, pH 7.5 for DNA extractions.

### Barcode sequencing

Barcode sequencing was performed as previously described (Levy *et al.* 2015). Briefly, genomic DNA was extracted by spooling. A two-step PCR was carried out on 14.4 μg genomic DNA to amplify the barcoded region, add multiplexing tags, and add Illumina paired-end sequencing adaptors. Four initial time points were pooled and sequenced on the Illumina MiSeq. Remaining libraries were pooled and paired-end sequencing was performed over four lanes on the Illumina HiSeq 2000 (10, 11, 20, and 23 libraries per lane). Additionally, 21 libraries were resequenced on one lane on Illumina HiSeq 2000 to test for sequencing noise.

Custom Python scripts were used to demultiplex the time points from the Illumina data, and to determine the number of reads matching each known double barcode in the pool at each time point. The observed number of counts for each strain, at each time point, in each experiment, is available in Table S5.

### Fitness and GI estimates

To estimate the fitness of each strain in the pool, barcode counts at each of the first four time points were normalized for each strain by first dividing by the total number of counts at that time point to get a relative frequency. These frequencies were then normalized to the change in WT frequency, and then subsequently divided by the relative frequency at the first time point. After taking the natural logarithm of each of these normalized frequencies, a least squares linear regression was fit using the lm function in R, using a predefined intercept of 0. The fitness estimate for each strain was then defined as 1 + *m*, where *m* is the slope of the fitted line.

To estimate quantitative genetic interaction scores, we calculated the deviation, ε, of the observed fitness of each double mutant strain (*f_ij_*) in the pool from the expected fitness, based on the product of the observed fitness of the single mutant strains, *f_i_* and *f_j_*, as:

$$\text{ε}=f_{\mathrm{ij}}-\left(f_{i}\times f_{j}\right)$$

Fitness and interaction score estimates for each experimental strain across each replicate can be found in Table S2. To call interaction scores as significantly positive or negative, a 95% confidence interval (C.I.) was calculated around the mean score from the four to eight strains with identical pairs of gene deletions. CIs for each gene pair across each condition can be found in Table S4.

### Optical density fitness estimates

A total of 393 barcoded strains were streaked for single colonies on YPD. A single colony was used to inoculate a 2 ml overnight YPD culture. For three replicates of each strain, 2 μl of this overnight culture was used to inoculate 98 μl YPD in a 96-well plate. This plate was placed in the TECAN (GENios), and optical density (OD) at 595 nm (OD_595_) was taken every 15 min for 90 cycles, or 180 cycles for exceptionally slow growing strains.

To estimate fitness of each strain, the region of the curve during exponential growth was found for each strain by fitting a linear regression to each window of 10 time points, across all 90 total time points (90 total windows). This windowing method was employed to adjust for the fact that not all strains started at the same OD, and to avoid choosing arbitrary threshold values within which to calculate the doubling time. The fitted line corresponding to the window with the maximum slope, and therefore maximum growth rate, was used to calculate a doubling time for each strain. Fitness estimates were calculated by dividing the doubling time of a WT strain (generated above) that was included on the plate by the doubling time of the experimental strain (St Onge *et al.* 2007). Observed doubling times and fitness estimates for each strain are available in Table S2 and Table S5.

### Whole-genome sequencing

Strains were streaked for single colonies from frozen stocks, and grown up overnight in YPD at 30°. Genomic DNA was isolated with the YeaStar Genomic DNA Kit (Zymo Research). Libraries for Illumina sequencing were constructed in 96-well format as previously described (Kryazhimskiy *et al.* 2014), pooled and analyzed for quality using Bioanalyzer (Agilent Technologies) and Qubit (Life Technologies), and sequenced on one lane of Illumina HiSeq 2000. Reads were trimmed for adaptors, quality and minimum length with cutadapt 1.7.1 (Martin 2011). Reads were mapped to the reference genome with BWA version 0.7.10-r789 (Li and Durbin 2009). And variants were called with GATK’s Unified Genotyper v.3.3.0 (McKenna *et al.* 2010). Significant changes in copy number were discovered using the CNV-Seq package (Xie and Tammi 2009). SIFT was used to predict the protein function tolerance of amino acid changes resulting from SNPs verified by visual inspection using samtools tview and mpileup (Kumar *et al.* 2009; Li *et al.* 2009).

### Data availability

Genotypes of all strains, as well as all primer and plasmid sequences are included in File S1. All strains and plasmids are available upon request. Barcode sequencing counts observed for each strain at each time point in each experiment can be found in Table S5 and were extracted from raw fastq sequencing files using custom Python scripts. Fitness and interaction score estimates for each strain in each condition can be found in Table S2. Variants called for each of 102 whole-genome sequenced strains are listed in Table S3. Whole-genome sequencing data have been made publicly available under the NCBI BioProject accession number PRJNA344503 and the SRA accession number (SRP090639).

## Results

### The iSeq platform

The iSeq platform consists of a novel double-barcoding technology combined with a pooled fitness assay. The double-barcoding technology uniquely identifies both parents of a mating event. While iSeq could be used to study interactions between any two genomes or genetic elements, here, we use iSeq in combination with gene deletion strains to assay interactions between pairwise combinations of deletions over three environments. Our system functions by first introducing loxP recombination sites at a common chromosomal location in both *MATa* and *MAT*α haploids. Barcodes are placed on opposite sides of the loxP sites such that mating and Cre induction causes recombination between homologous chromosomes, resulting in a barcode-loxP-barcode configuration on one chromosome (Figure 1A). Because these double barcodes are unlikely to dissociate during genomic DNA preparation, and are in close enough proximity to be sequenced by short-read single-end or paired-end sequencing, pools of double-barcode strains can subsequently be assayed using standard pooled barcode sequencing approaches (Pan *et al.* 2004; Decourty *et al.* 2008; Smith *et al.* 2009; Gresham *et al.* 2011; Robinson *et al.* 2014).

### Experimental design: genes and controls chosen for iSeq validation

To validate our approach, we chose a modestly sized group of nine genes, and used iSeq to measure the genetic interactions between the 36 possible gene pair combinations. To assess iSeq across a range of values, the genotypes in this set were chosen to include a range of published quantitative interaction scores (Table S1). Furthermore, seven of the gene pairs have no published interaction, providing negative controls as well as the possibility of detecting novel environment-dependent genetic interactions upon growth in new conditions. By “marking” each of these gene deletions with four different iSeq barcodes as outlined in Figure 1B and below, we were able to generate up to eight independently constructed strains for each double mutant assayed, thus providing a high level of biological replication.

Single mutant controls, required for interaction score estimates, were generated via the same protocol as their double mutant counterparts, ensuring that all experimental strains carried iSeq double barcodes and the same set of markers. When generating single mutants, we used dubious ORF deletions as placeholders for the second gene deletion. The two dubious ORFs we chose, YHR095W and YFR054C, are not expressed, have no fitness defect when deleted under the conditions in which they have been tested, and have no reported genetic interactions in the BioGRID database (Stark *et al.* 2006; Breslow *et al.* 2008; Li *et al.* 2008; Pelechano *et al.* 2013). Thus, strains carrying one gene deletion and one dubious ORF gene deletion should be reasonable proxies for single mutants. In total, we assayed multiple replicates of 36 double, and nine single gene deletions.

### Construction of iSeq deletion strains

To generate deletion strains carrying the double-barcoding system, we first constructed two yeast iSeq barcode libraries (288 strains each, in the same *MAT*α starting strain) by replacing the dubious ORF YBR209W with one of two complementary plasmid-derived constructs via homologous recombination. The YBR209W site has been used successfully in our laboratory as an integration site for heterologous genetic elements (Kao and Sherlock 2008; Levy *et al.* 2015); its transcript is not expressed, and its absence does not significantly affect fitness (Breslow *et al.* 2008; Li *et al.* 2008; Pelechano *et al.* 2013). A detailed protocol for making these yeast barcode libraries and subsequently generated double barcode strains can be found in File S1.

We next chose *MATa* strains derived from the systematic deletion collection (Winzeler *et al.* 1999) that carry either a *NatMX* or a *KanMX* selectable marker at the deletion locus (F0_Del_ haploids), and mated these to *MAT*α clones from each barcode library (F0_BC_ haploids). Resulting diploids were sporulated, and the magic marker system (Tong *et al.* 2004) was used to select *MATa* or *MAT*α haploid clones containing both the iSeq barcode and either a *KanMX* or *NatMX* marked deletion, respectively (F1 haploids, Figure 1B). After selection, and for each clone, the mating type was verified, and the iSeq barcode sequence identified. In total, we barcoded each of the nine gene deletions and two dubious ORF deletions with four different single iSeq barcodes, two barcodes for each version of the deletion (*KanMX* or *NatMX*) (Figure 1B).

To construct double-barcoded double-deletion strains, we mated all pairwise combinations of *KanMX* and *NatMX* strains, induced recombination at the iSeq barcode locus, sporulated, eliminated diploids by zymolyase digestion, and then selected haploid clones (F2 haploids, Figure 1C). After all matings, each double gene deletion is represented by up to eight unique iSeq double barcodes, and each single gene deletion, which brings together a gene deletion with a dubious ORF deletion, is represented by up to 16 double barcodes (Figure 1D). Finally, eight double-barcoded control strains, each intended to represent a wild-type phenotype, were generated by bringing together two dubious ORF deletions. In total, we generated 393 double-barcoded strains: 257 double gene deletions and 136 single gene deletions.

### Pooled fitness estimates of double-barcode double-deletion strains

**Figure 2:**
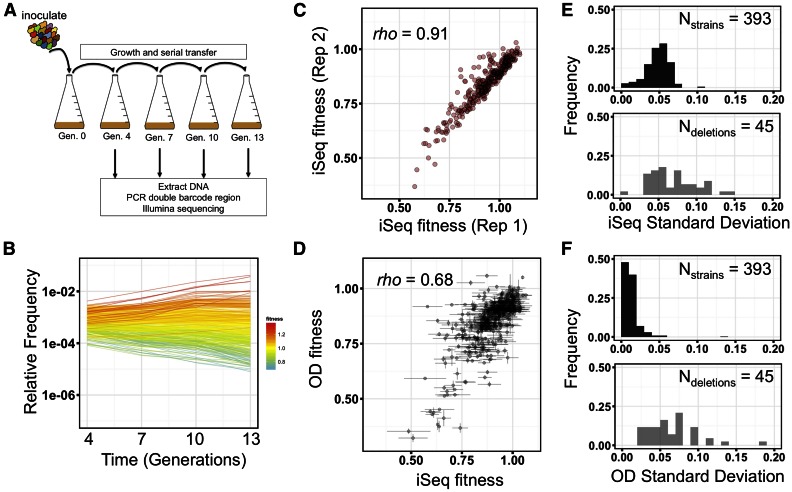
iSeq pooled fitness assay and reproducibility of measurements. (A) A schematic of the iSeq pooled fitness assay. Double-barcode pools are grown by serial transfer every ∼3 generations. At each transfer, relative double-barcode frequencies are assayed by short-read amplicon sequencing. (B) Representative plot of relative frequencies from a pooled fitness assay. Each line is an individual double-barcode strain. Colors indicate the fitness estimate of each strain. (C, D) Scatter plot of fitnesses between two biological replicates of the iSeq assay (C) or between iSeq and a multi-well OD-based measurement (D). Spearman’s *rho* is shown on each plot. (E, F) Frequency distributions of SD of the same double barcode across three growth replicates (black), or the same double deletion across four to eight double barcodes (gray) for iSeq (E) or OD-based (F) fitness measurements.

We pooled all 393 double-barcode haploid strains, and mixed this pool with a pool of the eight putative WT control strains at a ratio of 50:50. We combined pools in this way so that at least 50% of cells start with an approximately WT fitness, thereby minimizing the effects of strain–strain interactions between different mutant genotypes during pooled growth. We propagated this combined pool by serial batch culture in YPD at 30° at an effective population size of 8 × 10^9^, bottlenecking 1:8 at each transfer (Figure 2A, every three generations). This design, which samples at multiple and relatively frequent time points, was chosen for three reasons. First, multiple measurements increase the sensitivity to detect subtle fitness differences between strains. Second, measurements every few generations enable accurate estimates of low fitness genotypes that are rapidly driven to extinction. Third, this large population size was required for our DNA extraction and barcode sequencing protocol, such that sufficient material could be extracted for barcode sequencing (see *Materials and Methods*). At each bottleneck, we extracted genomic DNA, and then sequenced the double barcodes to estimate the relative frequency of each strain in the population (Figure 2, A and B and Figure S1A) (Smith *et al.* 2009; Levy *et al.* 2015). The slope of a log-linear regression of the change in frequency relative to WT over the four time points was used as the measure of fitness for each double-barcoded strain (see all estimates in Table S2). For each double-barcoded strain, fitness measurements were highly reproducible across biological replicates (Figure 2C and Figure S1B, Spearman’s *rho* = 0.91–0.97, *P* < 2.2 × 10^−16^). We next investigated the possibility that our pooled fitness assay might have larger errors on lower fitness genotypes, as those genotypes could be quickly driven to low frequencies where sampling errors have a larger effect. We find no significant association between fitness and standard deviation (SD) SD of fitness in our assay (Figure S2A, Spearman’s *rho* = −0.07, *P* = 0.19), with the least-fit double barcode still having a low fitness error (*s* = 0.49 ± 0.11) in YPD. We do, however, find greater errors on a small subset of low fitness strains in the two other conditions tested (see below), as, in these conditions, these strains are typically driven below our detection limit after just two time points (Figure S2, B and C).

To validate the fitness obtained by iSeq, and to determine whether pooling strains had an effect on strain fitness, we next compared iSeq fitness measurements to those from a standard growth assay. We grew each strain in an individual well of a multi-well plate, collecting OD-based growth curves, and using the maximum exponential growth rate as a proxy for fitness (see *Materials and Methods*). Exponential growth rate might not be expected to correlate highly with fitness during sequential batch growth since potentially important growth dynamics of sequential batch growth, such as entering or leaving saturation, are not captured. Nevertheless, we find a significant positive correlation between the two methods, indicating that potential strain–strain interactions during pooled growth had little to no effect on our fitness estimates (Figure 2D, Spearman’s *rho* = 0.68, *P* < 2.2 × 10^−16^, *N* = 391 strains).

However, despite the reproducibility of our fitness estimates for any given double barcode across replicate cultures, and its concordance with a secondary measure of fitness, we found that there was variability in fitness between strains carrying different double barcodes but the same putative gene deletions. The median SD of fitness for the same double barcode measured across independent cultures is 0.049, while the median SD of fitness of strains with different barcodes but the same deletions is 0.063 (Figure 2E). This high variability across strains was also observed in our independent OD-based measure of fitness, indicating it was not an artifact of measuring the fitness in pooled format (Figure 2F).

### Influence of genetic background on fitness

Above, we observed that the fitness varied when comparing strains carrying identical gene deletions but unique double barcodes (Figure 2, E and F). We hypothesized that this variation between double barcodes may be caused either by segregating genetic variation in the parental strains, and/or by *de novo* mutations that occurred during the growth, mating, or sporulation steps of strain construction. To investigate this possibility, we performed whole genome sequencing on 10 F0_BC_, 6 F0_Del_, 24 F1, and 39 F2 strains that were related by descent (see Figure 1, B and C, and Table S3). We also sequenced our eight control F2 strains, which each carry two dubious ORF deletions, and their corresponding F0_Del_ and F1 parental strains, in order to help determine for any mutations that did arise, whether they arose due to the strain generation protocol, or due to the presence of a gene deletion that causes a severe fitness defect.

A subset of strains from the gene deletion collection has been shown to carry both aneuploidies and suppressor mutations (Hughes *et al.* 2000; Teng *et al.* 2013). Thus, as our sequenced F0_Del_ strains were derived from the deletion collection, we first looked for mutations present in these strains. In each of the F0_Del_ strains, we observed between one and four private SNPs that were not observed in any other strains except direct descendants, with similar numbers observed between the gene and control groups. Only one aneuploidy was observed, in the *PHO23* deletion strain, on chromosome XI. Similarly, in our sequenced iSeq barcode library strains (F0_BC_), eight of 10 carried either one or two private SNPs, and no aneuploidy was observed.

**Figure 3:**
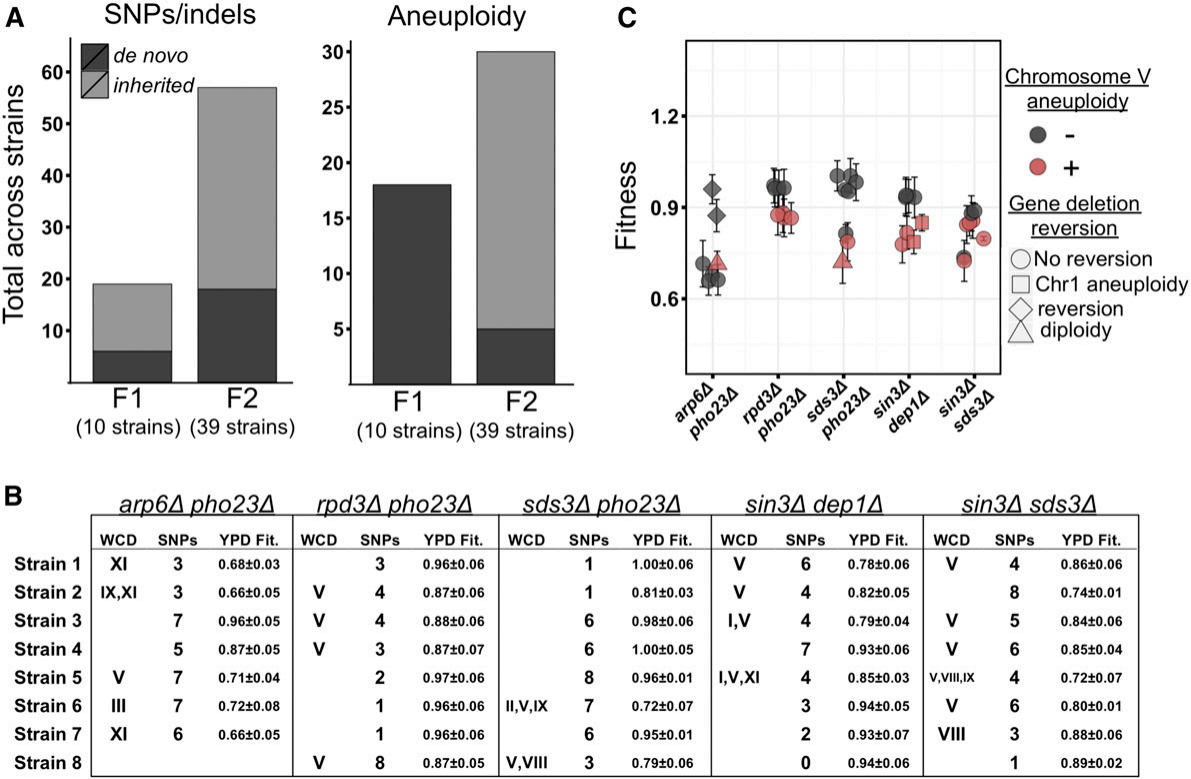
Segregating and *de novo* genetic variation revealed by whole-genome sequencing. (A) Total number of unique SNPs, or small indels (left), or aneuploidy events (right), observed across all F1 or F2 strains for which both parental strains had been sequenced (*N* = 10 and *N* = 39 strains, respectively). Bars are shaded by whether the observed variant was observed in a parental strain (light gray), or appeared to have occurred *de novo* during strain generation (dark gray). (B) For each of the strains sequenced (rows) in each of the double deletion groups, “WCD” indicates identities of duplicated chromosomes, “SNPs” indicates the total number of single nucleotide polymorphisms or small indels observed, and “YPD Fit.” indicates iSeq estimate in YPD. (C) Fitnesses for each whole-genome sequenced F2 strain. Color indicates chromosome V duplication events, and shape indicates gene reversion events in which sequencing reads mapped to one or two genic region(s) expected to be deleted. Error bars are the SD of estimates across three biological replicates.

We next studied the mutations present in the 24 F1 strains carrying one gene deletion and one iSeq barcode (see Figure 1B). Surprisingly, aneuploidy was extremely common, with 14 strains having an extra copy of at least one chromosome, and, of those, 12 strains carrying an extra copy of chromosome V. We also observed aneuploidy in three of the eight F1 control strains, indicating the aneuploidies were not a response to a specific gene deletion, but more likely a general result of the strain generation procedure. In addition to aneuploidy, one to seven SNPs were observed in 23 of the 24 F1 strains. Because we had sequenced both F0 parents for 10 of the F1 strains, we next determined how much of the observed variation had been inherited, and how much had occurred *de novo*. In total, 13 of the 19 unique SNPs observed in these 10 F1 strains were also observed in a parental strain, while none of the 18 aneuploidy events were present in a parental strain (Figure 3A).

Next we analyzed the genomes of the 39 F2 strains, which were generated after the second round of mating, and were used in the pooled fitness assay (see Figure 1C). First, as with the F1 strains, aneuploidy was common (Figure 3B). Of the 39 sequenced F2 strains, 21 had at least one chromosome duplicated (54%), and, of these, as with the F1 strains, chromosome V was the most likely to be duplicated (16 of 21 strains). The strains aneuploid for chromosome V generally had lower fitness than strains with the same gene deletions but no anueploidy (Figure 3C). A duplication of chromosome V was also observed in one of the eight F2 control strains, indicating these aneuploidies can occur in the absence of gene deletions. In total, 25 of 30 aneuploidies observed in the 21 F2 strains appeared to be inherited, as the aneuploidies were also observed in at least one related F1 parent (Figure 3A). Aneuploidies also appeared to be lost. Of the seven crosses where both F1 parents carried the same duplicated chromosome, in three cases F2 progeny did not have the aneuploidy.

Second, by examining the coverage in the genic regions that we expected to be deleted, we observed that six of the 39 sequenced F2 strains actually carried a copy of one or both of their two intended gene deletions. In two cases, aneuploidy of chromosome I yielded a heterozygous *DEP1* gene deletion. Two other cases (in putative *arp6*Δ*pho23*Δ and *sds3*Δ*pho23*Δ strains) contained reads mapping to the expected gene deletions, as well as several heterozygous SNPs, suggesting that they are diploids that somehow managed to survive digestion by zymolyase and haploid selection via the magic marker system. The two remaining cases contained reads mapping to the *PHO23* ORF, even though it was intended to be deleted, but no evidence of either aneuploidy or diploidy. We hypothesize that a rare recombination event reinstated the *PHO23* sequence after the second mating step to a strain carrying a wild-type *PHO23*. These reversions did not always lead to an increase in fitness as compared to other strains in the same group, as they often coincided with other events such as aneuploidy (Figure 3C). None of the F0_Del_ strains yielded sequencing reads at their gene deletions, while two F1 strains did, due to aneuploidy (in *DEP1* or *SDS3*), indicating these gene reinstatement events can occur after just one round of mating. No read coverage was ever observed in any of the 27 sequenced dubious ORF deletions (F0_Del_, F1, and F2), suggesting that these reversion events may be selected for because they result in increased fitness.

Finally, there were a total of 57 unique SNPs and small indels segregating across the 39 F2 double-deletion strains sequenced (Table S3). Of these 57 SNPs, 39 we present in an F1 parent (68%), while the rest had occurred *de novo* after the second round of mating (Figure 3A). Up to eight SNPs were observed in each double mutant strain, with a median of four SNPs per strain (Figure 3B). The number of SNPs per strain did not vary significantly across the five double deletion groups (*P* = 0.36, Kruskal-Wallis rank sum test). We also observed two to seven SNPs in our eight control F2 strains, illustrating that similar numbers of mutations accumulated in the absence of functional gene deletions. A majority (54%) of the SNPs and indels either fall in intergenic regions, result in synonymous changes, or result in amino acid changes predicted to be tolerated (Kumar *et al.* 2009). However, 19% resulted in frameshifts, premature stop codons, or nonsynonymous changes predicted to affect protein function (see *Materials and Methods*) (Kumar *et al.* 2009). There was no significant enrichment for any GO terms for the genes hit by SNPs and indels with functional consequences; however, this might be due to the small sample size. Regardless, segregating variation likely underlies some of the differences in fitness observed for different double-barcoded strains carrying the same gene deletions.

### Interaction score estimates using double barcodes

**Figure 4:**
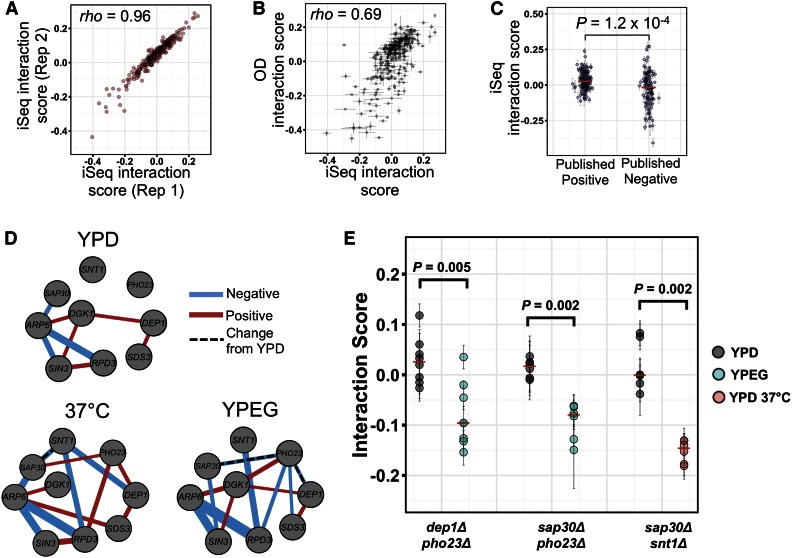
Identifying environment-dependent genetic interactions with iSeq. (A, B) Scatter plot of interaction scores between two biological replicates of the iSeq assay (A), or between iSeq and a multi-well OD-based measurement (B). (C) Interaction scores for individual strains carrying gene deletion pairs with a previously published positive (left) or negative (right) interaction. (D) The GI networks in each environment. For network edges, the color represents positive (red) or negative (blue) interaction scores, the width indicates relative magnitude of each score, and dashed lines are significant changes between YPD and another environment. (E) GI scores of all double-barcode replicates for three double deletions in two environments. Points and error bars in (B, C, E) are mean ± SD across three growth replicates. Red dashes in (C, E) are median values. *P*-values in (C, E) are Wilcoxon Mann-Whitney rank-sum test, and are 10% FDR corrected in (E).

Despite the genetic variation present in our strains, we were still able to calculate an interaction score for each strain using our fitness data. An interaction score, ε, is defined as the difference between the observed double mutant fitness, and its expected value based on the product of the fitnesses of the two corresponding single mutants (Phillips *et al.* 2000; Segrè *et al.* 2005; St Onge *et al.* 2007; Costanzo *et al.* 2010). Using this definition, we find that interaction scores for each double-barcode strain are highly reproducible between pooled growth replicates (Figure 4A and Figure S1C, Spearman’s *rho* = 0.96–0.98, *P* < 2.2 × 10^−16^, *N* = 255 strains), and correlate with interaction scores derived from the maximum exponential growth rate of single and double mutants (Figure 4B, Spearman’s *rho* = 0.69, *P* < 2.2 × 10^−16^, *N* = 255 strains). However, here too we find high variance between double barcodes that represent the same putative double knockout genotype. The median SD of interaction scores across strains with identical gene deletions is 0.072, 2.5-fold higher than the variance of each double barcode across pooled growth replicates (median SD = 0.072 *vs.* SD = 0.028, *P* = 2.2 × 10^−16^, Wilcoxon rank-sum test, *N* = 36 gene deletion pairs and 255 strains).

We next compared the interactions we identified with those collected through literature curation (Stark *et al.* 2006). We do note, however, that these published interactions are generally derived from colony growth on plates, and some interactions can be condition-specific, such that they are only observable either during growth in liquid, or when assayed on plates (Musso *et al.* 2008). Of the 36 gene pairs we tested, 14 have a reported negative genetic interaction, 15 a positive reported interaction, and seven have no reported interaction. Our scores for interactions in strains in the positive group were significantly different from those in the negative group (Figure 4C, *P* = 1.2 × 10^−4^, Wilcoxon rank-sum test), suggesting that, despite the scores being generated from different experimental conditions, and the known genetic variation our strains, there are similar observable trends.

To compare iSeq interaction scores to those previously reported from large-scale systematic screens, we calculated a mean interaction score for each double deletion (four to eight double barcodes per double gene deletion, with three replicate growth experiments each). Interaction scores derived from iSeq weakly correlate with those derived from two previous studies (Collins *et al.* 2007; Costanzo *et al.* 2010) (Figure S3, Collins: Spearman’s *rho* = 0.36, *P* = 0.063, *N* = 28 gene pairs; and Costanzo: Spearman’s *rho* *=* 0.38, *P* = 0.005, *N* = 33 gene pairs). As discussed above, complete agreement is not necessarily expected between different assays because they are performed in different growth conditions.

### Measurement of differential interactions using iSeq

We next performed two additional pooled fitness assays on our set of strains—one in heat stress (YPD 37°) and one in a nonfermentable carbon source (ethanol and glycerol, YPEG). As we observed in rich medium, fitness and interaction score estimates in the two new growth conditions were highly reproducible across replicate cultures (Figure S1, B and C, Spearman’s *rho* = 0.97–0.99, *P* < 2.2 × 10^−16^, fitness median SD = 0.027, interaction score median SD = 0.024), while there was only a weak negative correlation between fitness and the SD of fitness across replicate cultures (Figure S2, B and C).

To determine whether there are changes in interaction scores across conditions, we first called significant interactions in each of the three conditions using 95% CIs (see Table S4 and Figure 4D). Though many changes in sign and magnitude of interaction scores were observed between YPD and the two alternate conditions, a total of three gene pairs changed interaction score in a statistically significant manner [Figure 4E, *P* ≤ 0.005, *N* = 6–8 strains, Wilcoxon rank-sum test, 10% false discovery rate (FDR)]. Two gene deletion pairs (*dep1*Δ*pho23*Δ and *sap30*Δ*pho23*Δ) had no interaction in YPD but interact negatively in YPEG. One other gene deletion pair (*sap30*Δ*snt1*Δ) changed from no significant interaction in YPD at 30°, to a negative genetic interaction in YPD at 37°.

## Discussion

We have developed a new double barcode interaction sequencing technology (iSeq) that can be used to quantitatively examine pairwise GIs. iSeq’s double barcoding system allowed us to use pooled serial batch growth and high-throughput sequencing to measure the fitness of hundreds of double-deletion strains simultaneously—an approach previously only possible with pools of single deletion strains, or double deletions carrying a common deletion. Our method produces extremely reproducible fitness, and GI estimates for the same double barcode across replicate pooled growth experiments. Furthermore, the pooled iSeq fitness and GI scores correlate well with measurements made during individual growth, indicating that pooled growth does not confound our results. At current sequencing costs, considering an average coverage of 100 reads per strain for each of five time points (and 50% of the pool consisting of a barcoded control strain), we estimate a sequencing cost of $0.02 per GI per replicate per environment; these costs will fall at the same rate that sequencing does.

Importantly, we have illustrated iSeq’s ability to measure variance between individual, clonally derived strains with the same presumptive genotype by assaying several replicate strains in parallel. This ability could be applied to validate GIs measured in large screens with high replication. Here, by performing iSeq with four to eight independent constructs of the same double deletion, we found a high variance in both fitnesses and GI scores. The median correlation value for comparisons between our eight replicate strains per double gene deletion was 0.42, similar to previous reports of 0.2–0.5 (Schuldiner *et al.* 2005; Jasnos and Korona 2007; Dodgson *et al.* 2016). However, ours is the first study, to our knowledge, to use whole genome sequencing to investigate the underlying genetic variation that might confound GI measurements and lead to relatively low reproducibility. Our observation of new aneuploidies and SNPs after the first round of mating means mutations can accumulate quickly. Furthermore, these new mutations occurred prior to the Gal-induced Cre activity, and were also observed in dubious ORF deletion carrying controls, leading us to believe they were not an artifact specific to the deletion strains we chose, or the barcoding system itself.

However, several factors may limit generalizing our findings to previous GI studies. First, our study used the magic marker construct carrying the MFA/MFalpha promoters to select haploids. The MFA/MFalpha construct is known to be more leaky and prone to diploidization than the newer construct that uses *STE2/STE3* promoters, which is now more commonly used (Tong and Boone 2007; B. Andrews, personal communication). Further experimental work would be required to directly compare rates of aneuploidy accumulation with the *STE2/STE3* construct. It is also possible that the deletions we chose to examine have higher than average rates of mutation or chromosome segregation defects. Indeed, four of the double deletions we sequenced contain at least one gene shown to be involved in chromosome maintenance (*SIN3*, *SDS3*, and *RPD3*) (Wahba *et al.* 2011). Additionally, we chose a set of deletions with generally severe fitness effects, which might be more likely to accumulate additional fitness-altering mutations. Consequently, we did observe a slightly elevated accumulation of aneuploidies and SNPs in our strains carrying gene deletions compared to those carrying dubious ORF deletions (Table S3). Finally, our strains went through one additional round of mating and selection compared with standard interaction studies, which provided more opportunity for mutations to arise and segregate across our experimental strains. These extra mating and sporulation steps are especially relevant because meiosis can exhibit elevated rates of mutation at sites of recombination (Rattray *et al.* 2015).

Regarding the specific mutations we observed in our strains, despite the fact that aneuploidy typically results in a growth defect, in some cases it can provide an advantage during stress and even help overcome the loss of a gene (Vernon *et al.* 2008; Pavelka *et al.* 2010; Yona *et al.* 2012; Liu *et al.* 2015). In our experiments we find that chromosome V duplication was commonly observed in strains resulting from both the first and second rounds of mating and haploid selection. The magic marker locus we used to select for haploids of a desired mating type (*can1Δ*::*MFA1pr-HIS3-MFα1pr-LEU2*), is located on chromosome V. It functions by expressing His3 or Leu2 under a MATa-dependent or MATalpha-dependent promoter, respectively. Thus, an extra copy of the magic marker locus created by duplication may produce more His3 or Leu2, providing a benefit during selection on media lacking histidine or leucine. In our pooled growth assays, however, we found that chromosome V duplication typically correlates with a decrease in fitness, suggesting that the selective advantage only occurs during strain construction (Figure 3C). However, plating serial dilutions of our sequenced double mutants onto YPD, and the medium used for their selection, revealed no consistent growth advantage for strains carrying the duplication (Figure S4), though such an advantage could be confounded by other segregating variation. We lacked the statistical power to determine if rarer aneuploidies or SNPs also correlate with fitness. Of particular concern is that some of these variants may be deletion-specific suppressor mutations; these have been found in the deletion collection (Teng *et al.* 2013), and have been found to establish after only a few generations of growth (Szamecz *et al.* 2014). In our sequencing, we observed five cases of an aneuploidy of a chromosome rescuing a gene deletion.

There are several potential solutions to reduce the amount of segregating genetic variation and *de novo* mutations during genetic interaction screens. To address the common chromosome V aneuploidy we observe (in 41% of sequenced strains), one potential solution would be to include, at the magic marker locus, a gene that can be tolerated in no more than two copies in the haploid (including one copy at the endogenous locus), such as *CDC14* (Moriya *et al.* 2006). Alternatively, using the *STE2/STE3*-driven magic marker, or having the construct on a plasmid rather than it being integrated in the genome, may reduce the rates of accumulation of chromosome V aneuploidy. It is clear that not all genetic variation could be controlled in this manner. A possible alternative approach to minimize the generation of confounding genetic variation would be to minimize the number of generations deletion strains undergo between the introduction of the gene deletion(s) and the fitness measurements. For example, inducible CRISPR/Cas9 systems that knockdown selected gene targets are available (Gilbert *et al.* 2013; Mans *et al.* 2015; Senturk *et al.* 2015; Smith *et al.* 2016), and these could be used in conjunction with iSeq, by integrating gRNAs at the same time and location as barcodes in order to generate inducible double knockdowns. This strategy could also be employed to search for interactions that include essential genes.

We envision that iSeq can be used in the future to measure interactions between large groups of gene deletions across different experimental growth conditions. However, scaling up will require modifications to our strain generation protocol, such that a double barcode no longer marks a single clonal instance of a genotype. To achieve this, one could relatively easily place iSeq barcodes in the deletion collection library using the synthetic genetic array technology (Tong and Boone 2007) in combination with robotics. Double-barcode, double-deletion strains could then be generated via another round of robot-assisted paired mating and haploid selection. Because these protocols do not use single cell bottlenecks as we did here, strains generated from this modified protocol would likely consist of many segregants, and fitnesses and GI scores would be an aggregate measurement of these segregant pools. While segregant pool measurements may be likely to be more comparable with previous studies, they would not reveal the possible confounding influence of segregating variation and *de novo* mutations as we did here. Thus, employing iSeq as we described it here, would provide a valuable secondary method to validate that interactions discovered by high-throughput methods are robust. These two complementary approaches illustrate iSeq’s flexibility. Indeed, the iSeq technology has many potential applications beyond screening for interactions between gene deletions, and could in theory be used to screen, in multiple growth conditions, for interactions between naturally occurring genetic variation or engineered genetic constructs.

## Supplementary Material

Supplemental material is available online at www.g3journal.org/lookup/suppl/doi:10.1534/g3.116.034207/-/DC1.
