# Supplementary figures and images for: iSeq: A New Double-Barcode Method for Detecting Dynamic Genetic Interactions in Yeast
Source: G3 (Bethesda). 2016 Nov 7;7(1):143–53. doi: 10.1534/g3.116.034207 (PMC5217104; doi:10.1534/g3.116.034207)

Figure S1

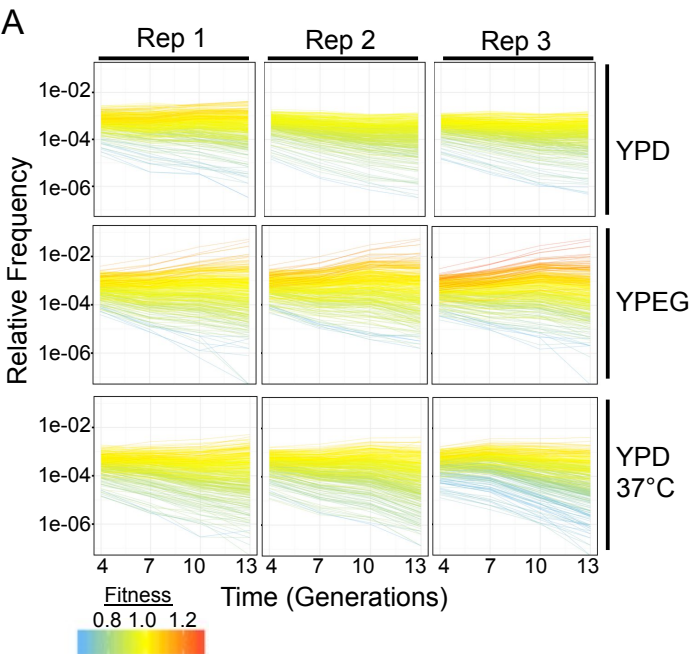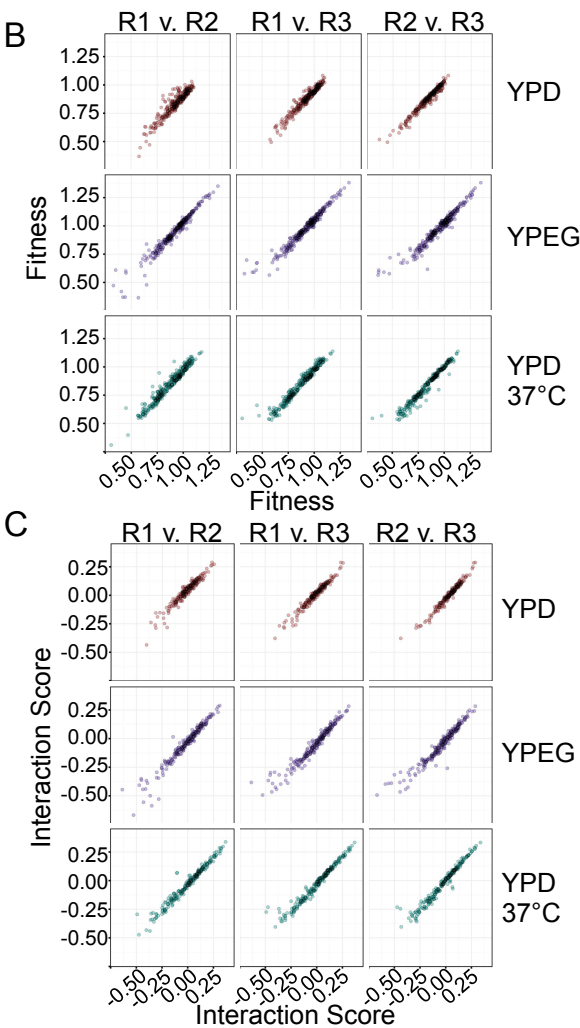

Supplement: Supplementary file 1 [file 143FigureS1.pdf]

Figure S2

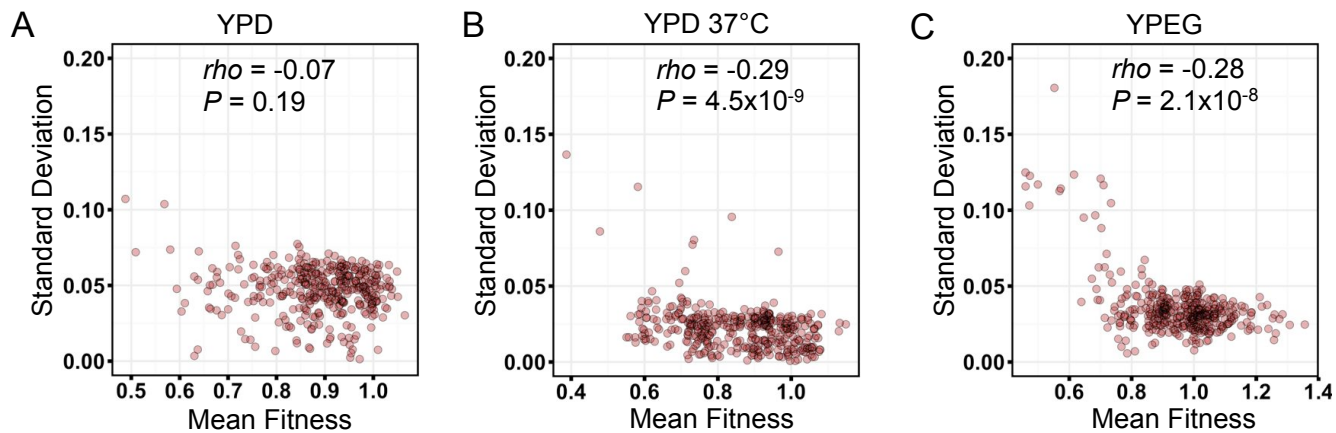

Supplement: Supplementary file 2 [file 143FigureS2.pdf]

Figure S3

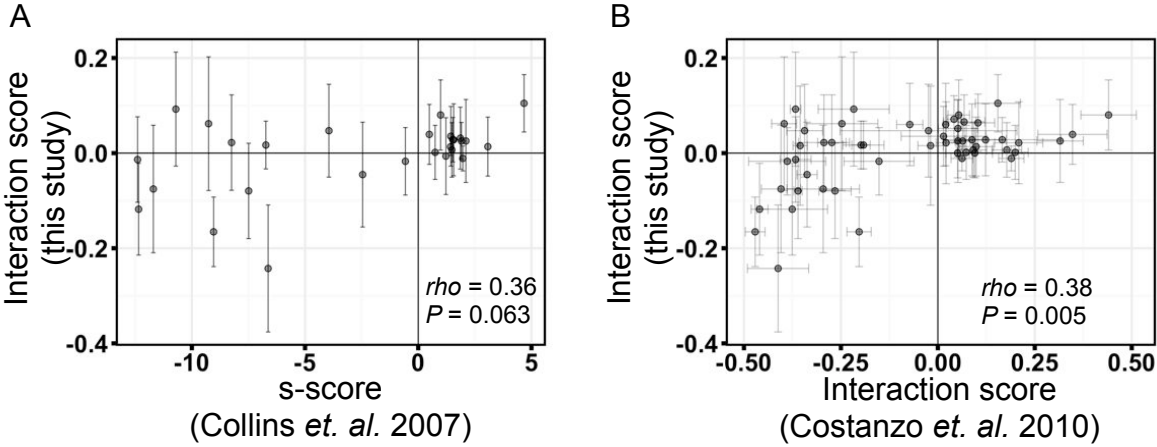

Supplement: Supplementary file 3 [file 143FigureS3.pdf]

Figure S4

*arp6Δ pho23Δ*

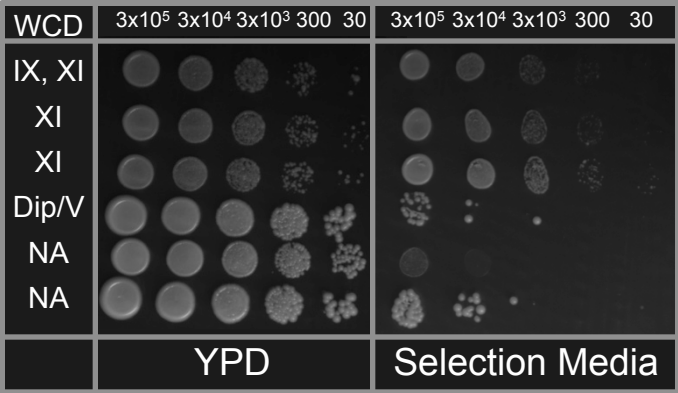

*sin3Δ dep1Δ*

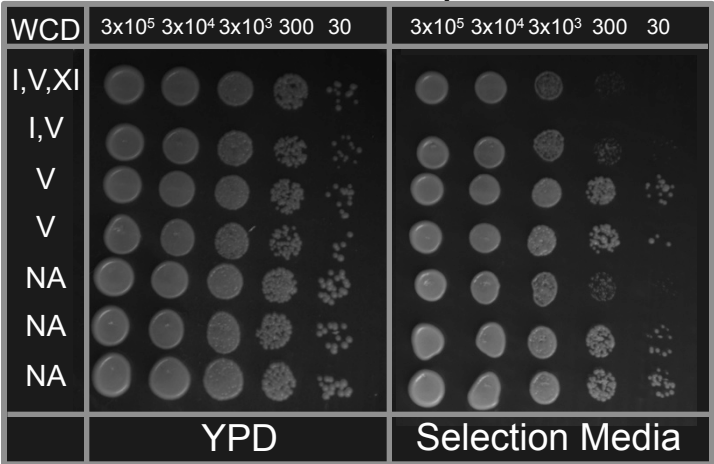

*sds3Δ pho23Δ*

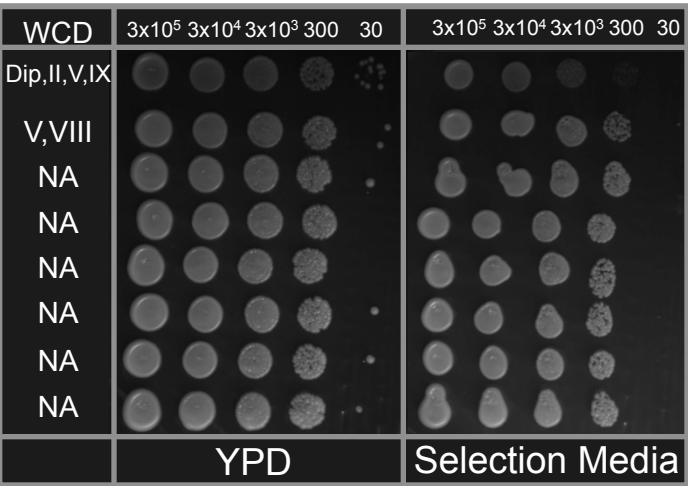

*rpd3Δ pho23Δ*

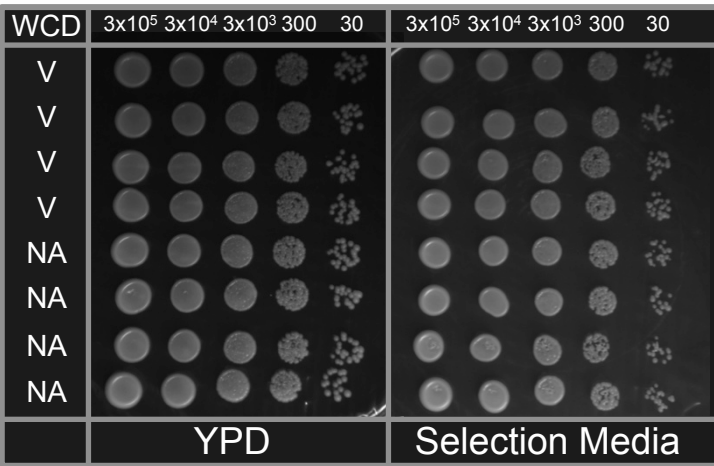

*sin3Δ sds3Δ*

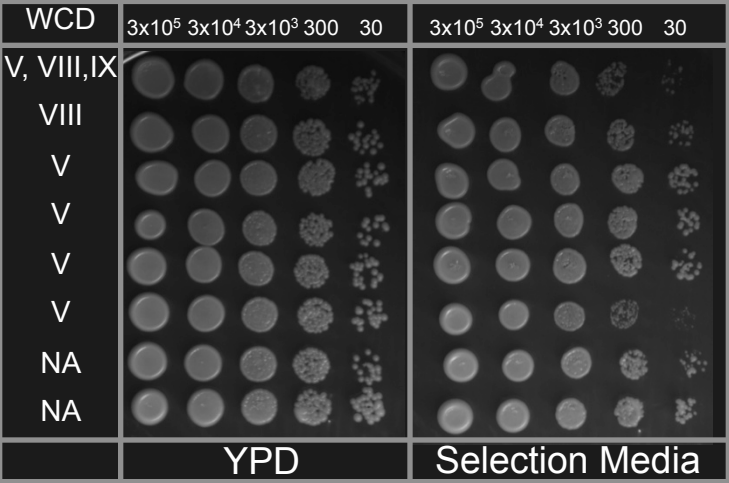

Supplement: Supplementary file 4 [file 143FigureS4.pdf]
