# Supplementary material for: iSeq: A New Double-Barcode Method for Detecting Dynamic Genetic Interactions in Yeast
Source: G3 (Bethesda). 2016 Nov 7;7(1):143–53. doi: 10.1534/g3.116.034207 (PMC5217104; doi:10.1534/g3.116.034207)
Supplement: Supplementary file 5 [file 143FileS1.pdf]

Supplemental Information

Table of Contents

Section	Page(s)
Table S1	3-5
Figures S1-S4	6-9
Supplemental Figure Legends	10
Supplemental Table Titles	10
Supplemental Materials and Methods	11-15
Primers	16
Strains	17
Plasmids	18-22
Supplemental References	23
Detailed Protocol for iSeq	24-47

Table S1

Gene Pair	Type	Publi-cations	Published Quantitative GI Scores	Published Non-Quantitative GIs
arp6Δsin3Δ	Negative	4	ε = -0.4711[1]<br>s = -9.0381[2]	Synthetic Lethal[3]<br>Synthetic Growth Defect[4]
sin3Δsnt1Δ	Negative	3	ε = -0.3606[1]<br>s = -7.4923[2]<br>s = -3.8016[5]	
arp6Δdep1Δ	Negative	3	ε = -0.3964[1]<br>s = -9.2601[2]	Synthetic Lethality[6]
arp6Δsap30Δ	Negative	5	ε = -0.4597[1]<br>s = -12.3508[2]	Synthetic Lethality[6]<br>Synthetic Growth Defect[3,4]
arp6Δsds3Δ	Negative	3	ε = -0.367[1]<br>s = -10.7073[2]	Synthetic Growth Defect[4]
arp6Δrpd3Δ	Negative	4	ε = -0.412[1]<br>s = -6.631[2]	Synthetic Lethal[3]<br>Synthetic Growth Defect[4]
arp6Δpho23Δ	Negative	5	ε = -0.4046[1]<br>s = -11.7012[2]	Synthetic Growth Defect[3,4,6]
dep1Δsnt1Δ	Negative	3	ε = -0.3668[1]<br>s = -12.3968[2]	Synthetic Lethality[6]
arp6Δsnt1Δ	Negative	2	ε = -0.3884[1]	Synthetic Growth Defect[4]
snt1Δsds3Δ	Negative	3	ε = -0.2734[1]<br>s = -8.2462[2]<br>s = -8.389[5]	
snt1Δdgk1Δ	Negative	1	ε = -0.3551[1]	
snt1Δpho23Δ	Negative	3	ε = -0.3436[1]<br>s = -3.947[2]<br>s = -3.5671[5]	
snt1Δrpd3Δ	Negative	2	ε = -0.3372[1]	Phenotypic Enhancement[7]
sap30Δsnt1Δ	Negative	2	ε = -0.1983[1]<br>s = -6.7316[2]	
sin3Δsds3Δ	Positive	1	ε = 0.1777[1]	

Table S1

Gene Pair	Type	Publi-<br>cations	Published<br>Quantitative GI Scores	Published<br>Non-Quantitative GIs
dgk1Δsin3Δ	Positive	1		Phenotypic Suppression[7]
sin3Δpho23Δ	Positive	3	s = 2.9662[5]<br>s = 2.0226[8]	Positive Genetic[9]
sin3Δrpd3Δ	Positive	2	s = 4.6737[2]	Positive Genetic[9]
sin3Δsap30Δ	Positive	3	ε = 0.1894[1]<br>s = 2.1593[10]	Positive Genetic[9]
dep1Δrpd3Δ	Positive	1	s = 3.0745[2]	
rpd3Δpho23Δ	Positive	1		Positive Genetic[9]
dgk1Δsds3Δ	Positive	1	ε = 0.2087[1]	
sap30Δsds3Δ	Positive	2	ε = 0.1995[1]<br>s = 2.7149[5]	
dep1Δpho23Δ	Positive	1	ε = 0.1659[1]	
sds3Δpho23Δ	Positive	2	ε = 0.3153[1]<br>s = 2.8751[5]	
dep1Δsds3Δ	Positive	1	ε = 0.4403[1]	
sds3Δrpd3Δ	Positive	2	ε = 0.3473[1]	Phenotypic Suppression[11]
sap30Δpho23Δ	Positive	2	s = 2.532[5]	Positive Genetic[9]
rpd3Δsap30Δ	Positive	1		Positive Genetic[9]
sin3Δdep1Δ	None			
dep1Δsap30Δ	None			
dgk1Δdep1Δ	None			
dgk1Δarp6Δ	None			
dgk1Δpho23Δ	None			
dgk1Δsap30Δ	None			
dgk1Δrpd3Δ	None			

Source Publications:

1. Costanzo, M. *et al.* The genetic landscape of a cell. *Science* **327,** 425–431 (2010).

Table S1

2. Collins, S. R. *et al.* Functional dissection of protein complexes involved in yeast chromosome biology using a genetic interaction map. *Nature* **446,** 806–810 (2007).
3. Lin, Y.-Y. *et al.* A comprehensive synthetic genetic interaction network governing yeast histone acetylation and deacetylation. *Genes Dev.* **22,** 2062–2074 (2008).
4. Krogan, N. J. *et al.* A Snf2 family ATPase complex required for recruitment of the histone H2A variant Htz1. *Mol. Cell* **12,** 1565–1576 (2003).
5. Zheng, J. *et al.* Epistatic relationships reveal the functional organization of yeast transcription factors. *Mol. Syst. Biol.* **6,** 420 (2010).
6. Tong, A. H. Y. *et al.* Global mapping of the yeast genetic interaction network. *Science* **303,** 808–813 (2004).
7. Beltrao, P. *et al.* Evolution of phosphoregulation: comparison of phosphorylation patterns across yeast species. *PLoS Biol.* **7,** e1000134 (2009).
8. Bandyopadhyay, S. *et al.* Rewiring of genetic networks in response to DNA damage. *Science* **330,** 1385–1389 (2010).
9. Breslow, D. K. *et al.* A comprehensive strategy enabling high-resolution functional analysis of the yeast genome. *Nat. Methods* **5,** 711–718 (2008).
10. Wilmes, G. M. *et al.* A genetic interaction map of RNA-processing factors reveals links between Sem1/Dss1-containing complexes and mRNA export and splicing. *Mol. Cell* **32,** 735–746 (2008).
11. Torres-Machorro, A. L. & Pillus, L. Bypassing the requirement for an essential MYST acetyltransferase. *Genetics* **197,** 851–863 (2014).

Figure S1

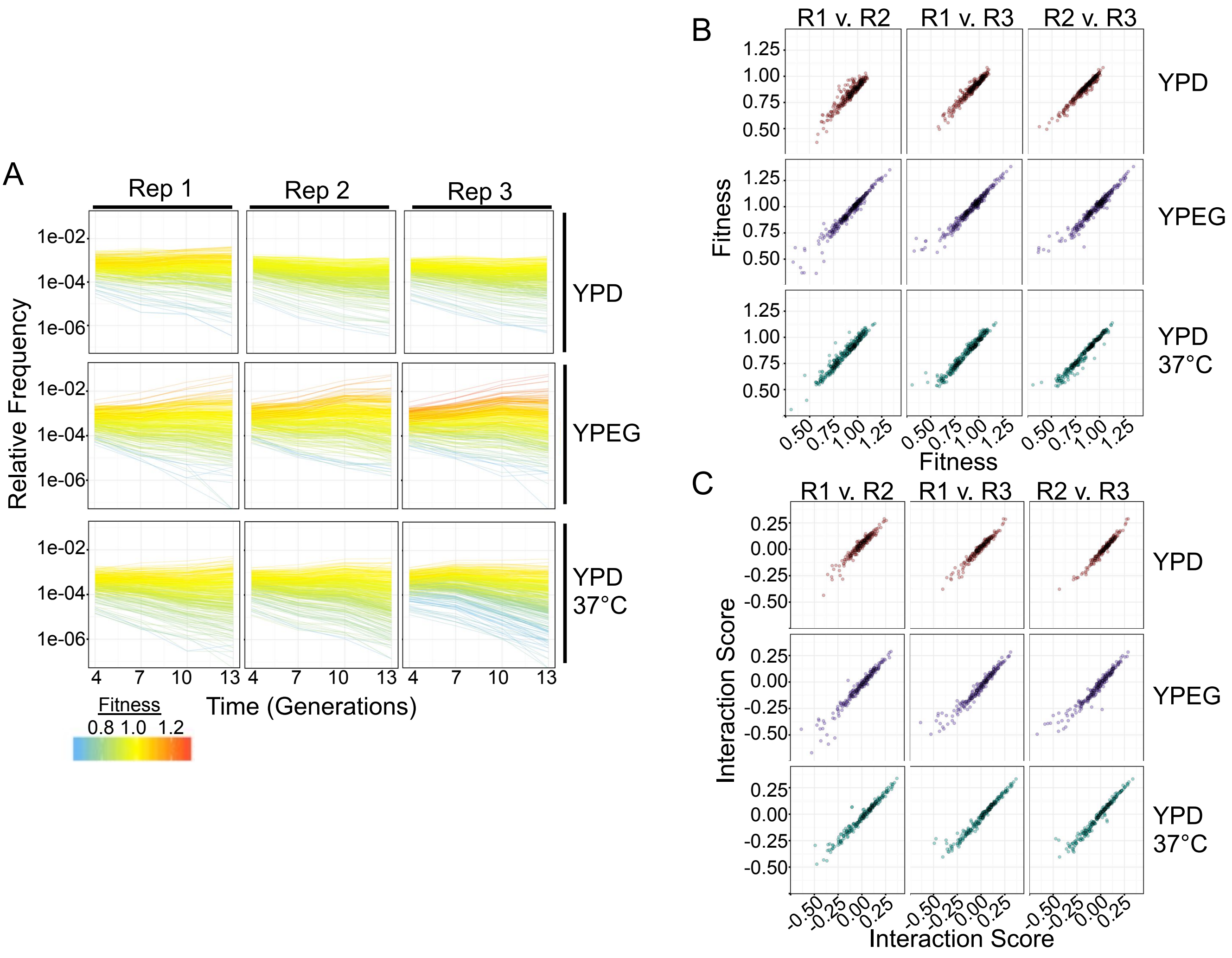

A
Rep 1
Rep 2
Rep 3
1e-02
1e-04
1e-06
Relative Frequency
YPD
YPEG
YPD 37°C
4 7 10 13
Time (Generations)
Fitness
0.8 1.0 1.2
B
R1 v. R2
R1 v. R3
R2 v. R3
1.25
1.00
0.75
0.50
Fitness
C
Interaction Score
0.25
0.00
-0.25
-0.50

Figure S2

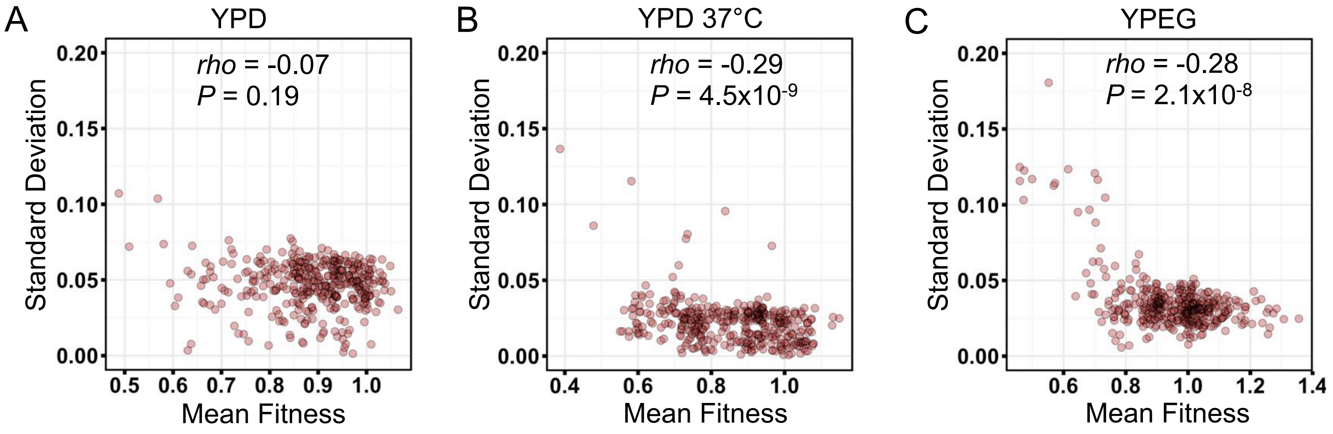
A
YPD
rho = -0.07
P = 0.19
Standard Deviation
Mean Fitness
B
YPD 37°C
rho = -0.29
P = 4.5x10-9
Standard Deviation
Mean Fitness
C
YPEG
rho = -0.28
P = 2.1x10-8
Standard Deviation
Mean Fitness

Figure S3

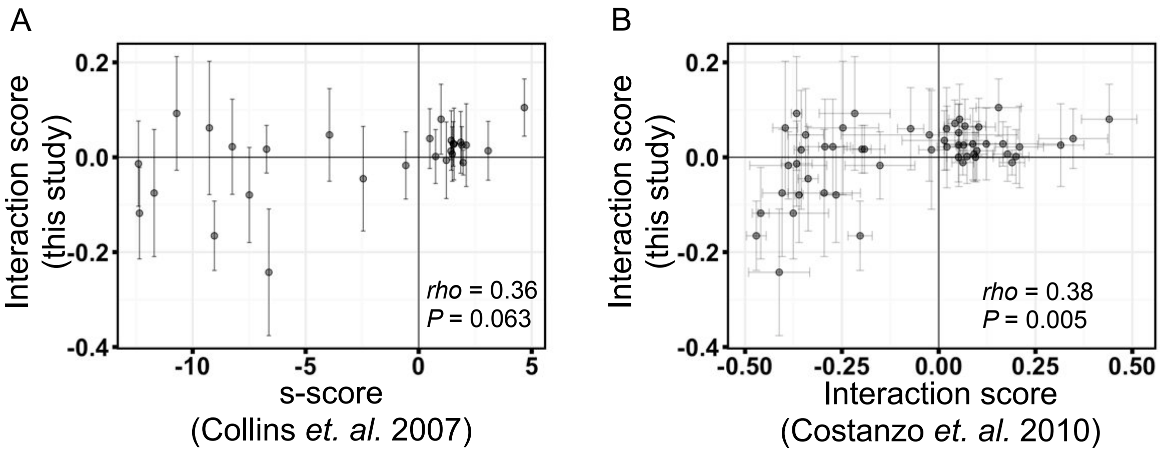
A
0.2
0.0
-0.2
-0.4
Interaction score
(this study)
-10
-5
0
5
s-score
(Collins et. al. 2007)
rho = 0.36
P = 0.063
B
0.2
0.0
-0.2
-0.4
Interaction score
(this study)
-0.50
-0.25
0.00
0.25
0.50
Interaction score
(Costanzo et. al. 2010)
rho = 0.38
P = 0.005

Figure S4

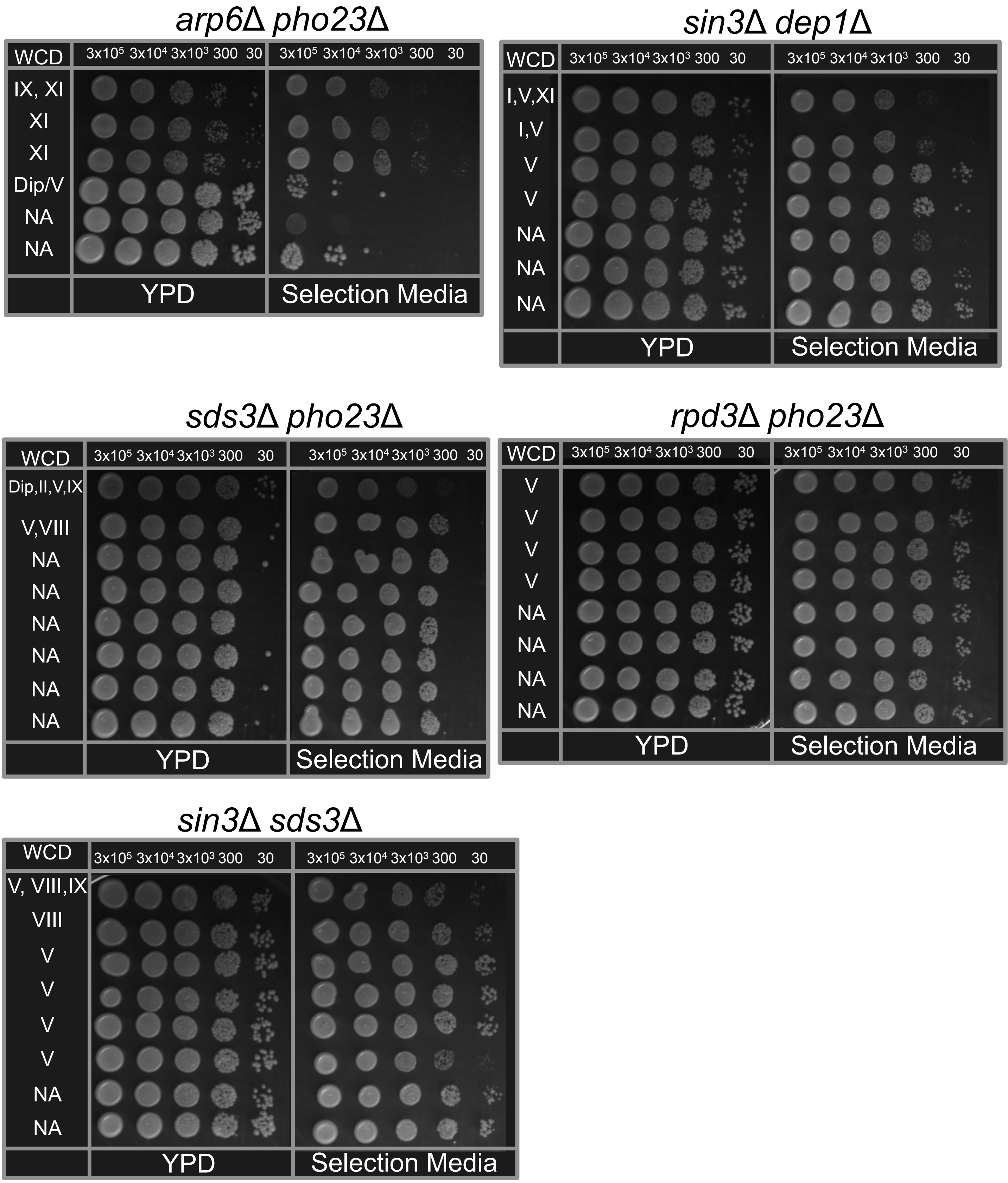

arp6Δ pho23Δ
WCD
3x10^5 3x10^4 3x10^3 300 30
IX, XI
XI
XI
Dip/V
NA
NA
YPD
Selection Media
sin3Δ dep1Δ
WCD
3x10^5 3x10^4 3x10^3 300 30
I,V,XI
I,V
V
V
NA
NA
NA
YPD
Selection Media
sds3Δ pho23Δ
WCD
3x10^5 3x10^4 3x10^3 300 30
Dip,II,V,IX
V,VIII
NA
NA
NA
NA
NA
NA
YPD
Selection Media
rpd3Δ pho23Δ
WCD
3x10^5 3x10^4 3x10^3 300 30
V
V
V
V
NA
NA
NA
NA
YPD
Selection Media
sin3Δ sds3Δ
WCD
3x10^5 3x10^4 3x10^3 300 30
V, VIII,IX
VIII
V
V
V
V
NA
NA
YPD
Selection Media

Supplemental Figure Legends:

Figure S1. A) Relative frequency trajectories for all conditions and replicates. Each line is an individual double barcode strain. Colors indicate the fitness estimate of each strain. **B,C)** Scatter plots of fitnesses **(B)** and interaction scores **(C)** between each of three biological replicates in each of three experimental conditions. Each point is an individual strain.

Figure S2. Scatter plot of the mean and the standard deviation of fitness estimates made in three replicate cultures in YPD (**A**), YPD 37°C (**B**) and YPEG (**C**). Each point is an individual strain. Spearman's *rho* and *P*-value are given on the plots.

Figure S3. Scatter plot of iSeq interaction score estimates and s-scores reported by Collins *et. al.* 2007 (**A**) and interaction scores reported in Costanzo *et. al.* 2010 (**B**). Each point is a gene pair. Error bars on our measurements represent the standard deviation across 4-8 strains carrying the corresponding gene deletions. In panel **B**, two scores were published for a subset of gene pairs. Error bars on Costanzo *et. al.* data are the standard deviations reported on the estimates.

Figure S4. Spot assays for F0 strains from the 5 double gene deletion genotypes that were whole-genome sequenced. Overnight cultures were inoculated from single colonies and diluted to $1x10^8$ cells/mL. Four additional serial dilutions (1:10) were performed, and 3ul of each dilution was plated to either YPD or the media used to select haploid double deletion strains carrying the iSeq double barcode (1.7g/L Difco yeast nitrogen base without amino acids and ammonium sulfate, 1g/L monosodium glutamic acid, 2% glucose, Nat, G418, –Ura, –Lys, –Leu, –Ade, and –Arg). The number of cells plated (columns), and the whole chromosome duplication events identified in each strain (rows), are labeled on each panel.

Supplemental Table Titles:

Table S1. Published genetic interactions for each of the 36 double gene deletion genotypes used in this study. Quantitative scores are either an SGA score (ε), or an S score (*s*). See references for sources of reported interactions. All published interactions were found in the BioGRID repository.
Table S2. (Separate excel file) Fitness and interaction score estimates for each individual strain in each replicate of each experimental condition.
Table S3. (Separate excel file) Expanded summary of whole genome sequencing data.
Table S4. (Separate excel file) Interaction score estimates called as significant using 95% confidence intervals.
Table S5. (Separate excel file) Sequencing counts for each strain, at each time point, in each culture, and each strain's doubling time and fitness estimates from Optical Density growth curve fitness assay.

Supplemental Materials and Methods

Yeast barcode library construction

Outline of protocol used to generate yeast barcode libraries is pictured in Diagram 1.

Construction of starting strains into which plasmid barcode libraries are introduced:

To generate the starting strain for the first yeast barcode library, *Gal-Cre-NatMX* was amplified from the plasmid pBAR1 (Levy et al., 2015) using the primers PEV8 and PEV9. The 5' ends of PEV8 and PEV9 are identical to downstream and upstream sequences of the dubious open reading frame (ORF) YBR209W, respectively. This PCR product was then transformed into BY4742 (Gietz and Schiestl, 2007), to replace the YBR209W ORF such that both the *Gal-Cre* and *NatMX* genes are transcribed on the Watson strand, yielding the strain SHA319. The starting strain for the complementary library was generated similarly by first using primers P102 and P103 to amplify *Gal-Cre-NatMX* from pBAR1.This PCR product was then transformed into BY4742 to replace the YBR209W ORF such that both the *Gal-Cre* and *NatMX* genes are on the Crick strand, yielding the strain SHA321. Each strain was verified by PCR for successful integration.

Next, the magic marker construct, *MFA1pr-HIS3-MFα1pr-LEU2* (Tong et al., 2004), was amplified from DNA extracted from a haploid derivative of UCC8600 (Lindstrom and Gottschling, 2009) using the published primers P14 and P15 (Tong et al., 2004). The resulting fragment was used to replace *CAN1* in SHA319 and SHA321 via homologous recombination. This insertion allows for selection of either *MATa* or *MAT*α haploids via growth on synthetic complete (SC) media containing canavanine and lacking either histidine or leucine respectively. Integration of the magic marker at the *CAN1* locus in SHA319 and SHA321 created strains HR28 and HR27 respectively; correct integration was verified by PCR.

Generation of plasmid barcode libraries and introduction into yeast starting strains:

The plasmid library pBAR3-L1 was previously generated (~500,000 barcodes) (Levy et al., 2015) and carries lox71 (Albert et al., 1995), a DNA barcode (20 random nucleotides), an artificial intron, the 5' half of *URA3*, and *HygMX*. A second plasmid library (~75,000 barcodes), L001, was constructed using the same protocol as the pBAR3-L1, and contains lox66, a DNA barcode, an artificial intron, the 3' half of *URA3*, and *HygMX*. In order to introduce the remaining components of the double barcoding system to the YBR209W locus carrying *Gal-Cre-NatMX*, pBAR3-L1 and L001 were each digested with SacI and XhoI, PCR column purified, and transformed into HR28 and HR27, respectively.

For each transformed yeast strain, 288 clones (with presumably different barcodes) were picked. All clones were tested for successful replacement of *NatMX* with *HygMX* by growth on YPD-Hygromycin (Hyg) and YPD-Nourseothricin (Nat) plates. All clones were determined to be competent to mate and recombine at the loxP locus by replica plating mated pairs onto SC+Gal–Ura. Gal-Cre induced recombination between lox66 and lox71 is irreversible, and brings the two barcodes onto the same stretch of DNA to form an intron of a complete and functional *URA3* gene (Fig 1A). Additionally, 5 clones total were verified for proper integration of the barcode construct via PCR and Sanger sequencing.

Double-barcoded double-deletion yeast strain generation

All steps used to generate double-barcode double-deletion yeast strains are outlined in Diagram 2.

Obtaining single gene deletion strains:

Gene deletion strains from the diploid heterozygous deletion collection (Pan et al., 2004; Tong et al., 2001) were obtained for the following genes and dubious ORFs: *ARP6*, *SAP30*, *SDS3*, *PHO23*, *SIN3*, *DGK1*, *SNT1*, *DEP1*, *RPD3*, YHR095W and YFR054C. Deletions in these strains are marked by KanMX and have the following genotype: *MATa/MAT*α *ura3*Δ0 *leu2*Δ0 *his3*Δ1 *LYS2*/*lys2*Δ0 *MET15*/*met15*Δ0 *CAN1*/*can1*Δ::*LEU2-MFA1pr-HIS3*. All 11 deletion strains were streaked from frozen stocks to single colonies and sporulated in 1% potassium acetate, 0.005% zinc acetate supplemented with uracil, histidine and leucine. Following tetrad dissection, haploids were genotyped on plates and strains carrying the *KanMX* marked gene deletion with the following genotype were chosen: *MATa ura3*Δ0 *leu2*Δ0 *his3*Δ1.

In subsequent steps, in order to be able to select strains carrying two gene deletions, strains with an alternative drug marker at each gene deletion were needed. To obtain these alternatively marked deletion strains,

each of the 11 haploid deletion strains was transformed, to replace the *KanMX* marker with the *NatMX* marker. DNA for the transformation was generated by digesting the pBAR1 plasmid with the FspI restriction enzyme and gel purifying.

Mating gene deletion strains to strains from yeast barcode libraries:

Each of the 11 deletion strains marked with *KanMX* was mated to two unique strains from the 5' barcode construct carrying yeast library. *NatMX* marked deletion strains were each mated to two strains from the 3' barcode construct carrying yeast library. All matings were carried out by first streaking parental strains from frozen stocks to single colonies, and then overlaying parental strains on patches on YPD plates and growing overnight at room temperature. Mating plates were replica plated to YP + 100 μg/mL Hygromycin (Hyg) + [100 μg/mL Nourseothricin (Nat) or 200 μg/mL G418, depending on deletion marker] to select for diploids. Diploid strains carrying a deletion and the barcode construct at YBR209W were sporulated and plated for haploid single colonies on selectable medium (1.7g/L Difco yeast nitrogen base without amino acids and ammonium sulfate, 1g/L monosodium glutamic acid, 2% glucose, (Nat or G418, depending on deletion marker), Hyg, 60 μg/mL canavanine, –Lys, –Arg and [–Leu or –His, depending on desired mating type]. In order to obtain strains with complementary mating types for the second round of matings, *MATa* strains were selected from the matings derived from *KanMX* marked parents and *MAT*α from those of *NatMX* marked parents. After selecting haploids, the identity of each gene deletion was verified by PCR and Sanger sequencing the DNA barcode at the deletion site (Winzeler et al., 1999). In each strain, the sequence of the DNA barcode in the construct at YBR209W was also determined by PCR and Sanger sequencing.

Mating strains carrying a gene deletion and a barcode construct with strains carrying a gene deletion and a complementary barcode construct:

To obtain strains carrying two gene deletions and both complementary barcode constructs, each singly barcoded deletion strain was streaked to a single colony on YPD. Strains were mated at room temperature on YPD overnight. After mating, plates were replica plated to YPD+Nat+G418 and grown at 30°C overnight to select for diploids. Strains were next replica plated to SC + 2% Galactose –Ura and grown at 30°C for 2 days to induce Cre-mediated recombination at the barcode locus. These cells were next grown O/N in 1 mL YPD at 30°C. 125 μL of this culture were resuspended in sporulation media, and cells were allowed to sporulate spinning at RT for 3 days. After sporulation, unsporulated diploids were digested using zymolyase as described (Herman and Rine, 1997) before plating to selectable media (1.7g/L Difco yeast nitrogen base without amino acids and ammonium sulfate, 1g/L monosodium glutamic acid, 2% glucose, Nat, G418, –Ura, –Lys, –Leu, –Ade, and –Arg) for single haploid colonies.

Picking colonies from haploid selection plates:

The number of colonies per plate varied between 1 and ~300. In the majority of cases, a single colony of average size was picked for each strain. In 31 cases high heterogeneity was observed in colony sizes within strains carrying double deletions with a large fitness defect (Costanzo et al., 2010). In these cases, small colonies were picked in order to avoid using strains containing suppressor mutations. In cases where segregants grew at a high density on plates, differences in colony sizes could not be easily distinguished, and a random colony was picked. All strains were verified for correct genotype on plates, and, in a subset, the double barcode sequence was verified by Sanger sequencing.

Fitness and genetic interaction estimates

Strain dropout:

The majority of strains were present at each of the four time points, and for each of these strains, fitness estimates were made from all available data. However, at time point four, there were 6 low fitness strains absent from the YPD pool, 32 were absent in YPEG, and 15 were absent in YPD 37°C. For all conditions, two of these strains were unobservable at even the first time point, indicating their absence in the starting pool at detectable frequencies. For the remaining strains, we used the maximum number of time points available to estimate fitness.

Single mutant fitness estimates used for interaction score calculation:

To estimate quantitative genetic interaction scores, estimates of fitness for single mutant strains are also required. In the pool, there were 12-16 strains representing each single mutant genotype. When calculating interaction scores, the single mutant fitness used was the average of the replicate strains carrying the same drug marker as the double mutant strain. For example, for a double mutant of the genotype *gene1*Δ::*KanMX*

*gene2*Δ::*NatMX*, the fitnesses of the four strains carrying the *gene1*Δ::*KanMX* allele and a deletion in a dubious ORF marked by *NatMX* were averaged and used as the fitness of the single mutant in *gene1*. Similarly, for the fitness of the single mutant in *gene2*, the fitness of the four strains carrying the *gene2*Δ::*NatMX* allele and a deletion in a dubious ORF marked by *KanMX* were averaged.

Calculation of 95% confidence interval around mean interaction score:

To call interaction scores as significantly positive or negative, a 95% confidence interval was calculated around the mean score from the 4-8 strains with identical pairs of gene deletions. This interval is defined as x ± E, where x is the sample mean. The margin of error, $E = t_c \times \frac{s}{\sqrt{n}}$, where s is the sample standard deviation, n is the number of samples, and t_c is the critical value taken from the *t*-distribution using a confidence level of 95%. An interaction was then called as significant depending on whether a score of zero fell within the calculated confidence interval.

Whole-genome sequencing

Calling SNPs/indels from raw GATK .vcf files

Raw .vcf files for each sequenced strain were filtered for quality using a quality threshold value of 350. Next, a list of 169 ancestral variants was generated and included any variant that was observed at least once in either of the two background control strains (BY4741 and BY4742, each sequenced in duplicate). If any of these 169 ancestral variants were called in F0, F1 or F2 experimental strains, they were removed. Next, for each of the 39 double gene deletion and 8 double dORF deletion F2 strains, the remaining variants were each visually verified using the `tview` function in samtools. Variants in regions with poor mapping quality, or low coverage were removed and a final matrix of 73 verified SNPs/indels and their presence across the 47 F2 strains was generated. Finally, the origin of each SNP/indel in each F2 strain was determined by observing whether the variant was present in the related F1 and F0 parental strains via its presence in the F0 or F1 strain's filtered .vcf file, or, to prevent false negatives, by visual inspection via samtools `tview`.

Diagram 1

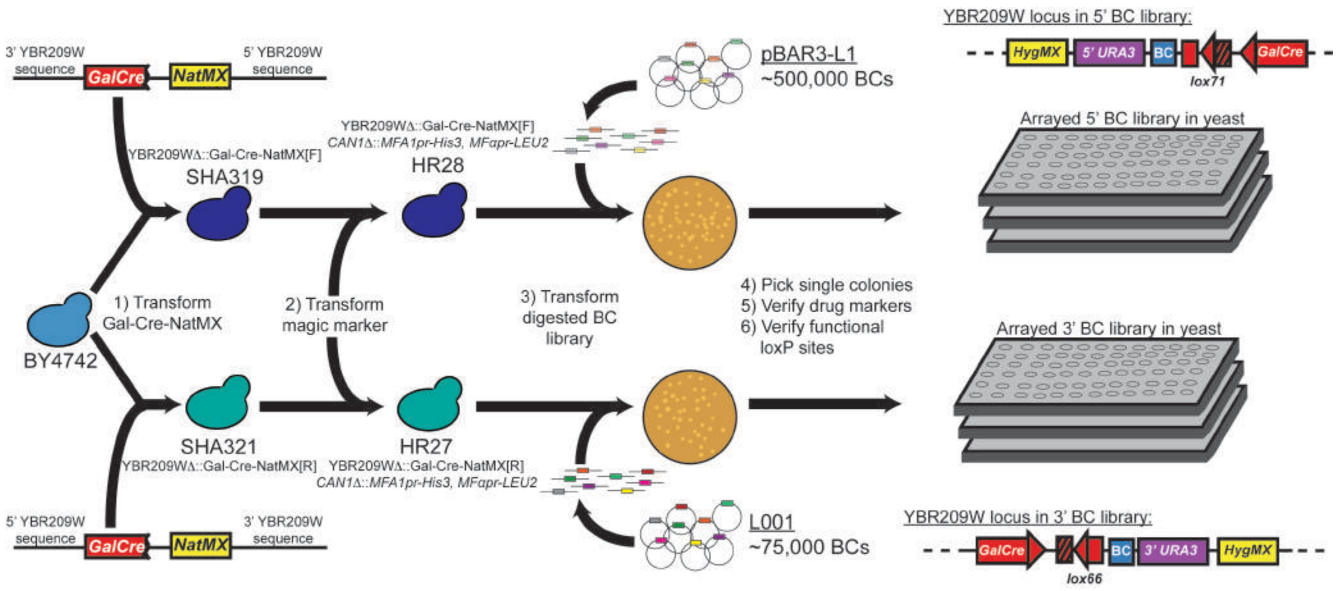

YBR209W locus in 5' BC library:
HygMX
5' URA3
BC
lox71
GalCre
3' YBR209W sequence
GalCre
NatMX
5' YBR209W sequence
pBAR3-L1
~500,000 BCs
Arrayed 5' BC library in yeast
YBR209WΔ::Gal-Cre-NatMX[F]
CAN1Δ::MFA1pr-His3, MFαpr-LEU2
YBR209WΔ::Gal-Cre-NatMX[F]
SHA319
HR28
1) Transform
Gal-Cre-NatMX
2) Transform
magic marker
3) Transform
digested BC
library
4) Pick single colonies
5) Verify drug markers
6) Verify functional
loxP sites
BY4742
Arrayed 3' BC library in yeast
SHA321
HR27
YBR209WΔ::Gal-Cre-NatMX[R]
YBR209WΔ::Gal-Cre-NatMX[R]
CAN1Δ::MFA1pr-His3, MFαpr-LEU2
5' YBR209W sequence
GalCre
NatMX
3' YBR209W sequence
L001
~75,000 BCs
YBR209W locus in 3' BC library:
GalCre
lox66
BC
3' URA3
HygMX

Diagram 2

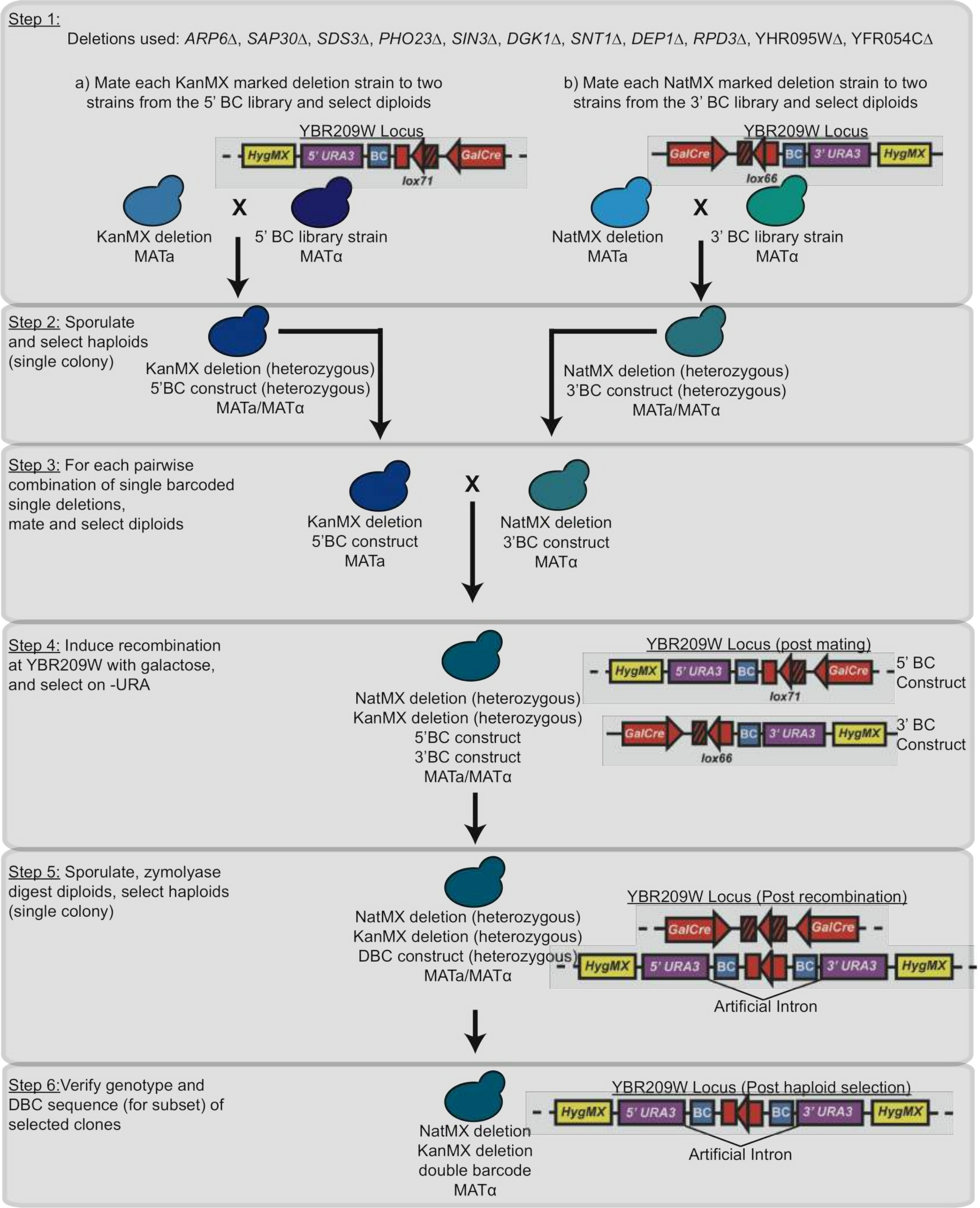

Step 1:
Deletions used: ARP6Δ, SAP30Δ, SDS3Δ, PHO23Δ, SIN3Δ, DGK1Δ, SNT1Δ, DEP1Δ, RPD3Δ, YHR095WΔ, YFR054CΔ
a) Mate each KanMX marked deletion strain to two strains from the 5' BC library and select diploids
b) Mate each NatMX marked deletion strain to two strains from the 3' BC library and select diploids
YBR209W Locus
HygMX
5' URA3
BC
GalCre
lox71
YBR209W Locus
GalCre
BC
3' URA3
HygMX
lox66
KanMX deletion
MATa
X
5' BC library strain
MATα
NatMX deletion
MATa
X
3' BC library strain
MATα
Step 2: Sporulate and select haploids (single colony)
KanMX deletion (heterozygous)
5'BC construct (heterozygous)
MATa/MATα
NatMX deletion (heterozygous)
3'BC construct (heterozygous)
MATa/MATα
Step 3: For each pairwise combination of single barcoded single deletions, mate and select diploids
KanMX deletion
5'BC construct
MATa
X
NatMX deletion
3'BC construct
MATα
Step 4: Induce recombination at YBR209W with galactose, and select on -URA
NatMX deletion (heterozygous)
KanMX deletion (heterozygous)
5'BC construct
3'BC construct
MATa/MATα
YBR209W Locus (post mating)
HygMX
5' URA3
BC
GalCre
lox71
5' BC Construct
GalCre
BC
3' URA3
HygMX
lox66
3' BC Construct
Step 5: Sporulate, zymolyase digest diploids, select haploids (single colony)
NatMX deletion (heterozygous)
KanMX deletion (heterozygous)
DBC construct (heterozygous)
MATa/MATα
YBR209W Locus (Post recombination)
GalCre
GalCre
HygMX
5' URA3
BC
BC
3' URA3
HygMX
Artificial Intron
Step 6:Verify genotype and DBC sequence (for subset) of selected clones
NatMX deletion
KanMX deletion
double barcode
MATα
YBR209W Locus (Post haploid selection)
HygMX
5' URA3
BC
BC
3' URA3
HygMX
Artificial Intron

Primers

PEV8: 5′GTTCTTTGCTTTTTTTTCCCCAACGACGTCGAACACATTAGTCCTACGCACTTAACTTCGCATCTG
PEV9: 5′GCTTGCGCTAACTGCGAACAGAGTGCCCTATGAAATAGGGGAATGCATATCATACGTAATGCTCAACCTT
P102: 5′GCTTGCGCTAACTGCGAACAGAGTGCCCTATGAAATAGGGGAATGCGCACTTAACTTCGCATCTG
P103: 5′GTTCTTTGCTTTTTTTTCCCCAACGACGTCGAACACATTAGTCCTACATATCATACGTAATGCTCAACCTT
P14: 5′GCGAACAGAGTAAACCGAA3′
P15: 5′GAAGGTCTGAAGGAGTTC3′

Strains

BY4742: *MAT*α *ura3*Δ0 *leu2*Δ0 *his3*Δ1 *lys2*Δ0
SHA319: *MAT*α *ura3*Δ0 *leu2*Δ0 *his3*Δ1 *lys2*Δ0 YBR209WΔ*::Gal-Cre-NatMX[W]*
SHA321: *MAT*α *ura3*Δ0 *leu2*Δ0 *his3*Δ1 *lys2*Δ0 YBR209WΔ*::Gal-Cre-NatMX[C]*
HR28: *MAT*α *ura3*Δ0 *leu2*Δ0 *his3*Δ1 *lys2*Δ0 YBR209WΔ*::Gal-Cre-NatMX[W] CAN1*Δ::*MFA1pr-HIS3-MFα1pr-LEU2*
HR27: *MAT*α *ura3*Δ0 *leu2*Δ0 *his3*Δ1 *lys2*Δ0 YBR209WΔ*::Gal-Cre-NatMX[C] CAN1*Δ::*MFA1pr-HIS3-MFα1pr-LEU2*

Plasmids

M207/pBAR1 (Published in Levy et al, 2015)

Key:

NNNN = Gal promoter
NNNN = Cre
NNNN = CYC1 Terminator
NNNN = *NatMX*

GAACGCGGCCGCCAGCTGAAGCTTCGTACGCTGCAGGTCGACGGATCCCCGGGTTAATTAAGGGCCCACAAGGTTTT
GCATTGAGGATAGTATAGAAGCAAGAATCATTGAATTACAGGAAAAAAAGGCAAATATGATTCATGCTACAATAAAC
CAAGATGAAGCTGCCATTAGCAGACTAACGCCAGCTGATTTACAGTTCTTATTCAATAACTAATATTTTATTCTCTT
ATTATATATTATTCTCGGAGTTTTTAAGTGACATCACCCGAAAAGAAGCTAAGTCTTTCTCCTAATTCATATTTAAT
TATTGTACATGGACATATCATACGTAATGCTCAACCTTAGCTAGCtagtacggattagaagccgccgagcgggtgac
agccctccgaaggaagactctcctccgtgcgtcctcgtcttcaccggtcgcgttcctgaaacgcagatgtgcctcgc
gccgcactgctccgaacaataaagattctacaatactagcttttatggttatgaagaggaaaaattggcagtaacct
ggccccacaaaccttcaaatgaacgaatcaaattaacaaccataggatgataatgcgattagttttttagccttatt
tctggggtaattaatcagcgaagcgatgatttttgatctattaacagatatataaatgcaaaaactgcataaccact
ttaactaatactttcaacattttcggtttgtattacttcttattcaaatgtaataaaagtatcaacaaaaaattgtt
aatatacctctatactttaacgtcaaggagaaaaaaccccggattctagaactagtggatcccccgggctgcaggaa
ttcgatatcaagcttatcgataccgtcgaggggcagagccgatcctgtacactttacttaaaaccattatctgagtg
ttaaatgtccaatttactgaccgtacaccaaaatttgcctgcattaccggtcgatgcaacgagtgatgaggttcgca
agaacctgatggacatgttcagggatcgccaggcgttttctgagcatacctggaaaatgcttctgtccgtttgccgg
tcgtgggcggcatggtgcaagttgaataaccggaaatggtttcccgcagaacctgaagatgttcgcgattatcttct
atatcttcaggcgcgcggtctggcagtaaaaactatccagcaacatttgggccagctaaacatgcttcatcgtcggt
ccgggctgccacgaccaagtgacagcaatgctgtttcactggttatgcggcggatccgaaaagaaaacgttgatgcc
ggtgaacgtgcaaaacaggctctagcgttcgaacgcactgatttcgaccaggttcgttcactcatggaaaatagcga
tcgctgccaggatatacgtaatctggcatttctggggattgcttataacaccctgttacgtatagccgaaattgcca
ggatcagggttaaagatatctcacgtactgacggtgggagaatgttaatccatattggcagaacgaaaacgctggtt
agcaccgcaggtgtagagaaggcacttagcctgggggtaactaaactggtcgagcgatggatttccgtctctggtgt
agctgatgatccgaataactacctgttttgccgggtcagaaaaaatggtgttgccgcgccatctgccaccagccagc
tatcaactcgcgccctggaagggatttttgaagcaactcatcgattgatttacggcgctaaggatgactctggtcag
agatacctggcctggtctggacacagtgcccgtgtcggagccgcgcgagatatggcccgcgctggagtttcaatacc
ggagatcatgcaagctggtggctggaccaatgtaaatattgtcatgaactatatccgtaccctggatagtgaaacag
gggcaatggtgcgcctgctggaagatggcgattagccattaacgcgtaaatgattgctataattatttgatatttat
ggtgacatatgagaaaggatttcaacatcgacggaaaatatgtagtgctgtctgtaagcactaatattcagtcgcca
gccgtcattgtcactgtaaagctgagcgatagaatgcctgatattgactcaatatccgttgcgtttcctgtcaaaag
tatgcgtagtgctgaacatttcgtgatgaatgccaccgaggaagaagcacggcgcggttttgctaaagtgatgtctg
agtttggcgaactcttgggtaaggttggaattgtcgacctcgagtcatgtaattagttatgtcacgcttacattcac
gccctccccccacatccgctctaaccgaaaaggaaggagttagacaacctgaagtctaggtccctatttattttttt
atagttatgttagtattaagaacgttatttatatttcaaatttttcttttttttctgtacagacgcgtgtacgcatg
taacattatactgaaaaccttgcttgagaaggttttgggacgctcgaaggctttaatttgcggccGGCGCGCCAGAT
CTGTTTAGCTTGCCTcGTCCCCGCCGGGTCACCCGGCCAGCGACATGGAGGCCCAGAATACCCTCCTTGACAGTCTT
GACGTGCGCAGCTCAGGGGCATGATGTGACTGTCGCCCGTACATTTAGCCCATACATCCCCATGTATAATCATTTGC
ATCCATACATTTTGATGGCCGCACGGCGCGAAGCAAAAATTACGGCTCCTCGCTGCAGACCTGCGAGCAGGGAAACG
CTCCCCTCACAGACGCGTTGAATTGTCCCCACGCCGCGCCCCTGTAGAGAAATATAAAAGGTTAGGATTTGCCACTG
AGGTTCTTCTTTCATATACTTCCTTTTAAAATCTTGCTAGGATACAGTTCTCACATCACATCCGAACATAAACAACC
ATGGGTACCACTCTTGACGACACGGCTTACCGGTACCGCACCAGTGTCCCGGGGGACGCCGAGGCCATCGAGGCACT
GGATGGGTCCTTCACCACCGACACCGTCTTCCGCGTCACCGCCACCGGGGACGGCTTCACCCTGCGGGAGGTGCCGG
TGGACCCGCCCCTGACCAAGGTGTTCCCCGACGACGAATCGGACGACGAATCGGACGACGGGGAGGACGGCGACCCG
GACTCCCGGACGTTCGTCGCGTACGGGGACGACGGCGACCTGGCGGGCTTCGTGGTCGTCTCGTACTCCGGCTGGAA
CCGCCGGCTGACCGTCGAGGACATCGAGGTCGCCCCGGAGCACCGGGGGCACGGGGTCGGGCGCGCGTTGATGGGGC
TCGCGACGGAGTTCGCCCGCGAGCGGGGCGCCGGGCACCTCTGGCTGGAGGTCACCAACGTCAACGCACCGGCGATC
CACGCGTACCGGCGGATGGGGTTCACCCTCTGCGGCCTGGACACCGCCCTGTACGACGGCACCGCCTCGGACGGCGA
GCAGGCGCTCTACATGAGCATGCCCTGCCCCTAATCAGTACTGACAATAAAAAGATTCTTGTTTTCAAGAACTTGTC
ATTTGTATAGTTTTTTTATATTGTAGTTGTTCTATTTTAATCAAATGTTAGCGTGATTTATATTTTTTTTCGCCTCG
ACATCATCTGCCCAGATGCGAAGTTAAGTGCGCAGAAAGTAATATCATGCGTCAATCGTATGTGAATGCTGGTCGCT
ATACTGCTGTCGATTCGATACTAACGCCGCCATCCAGTGTCGAAAACGAGCTCCATTAGTGAGTAACTCTGTGATAT

CTCTCTATAATTAGCAGTTTTTCACTGAAATTCCAGGAAAGGTAATAAACTCAGATTTTTTTTATACTATTGGCTG
CTTGTTACTTATATATCTTGAACTTCTCCCAGCGGGTCTTCAAATACATTTGGGCGATGTTCATGTTCATTAGGCAG
GTATTTCGACATTGAGTCACACGCGAAAAACCGCCGGAATTTTTATGTAATTGCAAGTGGAATTCCGCTGGCAAAA
CTATTGGGCCCGTTAACCTGCATTAATGAATCGGCCAACGCGCGGGGAGAGGCGGTTTGCGTATTGGGCGCTCTTCC
GCTTCCTCGCTCACTGACTCGCTGCGCTCGGTCGTTCGGCTGCGGCGAGCGGTATCAGCTCACTCAAAGGCGGTAAT
ACGGTTATCCACAGAATCAGGGGATAACGCAGGAAAGAACATGTGAGCAAAAGGCCAGCAAAAGGCCAGGAACCGTA
AAAAGGCCGCGTTGCTGGCGTTTTTCCATAGGCTCCGCCCCCCTGACGAGCATCACAAAAATCGACGCTCAAGTCAG
AGGTGGCGAAACCCGACAGGACTATAAAGATACCAGGCGTTTCCCCCTGGAAGCTCCCTCGTGCGCTCTCCTGTTCC
GACCCTGCCGCTTACCGGATACCTGTCCGCCTTTCTCCCTTCGGGAAGCGTGGCGCTTTCTCAATGCTCACGCTGTA
GGTATCTCAGTTCGGTGTAGGTCGTTCGCTCCAAGCTGGGCTGTGTGCACGAACCCCCCGTTCAGCCCGACCGCTGC
GCCTTATCCGGTAACTATCGTCTTGAGTCCAACCCGGTAAGACACGACTTATCGCCACTGGCAGCAGCCACTGGTAA
CAGGATTAGCAGAGCGAGGTATGTAGGCGGTGCTACAGAGTTCTTGAAGTGGTGGCCTAACTACGGCTACACTAGAA
GGACAGTATTTGGTATCTGCGCTCTGCTGAAGCCAGTTACCTTCGGAAAAAGAGTTGGTAGCTCTTGATCCGGCAAA
CAAACCACCGCTGGTAGCGGTGGTTTTTTTGTTTGCAAGCAGCAGATTACGCGCAGAAAAAAAGGATCTCAAGAAGA
TCCTTTGATCTTTTCTACGGGGTCTGACGCTCAGTGGAACGAAAACTCACGTTAAGGGATTTTGGTCATGAGATTAT
CAAAAAGGATCTTCACCTAGATCCTTTTAAATTAAAAATGAAGTTTTAAATCAATCTAAAGTATATATGAGTAAACT
TGGTCTGACAGTTACCAATGCTTAATCAGTGAGGCACCTATCTCAGCGATCTGTCTATTTCGTTCATCCATAGTTGC
CTGACTCCCCGTCGTGTAGATAACTACGATACGGGAGGGCTTACCATCTGGCCCCAGTGCTGCAATGATACCGCGAG
ACCCACGCTCACCGGCTCCAGATTTATCAGCAATAAACCAGCCAGCCGGAAGGGCCGAGCGCAGAAGTGGTCCTGCA
ACTTTATCCGCCTCCATCCAGTCTATTAATTGTTGCCGGGAAGCTAGAGTAAGTAGTTCGCCAGTTAATAGTTTGCG
CAACGTTGTTGCCATTGCTACAGGCATCGTGGTGTCACGCTCGTCGTTTGGTATGGCTTCATTCAGCTCCGGTTCCC
AACGATCAAGGCGAGTTACATGATCCCCCATGTTGTGCAAAAAAGCGGTTAGCTCCTTCGGTCCTCCGATCGTTGTC
AGAAGTAAGTTGGCCGCAGTGTTATCACTCATGGTTATGGCAGCACTGCATAATTCTCTTACTGTCATGCCATCCGT
AAGATGCTTTTCTGTGACTGGTGAGTACTCAACCAAGTCATTCTGAGAATAGTGTATGCGGCGACCGAGTTGCTCTT
GCCCGGCGTCAATACGGGATAATACCGCGCCACATAGCAGAACTTTAAAAGTGCTCATCATTGGAAAACGTTCTTCG
GGGCGAAAACTCTCAAGGATCTTACCGCTGTTGAGATCCAGTTCGATGTAACCCACTCGTGCACCCAACTGATCTTC
AGCATCTTTTACTTTCACCAGCGTTTCTGGGTGAGCAAAAACAGGAAGGCAAAATGCCGCAAAAAAGGGAATAAGGG
CGACACGGAAATGTTGAATACTCATACTCTTCCTTTTTCAATATTATTGAAGCATTTATCAGGGTTATTGTCTCATG
AGCGGATACATATTTGAATGTATTTAGAAAAATAAACAAATAGGGGTTCCGCGCACATTTCCCCGAAAAGTGCCACC
TGACGTCTAAGAAACCATTATTATCATGACATTAACCTATAAAAATAGGCGTATCACGAGGCCCTTTCGTCTCGCGC
GTTTCGGTGATGACGGTGAAAACCTCTGACACATGCAGCTCCCGGAGACGGTCACAGCTTGTCTGTAAGCGGATGCC
GGGAGCAGACAAGCCCGTCAGGGCGCGTCAGCGGGTGTTGGCGGGTGTCGGGGCTGGCTTAACTATGCGGCATCAGA
GCAGATTGTACTGAGAGTGCACCATATGGACATATTGTCGTTAGAACGCGGCTACAATTAATACATAACCTTATGTA
TCATACACATACGATTTAGGTGACACTATA

pBAR3-L1 (Published in Levy et al., 2015)

Key:
NNNN = Gal promoter
NNNN = Truncated Cre sequence
NNNN = CYC1 Terminator
NNNN = *HygMX*
NNNN = 5' iSeq Barcode
NNNN = 5' half of the *URA3* gene
NNNN = lox71
NNNN = Artificial intron start

GAACGCGGCCGCCAGCTGAAGCTTCGTACGCTGCAGGTCGACGGATCCCCGGGTTAATTAAGGGCCCACAAGGTTTT
GCATTGAGGATAGTATAGAAGCAAGAATCATTGAATTACAGGAAAAAAAGGCAAATATGATTCATGCTACAATAAAC
CAAGATGAAGCTGCCATTAGCAGACTAACGCCAGCTGATTTACAGTTCTTATTCAATAACTAATATTTTATTCTCTT
ATTATATATTATTCTCGGAGTTTTTAAGTGACATCACCCGAAAAGAAGCTAAGTCTTTCTCCTAATTCATATTTAAT
TATTGTACATGGACATATCATACGTAATGCTCAACCTTAGCTAGCtagtacggattagaagccgccgagcgggtgac
agccctccgaaggaagactctcctccgtgcgtcctcgtcttcaccggtcgcgttcctgaaacgcagatgtgcctcgc
gccgcactgctccgaacaataaagattctacaatactagcttttatggttatgaagaggaaaaattggcagtaacct
ggccccacaaaccttcaaatgaacgaatcaaattaacaaccataggatgataatgcgattagttttttagccttatt
tctggggtaattaatcagcgaagcgatgatttttgatctattaacagatatataaatgcaaaaactgcataaccact
ttaactaatactttcaacattttcggtttgtattacttcttattcaaatgtaataaaagtatcaacaaaaaattgtt
aatatacctctatactttaacgtcaaggagaaaaaaccccggattctagaactagtggatcccccgggctgcaggaa
ttcgatatcaagcttatcgattgatttacggcgctaaggatgactctggtcagagatacctggcctggtctggacac

agtgcccgtgtcggagccgcgcgagatatggcccgcgctggagtttcaataccggagatcatgcaagctggtggctg
gaccaatgtaaatattgtcatgaactatatccgtaAcctggatagtgaaacaggggcaatggtgcgcctgctggaag
atggcgattagccattaacgcgtaaatgattgctataattatttgatatttatggtgacatatgagaaaggatttca
acatcgacggaaaatatgtagtgctgtctgtaagcactaatattcagtcgccagccgtcattgtcactgtaaagctg
agcgatagaatgcctgatattgactcaatatccgttgcgtttcctgtcaaaagtatgcgtagtgctgaacatttcgt
gatgaatgccaccgaggaagaagcacggcgcggttttgctaaagtgatgtctgagtttggcgaactcttgggtaagg
ttggaattgtcgacctcgagtcatgtaattagttatgtcacgcttacattcacgccctccccccacatccgctctaa
ccgaaaaggaaggagttagacaacctgaagtctaggtccctatttattttttttatagttatgttagtattaagaacg
ttatttatatttcaaatttttcttttttttctgtacagacgcgtgtacgcatgtaacattatactgaaaaccttgct
tgagaaggttttgggacgctcgaaggctttaatttgcggccGGCGCGCCTACCGTTCGTATAGCATACATTATACGA
AGTTATNNNNNAANNNNNTTNNNNNTTNNNNNGGTACCGATATCGGATCCGTCGACAAAAGCCTCCTTTAGTCCATA
TTAACAtacatccacatgtgtttttagtaaacaaattttgggacctaatgcttcaactaactccagtaattccttgg
tggtacgaacatccaatgaagcacacaagtttgtttgcttttcgtgcatgatattaaatagcttggcagcaacagga
ctaggatgagtagcagcacgttccttatatgtagctttcgacatgatttatcttcgtttcctgcaggtttttgttct
gtgcagttgggttaagaatactgggcaatttcatgtttcttcaacactacatatgcgtatatataccaatctaagtc
tgtgctccttccttcgttcttccttCTGTTCGGAGATTACCGAATCGGTCACCCGGCCAGCGACATGGAGGCCCAGA
ATACCCTCCTTGACAGTCTTGACGTGCGCAGCTCAGGGGCATGATGTGACTGTCGCCCGTACATTTAGCCCATACAT
CCCCATGTATAATCATTTGCATCCATACATTTTGATGGCCGCACGGCGCGAAGCAAAAATTACGGCTCCTCGCTGCA
GACCTGCGAGCAGGGAAACGCTCCCCTCACAGACGCGTTGAATTGTCCCCACGCCGCGCCCCTGTAGAGAAATATAA
AAGGTTAGGATTTGCCACTGAGGTTCTTCTTTCATATACTTCCTTTTAAAATCTTGCTAGGATACAGTTCTCACATC
ACATCCGAACATAAACAACCATGGGTAAAAAGCCTGAACTCACCGCGACGTCTGTCGAGAAGTTTCTGATCGAAAAG
TTCGACAGCGTCTCCGACCTGATGCAGCTCTCGGAGGGCGAAGAATCTCGTGCTTTCAGCTTCGATGTAGGAGGGCG
TGGATATGTCCTGCGGGTAAATAGCTGCGCCGATGGTTTCTACAAAGATCGTTATGTTTATCGGCACTTTGCATCGG
CCGCGCTCCCGATTCCGGAAGTGCTTGACATTGGGGAATTCAGCGAGAGCCTGACCTATTGCATCTCCCGCCGTGCA
CAGGGTGTCACGTTGCAAGACCTGCCTGAAACCGAACTGCCCGCTGTTCTGCAGCCGGTCGCGGAGGCCATGGATGC
GATCGCTGCGGCCGATCTTAGCCAGACGAGCGGGTTCGGCCCATTCGGACCGCAAGGAATCGGTCAATACACTACAT
GGCGTGATTTCATATGCGCGATTGCTGATCCCCATGTGTATCACTGGCAAACTGTGATGGACGACACCGTCAGTGCG
TCCGTCGCGCAGGCTCTCGATGAGCTGATGCTTTGGGCCGAGGACTGCCCCGAAGTCCGGCACCTCGTGCACGCGGA
TTTCGGCTCCAACAATGTCCTGACGGACAATGGCCGCATAACAGCGGTCATTGACTGGAGCGAGGCGATGTTCGGGG
ATTCCCAATACGAGGTCGCCAACATCTTCTTCTGGAGGCCGTGGTTGGCTTGTATGGAGCAGCAGACGCGCTACTTC
GAGCGGAGGCATCCGGAGCTTGCAGGATCGCCGCGGCTCCGGGCGTATATGCTCCGCATTGGTCTTGACCAACTCTA
TCAGAGCTTGGTTGACGGCAATTTCGATGATGCAGCTTGGGCGCAGGGTCGATGCGACGCAATCGTCCGATCCGGAG
CCGGGACTGTCGGGCGTACACAAATCGCCCGCAGAAGCGCGGCCGTCTGGACCGATGGCTGTGTAGAAGTACTCGCC
GATAGTGGAAACCGACGCCCCAGCACTCGTCCGAGGGCAAAGGAATAATCAGTACTGACAATAAAAAGATTCTTGTT
TTCAAGAACTTGTCATTTGTATAGTTTTTTTATATTGTAGTTGTTCTATTTTAATCAAATGTTAGCGTGATTTATAT
TTTTTTTCGCCTCGACATCATCTGCCCAGATGCGAAGTTAAGTGCGCAGAAAGTAATATCATGCGTCAATCGTATGT
GAATGCTGGTCGCTATACTGCTGTCGATTCGATACTAACGCCGCCATCCAGTGTCGAAAACGAGCTCCATTAGTGAG
TAACTCTGTGATATCTCTCTATAATTAGCAGTTTTTCACTGAAATTCCAGGAAAGGTAATAAACTCAGATTTTTTTT
TATACTATTGGCTGCTTGTTACTTATATATCTTGAACTTCTCCCAGCGGGTCTTCAAATACATTTGGGCGATGTTCA
TGTTCATTAGGCAGGTATTTCGACATTGAGTCACACGCGAAAAACCGCCGGAATTTTTTATGTAATTGCAAGTGGAA
TTCCGCTGGCAAAACTATTGGGCCCGTTAACCTGCATTAATGAATCGGCCAACGCGCGGGGAGAGGCGGTTTGCGTA
TTGGGCGCTCTTCCGCTTCCTCGCTCACTGACTCGCTGCGCTCGGTCGTTCGGCTGCGGCGAGCGGTATCAGCTCAC
TCAAAGGCGGTAATACGGTTATCCACAGAATCAGGGGATAACGCAGGAAAGAACATGTGAGCAAAAGGCCAGCAAAA
GGCCAGGAACCGTAAAAAGGCCGCGTTGCTGGCGTTTTTCCATAGGCTCCGCCCCCCTGACGAGCATCACAAAAATC
GACGCTCAAGTCAGAGGTGGCGAAACCCGACAGGACTATAAAGATACCAGGCGTTTCCCCCTGGAAGCTCCCTCGTG
CGCTCTCCTGTTCCGACCCTGCCGCTTACCGGATACCTGTCCGCCTTTCTCCCTTCGGGAAGCGTGGCGCTTTCTCA
ATGCTCACGCTGTAGGTATCTCAGTTCGGTGTAGGTCGTTCGCTCCAAGCTGGGCTGTGTGCACGAACCCCCCGTTC
AGCCCGACCGCTGCGCCTTATCCGGTAACTATCGTCTTGAGTCCAACCCGGTAAGACACGACTTATCGCCACTGGCA
GCAGCCACTGGTAACAGGATTAGCAGAGCGAGGTATGTAGGCGGTGCTACAGAGTTCTTGAAGTGGTGGCCTAACTA
CGGCTACACTAGAAGGACAGTATTTGGTATCTGCGCTCTGCTGAAGCCAGTTACCTTCGGAAAAAGAGTTGGTAGCT
CTTGATCCGGCAAACAAACCACCGCTGGTAGCGGTGGTTTTTTTGTTTGCAAGCAGCAGATTACGCGCAGAAAAAAA
GGATCTCAAGAAGATCCTTTGATCTTTTCTACGGGGTCTGACGCTCAGTGGAACGAAAACTCACGTTAAGGATTTT
GGTCATGAGATTATCAAAAAGGATCTTCACCTAGATCCTTTTAAATTAAAAATGAAGTTTTAAATCAATCTAAAGTA
TATATGAGTAAACTTGGTCTGACAGTTACCAATGCTTAATCAGTGAGGCACCTATCTCAGCGATCTGTCTATTTCGT
TCATCCATAGTTGCCTGACTCCCCGTCGTGTAGATAACTACGATACGGGAGGGCTTACCATCTGGCCCCAGTGCTGC
AATGATACCGCGAGACCCACGCTCACCGGCTCCAGATTTATCAGCAATAAACCAGCCAGCCGGAAGGGCCGAGCGCA
GAAGTGGTCCTGCAACTTTATCCGCCTCCATCCAGTCTATTAATTGTTGCCGGGAAGCTAGAGTAAGTAGTTCGCCA
GTTAATAGTTTGCGCAACGTTGTTGCCATTGCTACAGGCATCGTGGTGTCACGCTCGTCGTTTGGTATGGCTTCATT

CAGCTCCGGTTCCCAACGATCAAGGCGAGTTACATGATCCCCCATGTTGTGCAAAAAAGCGGTTAGCTCCTTCGGTC
CTCCGATCGTTGTCAGAAGTAAGTTGGCCGCAGTGTTATCACTCATGGTTATGGCAGCACTGCATAATTCTCTTACT
GTCATGCCATCCGTAAGATGCTTTTCTGTGACTGGTGAGTACTCAACCAAGTCATTCTGAGAATAGTGTATGCGGCG
ACCGAGTTGCTCTTGCCCGGCGTCAATACGGGATAATACCGCGCCACATAGCAGAACTTTAAAAGTGCTCATCATTG
GAAAACGTTCTTCGGGGCGAAAACTCTCAAGGATCTTACCGCTGTTGAGATCCAGTTCGATGTAACCCACTCGTGCA
CCCAACTGATCTTCAGCATCTTTTACTTTCACCAGCGTTTCTGGGTGAGCAAAAACAGGAAGGCAAAATGCCGCAAA
AAAGGGAATAAGGGCGACACGGAAATGTTGAATACTCATACTCTTCCTTTTTCAATATTATTGAAGCATTTATCAGG
GTTATTGTCTCATGAGCGGATACATATTTGAATGTATTTAGAAAAATAAACAAATAGGGGTTCCGCGCACATTTCCC
CGAAAAGTGCCACCTGACGTCTAAGAAACCATTATTATCATGACATTAACCTATAAAAATAGGCGTATCACGAGGCC
CTTTCGTCTCGCGCGTTTCGGTGATGACGGTGAAAACCTCTGACACATGCAGCTCCCGGAGACGGTCACAGCTTGTC
TGTAAGCGGATGCCGGGAGCAGACAAGCCCGTCAGGGCGCGTCAGCGGGTGTTGGCGGGTGTCGGGGCTGGCTTAAC
TATGCGGCATCAGAGCAGATTGTACTGAGAGTGCACCATATGGACATATTGTCGTTAGAACGCGGCTACAATTAATA
CATAACCTTATGTATCATACACATACGATTTAGGTGACACTATA

L001

Key:
NNNN = Gal promoter
NNNN = Truncated Cre sequence
NNNN = CYC1 Terminator
NNNN = *HygMX*
NNNN = 3' iSeq Barcode
NNNN = 3' half of the *URA3* gene
NNNN = lox66
NNNN = Artificial intron end

GAACGCGGCCGCCAGCTGAAGCTTCGTACGCTGCAGGTCGACGGATCCCCGGGTTAATTAAGGGCCCAATAGTTTTG
CCAGCGGAATTCCACTTGCAATTACATAAAAAATTCCGGCGGTTTTTCGCGTGTGACTCAATGTCGAAATACCTGCC
TAATGAACATGAACATCGCCCAAATGTATTTGAAGACCCGCTGGGAGAAGTTCAAGATATATAAGTAACAAGCAGCC
AATAGTATAAAAAAAAATCTGAGTTTATTACCTTTCCTGGAATTTCAGTGAAAAACTGCTAATTATAGAGAGATATC
ACAGAGTTACTCACTAATGGCTAGCtagtacggattagaagccgccgagcgggtgacagccctccgaaggaagactc
tcctccgtgcgtcctcgtcttcaccggtcgcgttcctgaaacgcagatgtgcctcgcgccgcactgctccgaacaat
aaagattctacaatactagcttttatggttatgaagaggaaaaattggcagtaacctggccccacaaaccttcaaat
gaacgaatcaaattaacaaccataggatgataatgcgattagttttttagccttatttctggggtaattaatcagcg
aagcgatgatttttgatctattaacagatatataaatgcaaaaactgcataaccactttaactaatactttcaacat
tttcggtttgtattacttcttattcaaatgtaataaaagtatcaacaaaaaattgttaatatacctctatactttaa
cgtcaaggagaaaaaaccccggattctagaactagtggatcccccgggctgcaggaattcgatatcaagcttatcga
ttgatttacggcgctaaggatgactctggtcagagatacctggcctggtctggacacagtgcccgtgtcggagccgc
gcgagatatggcccgcgctggagtttcaataccggagatcatgcaagctggtggctggaccaatgtaaatattgtca
tgaactatatccgtaAcctggatagtgaaacaggggcaatggtgcgcctgctggaagatggcgattagccattaacg
cgtaaatgattgctataattatttgatatttatggtgacatatgagaaaggatttcaacatcgacggaaaatatgta
gtgctgtctgtaagcactaatattcagtcgccagccgtcattgtcactgtaaagctgagcgatagaatgcctgatat
tgactcaatatccgttgcgtttcctgtcaaaagtatgcgtagtgctgaacatttcgtgatgaatgccaccgaggaag
aagcacggcgcggttttgctaaagtgatgtctgagtttggcgaactcttgggtaaggttggaattgtcgacctcgag
tcatgtaattagttatgtcacgcttacattcacgccctccccccacatccgctctaaccgaaaaggaaggagttaga
caacctgaagtctaggtccctatttatttttttatagttatgttagtattaagaacgttatttatatttcaaatttt
tctttttttttctgtacagacgcgtgtacgcatgtaacattatactgaaaaccttgcttgagaaggttttgggacgct
cgaaggctttaatttgcggccGGCGCGCCTACCGTTCGTATAATGTATGCTATACGAAGTTATNNNNNAANNNNNAA
NNNNNTTNNNNNGGTACCGATATCAGATCTAAGCTTGAATTCGAATTTTTACTAACAAATGGTATTATTTATAAcag
atcttgactgattttttccatggagggcacagttaagccgctaaaggcattatccgccaagtacaatttttttactctt
cgaagacagaaaatttgctgacattggtaatacagtcaaattgcagtactctgcgggtgtatacagaatagcagaat
gggcagacattacgaatgcacacggtgtggtgggcccaggtattgttagcggtttgaagcaggcggcagaagaagta
acaaaggaacctagaggccttttgatgttagcagaattgtcatgcaagggctccctatctactggagaatatactaa
gggtactgttgacattgcgaagagcgacaaagatttttgttatcggctttattgctcaaagagacatgggtggaagag
atgaaggttacgattggttgattatgacacccggtgtgggtttagatgacaagggagacgcattgggtcaacagtat
agaaccgtggatgatgtggtctctacaggatctgacattattattgttggaagaggactatttgcaaagggaaggga
tgctaaggtagagggtgaacgttacagaaaagcaggctgggaagcatatttgagaagatgcggccagcaaaactaaa
aaactgtattataagtaaatgcatgtatactaaactcacaaattagagcttcaatttaattatatcagttattaccG

GTCACCCGGCCAGCGACATGGAGGCCCAGAATACCCTCCTTGACAGTCTTGACGTGCGCAGCTCAGGGGCATGATGT
GACTGTCGCCCGTACATTTAGCCCATACATCCCCATGTATAATCATTTGCATCCATACATTTTGATGGCCGCACGGC
GCGAAGCAAAAATTACGGCTCCTCGCTGCAGACCTGCGAGCAGGGAAACGCTCCCCTCACAGACGCGTTGAATTGTC
CCCACGCCGCGCCCCTGTAGAGAAATATAAAAGGTTAGGATTTGCCACTGAGGTTCTTCTTTCATATACTTCCTTTT
AAAATCTTGCTAGGATACAGTTCTCACATCACATCCGAACATAAACAACCATGGGTAAAAAGCCTGAACTCACCGCG
ACGTCTGTCGAGAAGTTTCTGATCGAAAAGTTCGACAGCGTCTCCGACCTGATGCAGCTCTCGGAGGGCGAAGAATC
TCGTGCTTTCAGCTTCGATGTAGGAGGGCGTGGATATGTCCTGCGGGTAAATAGCTGCGCCGATGGTTTCTACAAAG
ATCGTTATGTTTATCGGCACTTTGCATCGGCCGCGCTCCCGATTCCGGAAGTGCTTGACATTGGGGAATTCAGCGAG
AGCCTGACCTATTGCATCTCCCGCCGTGCACAGGGTGTCACGTTGCAAGACCTGCCTGAAACCGAACTGCCCGCTGT
TCTGCAGCCGGTCGCGGAGGCCATGGATGCGATCGCTGCGGCCGATCTTAGCCAGACGAGCGGGTTCGGCCCATTCG
GACCGCAAGGAATCGGTCAATACACTACATGGCGTGATTTCATATGCGCGATTGCTGATCCCCATGTGTATCACTGG
CAAACTGTGATGGACGACACCGTCAGTGCGTCCGTCGCGCAGGCTCTCGATGAGCTGATGCTTTGGGCCGAGGACTG
CCCCGAAGTCCGGCACCTCGTGCACGCGGATTTCGGCTCCAACAATGTCCTGACGGACAATGGCCGCATAACAGCGG
TCATTGACTGGAGCGAGGCGATGTTCGGGGATTCCCAATACGAGGTCGCCAACATCTTCTTCTGGAGGCCGTGGTTG
GCTTGTATGGAGCAGCAGACGCGCTACTTCGAGCGGAGGCATCCGGAGCTTGCAGGATCGCCGCGGCTCCGGGCGTA
TATGCTCCGCATTGGTCTTGACCAACTCTATCAGAGCTTGGTTGACGGCAATTTCGATGATGCAGCTTGGGCGCAGG
GTCGATGCGACGCAATCGTCCGATCCGGAGCCGGGACTGTCGGGCGTACACAAATCGCCCGCAGAAGCGCGGCCGTC
TGGACCGATGGCTGTGTAGAAGTACTCGCCGATAGTGGAAACCGACGCCCCAGCACTCGTCCGAGGGCAAAGGAATA
ATCAGTACTGACAATAAAAAGATTCTTGTTTTCAAGAACTTGTCATTTGTATAGTTTTTTTATATTGTAGTTGTTCT
ATTTTAATCAAATGTTAGCGTGATTTATATTTTTTTTCGCCTCGACATCATCTGCCCAGATGCGAAGTTAAGTGCGC
AGAAAGTAATATCATGCGTCAATCGTATGTGAATGCTGGTCGCTATACTGCTGTCGATTCGATACTAACGCCGCCAT
CCAGTGTCGAAAACGAGCTCTAAGGTTGAGCATTACGTATGATATGTCCATGTACAATAATTAAATATGAATTAGGA
GAAAGACTTAGCTTCTTTTCGGGTGATGTCACTTAAAAACTCCGAGAATAATATATAATAAGAGAATAAAATATTAG
TTATTGAATAAGAACTGTAAATCAGCTGGCGTTAGTCTGCTAATGGCAGCTTCATCTTGGTTTATTGTAGCATGAAT
CATATTTGCCTTTTTTTCCTGTAATTCAATGATTCTTGCTTCTATACTATCCTCAATGCAAAACCTTGTGGGCCCGT
TAACCTGCATTAATGAATCGGCCAACGCGCGGGGAGAGGCGGTTTGCGTATTGGGCGCTCTTCCGCTTCCTCGCTCA
CTGACTCGCTGCGCTCGGTCGTTCGGCTGCGGCGAGCGGTATCAGCTCACTCAAAGGCGGTAATACGGTTATCCACA
GAATCAGGGGATAACGCAGGAAAGAACATGTGAGCAAAAGGCCAGCAAAAGGCCAGGAACCGTAAAAAGGCCGCGTT
GCTGGCGTTTTTCCATAGGCTCCGCCCCCCTGACGAGCATCACAAAAATCGACGCTCAAGTCAGAGGTGGCGAAACC
CGACAGGACTATAAAGATACCAGGCGTTTCCCCCTGGAAGCTCCCTCGTGCGCTCTCCTGTTCCGACCCTGCCGCTT
ACCGGATACCTGTCCGCCTTTCTCCCTTCGGGAAGCGTGGCGCTTTCTCAATGCTCACGCTGTAGGTATCTCAGTTC
GGTGTAGGTCGTTCGCTCCAAGCTGGGCTGTGTGCACGAACCCCCCGTTCAGCCCGACCGCTGCGCCTTATCCGGTA
ACTATCGTCTTGAGTCCAACCCGGTAAGACACGACTTATCGCCACTGGCAGCAGCCACTGGTAACAGGATTAGCAGA
GCGAGGTATGTAGGCGGTGCTACAGAGTTCTTGAAGTGGTGGCCTAACTACGGCTACACTAGAAGGACAGTATTTGG
TATCTGCGCTCTGCTGAAGCCAGTTACCTTCGGAAAAAGAGTTGGTAGCTCTTGATCCGGCAAACAAACCACCGCTG
GTAGCGGTGGTTTTTTTGTTTGCAAGCAGCAGATTACGCGCAGAAAAAAAGGATCTCAAGAAGATCCTTTGATCTTT
TCTACGGGGTCTGACGCTCAGTGGAACGAAAACTCACGTTAAGGGATTTTGGTCATGAGATTATCAAAAAGGATCTT
CACCTAGATCCTTTTAAATTAAAAATGAAGTTTTAAATCAATCTAAAGTATATATGAGTAAACTTGGTCTGACAGTT
ACCAATGCTTAATCAGTGAGGCACCTATCTCAGCGATCTGTCTATTTCGTTCATCCATAGTTGCCTGACTCCCCGTC
GTGTAGATAACTACGATACGGGAGGGCTTACCATCTGGCCCCAGTGCTGCAATGATACCGCGAGACCCACGCTCACC
GGCTCCAGATTTATCAGCAATAAACCAGCCAGCCGGAAGGGCCGAGCGCAGAAGTGGTCCTGCAACTTTATCCGCCT
CCATCCAGTCTATTAATTGTTGCCGGGAAGCTAGAGTAAGTAGTTCGCCAGTTAATAGTTTGCGCAACGTTGTTGCC
ATTGCTACAGGCATCGTGGTGTCACGCTCGTCGTTTGGTATGGCTTCATTCAGCTCCGGTTCCCAACGATCAAGGCG
AGTTACATGATCCCCCATGTTGTGCAAAAAAGCGGTTAGCTCCTTCGGTCCTCCGATCGTTGTCAGAAGTAAGTTGG
CCGCAGTGTTATCACTCATGGTTATGGCAGCACTGCATAATTCTCTTACTGTCATGCCATCCGTAAGATGCTTTTCT
GTGACTGGTGAGTACTCAACCAAGTCATTCTGAGAATAGTGTATGCGGCGACCGAGTTGCTCTTGCCCGGCGTCAAT
ACGGGATAATACCGCGCCACATAGCAGAACTTTAAAAGTGCTCATCATTGGAAAACGTTCTTCGGGGCGAAAACTCT
CAAGGATCTTACCGCTGTTGAGATCCAGTTCGATGTAACCCACTCGTGCACCCAACTGATCTTCAGCATCTTTTACT
TTCACCAGCGTTTCTGGGTGAGCAAAAACAGGAAGGCAAAATGCCGCAAAAAAGGGAATAAGGGCGACACGGAAATG
TTGAATACTCATACTCTTCCTTTTTCAATATTATTGAAGCATTTATCAGGGTTATTGTCTCATGAGCGGATACATAT
TTGAATGTATTTAGAAAAATAAACAAATAGGGGTTCCGCGCACATTTCCCCGAAAAGTGCCACCTGACGTCTAAGAA
ACCATTATTATCATGACATTAACCTATAAAAATAGGCGTATCACGAGGCCCTTTCGTCTCGCGCGTTTCGGTGATGA
CGGTGAAAACCTCTGACACATGCAGCTCCCGGAGACGGTCACAGCTTGTCTGTAAGCGGATGCCGGGAGCAGACAAG
CCCGTCAGGGCGCGTCAGCGGGTGTTGGCGGGTGTCGGGGCTGGCTTAACTATGCGGCATCAGAGCAGATTGTACTG
AGAGTGCACCATATGGACATATTGTCGTTAGAACGCGGCTACAATTAATACATAACCTTATGTATCATACACATACG
ATTTAGGTGACACTATA

Supplemental References

1. Gietz, R.D. and Schiestl, R.H. (2007). High-efficiency yeast transformation using the LiAc/SS carrier DNA/PEG method. Nat. Protoc. *2*, 31–34.
2. Lindstrom, D.L. and Gottschling, D.E. (2009). The mother enrichment program: A genetic system for facile replicative life span analysis in Saccharomyces cerevisiae. Genetics. *183*, 413–422.

Detailed Protocol for iSeq

Table of Contents:

Section — Page Numbers
Generating Plasmid iSeq Barcode Libraries…… 2 - 4
Generating Yeast iSeq Barcode Libraries…… 5
Crossing Yeast iSeq Barcode Library Strains with Deletion Strains of Interest…… 6 - 7
Crossing Yeast Strains Carrying Compatible iSeq Single Barcode Constructs…….. 8
Pooled Growth…… 9
Barcode Sequencing and Analysis…….. 10 - 11
Media Recipes…… 12 - 13
Appendix (Primers, Strains, Plasmids, PCR Products, References)…… 14 - 24

Generating Plasmid iSeq Barcode Libraries

3' Plasmid iSeq BC Library:

1. Preparing Insert Containing lox66 site and DNA BC
    a. Perform 4 PCR reactions using the recipe and cycling conditions outlined below:

        Amount of each reagent per tube:
        i. 10 uL 5X PrimeStar HS Buffer (Clontech R010A)
        ii. 2 uL Forward Primer (10uM stock of P7, see Appendix for sequence)
        iii. 2 uL Reverse Primer (10uM stock of P8, see see Appendix for sequence)
        iv. 1 uL template (M207/pBAR1 plasmid DNA-see Appendix for sequence, available upon request)
        v. 0.5 uL PrimeStar HS enzyme (Clontech R010A)
        vi. 4 uL dNTPs
        vii. 30.5 uL H_2O

        Cycling conditions (perform 3 cycles for ii-iv and 27 cycles for v-vii):
        i. 98°C, 10 min
        ii. 98°C, 10 sec
        iii. 60°C, 30 sec
        iv. 72°C, 1 min and 30 sec
        v. 98°C, 10 sec
        vi. 64°C, 30s
        vii. 72°C, 1 min and 30 sec
        viii. 72°C, 10 min
        ix. 4°C, Inf

    b. Use QIAquick PCR Purification Kit (QIAGEN, #28106) to purify the product of each PCR reaction, eluting in 50 uL of H_2O each.
    c. Perform a KpnI (NEB, #R0142S) and XhoI (NEB, #R0146S) digest on each purified rxn:
        i. Add a mix of 29 uL H_2O, 10 uL NEB Buffer 1, 1 uL BSA, 5 uL KpnI and 5 uL XhoI to each tube containing 50 uL purified PCR product from step (c).
        ii. Incubate at 37°C for 2 hr.
        iii. Run digested samples on 1.2% agarose gel and purify with Qiaquick Gel Extraction Kit (QIAGEN, #28706).

2. Restriction Enzyme Digest and Column Purification of Vector Containing the 3' half of the *URA3* gene and *HygMX* (M272-see Appendix for sequence, available upon request)
    a. Digest vector DNA with XhoI (NEB, #R0146S) (37°C for 2 hr, then 65°C for 20 min).
    b. Column purify with QIAquick PCR Purification Kit (QIAGEN, #28106).
    c. Digest product from (b) with KpnI (NEB, #R0142S) (37°C for 2 hr, then 65°C for 20 min).
    d. Column purify with QIAquick PCR Purification Kit (QIAGEN, #28106).

3. Alkaline Phosphatase Digestion and Gel Purification of Vector
    a. Add together 5 ug DNA from Step #2 + 5 uL alkaline phosphatase (Roche Diagnostics, #10713023001) + 5 uL 10X AP buffer + H_2O to 50 uL.
    b. Incubate 37°C for 30 min.
    c. Add an additional 5 uL enzyme and incubate another 30 min.
    d. Add small volume of concentrated EDTA such that the final concentration is 5 mM and incubate at 75°C for 10 min.
    e. Run digested samples on 1.1% agarose gel and purify with Qiaquick Gel Extraction Kit (QIAGEN, #28706).

4. Ligation of Vector and Insert
    a. Add the following reagents to each of 10 tubes:
        i. 1 uL T4 DNA ligase (NEB, #M0202S)
        ii. 2 uL 10X ligase buffer
        iii. 10 ng purified insert (from Step #1)
        iv. 50 ng purified vector (from Step #3)
        v. H_2O up to 20 uL
    b. For vector only control, prepare 10 additional tubes as above omitting the insert DNA in (iii).
    c. Incubate 16°C overnight.
    d. Purify all 10 vector+insert ligation reactions using 1 QIAquick PCR Purification Kit column (QIAGEN, #28106), and all 10 vector-only control ligation using a second column.

5. Electroporation and Plating
    a. Prepare 40 LB+Amp plates (more can be required if generating larger libraries).
    b. Thaw (or cool) the following on ice:
        i. ElectroMAX DH10B Cells, 1 tube of 0.5mL (Invitrogen, 18290-015)
        ii. pUC control DNA (Invitrogen, 18290-015)
        iii. Purified vector+insert ligation DNA (from Step #4)
        iv. Purified vector only control ligation DNA (from Step #4)
        v. 3 eppendorf tubes, 1.7 mL, labeled for each of the following electroporations:
            1. Sample 1: vector+insert (to generate larger BC libraries, include multiple tubes, and perform electroporations for each one, using this DNA sample)
            2. Sample 2: vector-only control
            3. Sample 3: pUC control
        vi. 3 cuvettes (Bio-Rad, #1652086) (one is required for each sample in [v])
    c. Warm S.O.C. media (Invitrogen, 18290-015) to 37°C.
    d. Pipet 50 uL thawed cells to each labeled eppendorf tube.
    e. Turn on electroporator (i.e. the Gene Pulser Xcell, Bio-Rad, #1652660) to the following settings:
        i. 200 Ohms
        ii. 25 uFD
        iii. 2.5 Volts
        iv. Set monitor to time constant
    f. Sequentially, pipet 5 uL corresponding DNA to each labeled eppendorf tube containing cells, and then transfer the mixture to a cuvette and electroporate.
    g. After each electroporation, immediately add 1 mL warmed S.O.C. to cuvette and transfer cells to 5 mL plastic test tube (Falcon, #352063).
    h. After all electroporations are finished, incubate the test tubes for 1 hr, shaking at 37°C.
    i. Vector+insert sample: Add 3.8 mL LB+Amp to the test tube with the vector+insert sample and mix well. To generate 1:20 dilution for estimating colony numbers, dilute 50 uL of this mixture in 950 uL LB+Amp, and plate 200 uL to each of 3 LB+Amp plates using sterile glass beads. For remaining, undiluted, mixture plate 200 uL to each of 20 LB+Amp plates using glass beads.
    j. pUC control: dilute 10 uL cells electroporated with pUC control DNA to 990 uL S.O.C. and plate 50 uL of this 1:100 dilution to 1 LB+Amp plate for determining electroporation efficiency
    k. Vector only control: Add 3.8 mL LB+Amp to test tube with the vector only control sample and mix well. Plate 200 ul of this mixture to each of 3+LB Amp plates.
    l. Grow plates at 37°C overnight.

6. Scraping Plates, Pooling Colonies, and Preparing Plasmid BC Library DNA
    a. To estimate the number of unique BCs in the generated library first use the 3 plates of 1:20 dilutions of vector+insert electroporation to estimate the total number of colonies on the higher density platings (i.e. if there were and average of 100 colonies on the 1:20 dilution plates, and there were 20 high density plates, the total number of colonies on these high density plates would be 100 x 20 x 20 plates = 40,000 colonies total). Next, use the number of colonies observed on the vector-only control platings to estimate how many of the total colonies contain a plasmid in which the vector self-ligated such that no BC or lox site is present (i.e. if the average number of colonies on the vector-only control plates was 50, the total number of colonies on the experimental plates

containing plasmids without barcodes would be 50 colonies x 20 plates = 1,000). Finally, subtract the estimated number of colonies containing no BCs from the total number of colonies to get an estimate of the number of barcodes in the pool (i.e. for the examples given this would be 40,000 total colonies – 1,000 colonies without a BC = 39,000 barcodes total).

b. Add 5 mL LB+Amp to each of the densely plated vector+insert samples plates and mix colonies into solution with sterile scraper.
c. Pool liquid from each plate into sterile 500 mL flask.
d. Shake at 37°C for 1 hr to mix solution well.
e. Store aliquots of this plasmid library in a final concentration of 17% glycerol in -80°C for future plasmid library DNA preps.
f. With remaining cells, purify plasmid library DNA with QIAGEN Plasmid Maxi Kit (QIAGEN, #12162).

5' Plasmid iSeq BC Library:

The protocol for generating the 5' Plasmid BC library was published in Levy et al., 2015. The process is essentially the same as described above in Steps #1-6, except primers P7 and P9 (see Appendix for sequences) are used in the PCR to generated the insert in Step #1, and the vector backbone used in Step #2 is M264/pBAR3, and contains *HygMX*, the 5' half of the *URA3* gene (see Appendix for sequence, plasmid available upon request).

Generating Yeast iSeq Barcode Libraries

5' Yeast iSeq Barcode Library:

1. Obtaining DNA for Yeast Transformation using a PCR from Plasmid Barcode Library
   a. Perform PCR with the recipe and cycling conditions outlined below:

      Amount of each reagent per tube:
      i. 10 uL 5X PrimeStar HS Buffer (Clontech R010A)
      ii. 2 uL Forward Primer (10 uM stock of P22, see Appendix for sequence)
      iii. 2 uL Reverse Primer (10 uM stock of P10, see Appendix for sequence)
      iv. 12 ng 5' Plasmid BC Library
      v. 0.5 uL PrimeStar HS enzyme (Clontech R010A)
      vi. 4 uL dNTPs (Clontech R010A)
      vii. up to 50 uL with H_2O

      Cycling conditions (perform 30 cycles for steps ii-v):
      i. 98°C, 2 min
      ii. 98°C, 10 sec
      iii. 55°C, 15 sec
      iv. 72°C, 3 min
      v. 72°C, 10min
      vi. 4°C, Inf

   b. Purify PCR product using QIAquick PCR Purification Kit (QIAGEN, #28106), eluting with 50 uL H_2O.
   c. Take concentration of purified product using Qubit (Thermo Fisher, #Q33216), and run 1 uL on a gel to confirm successful PCR.

2. Transforming Barcode Library into Yeast Starting Strain
   a. Use 1 ug of PCR product to transform the starting yeast strain 'HR28' (genotype in Appendix, strain available upon request) by homologous recombination (Gietz and Schiestl, 2007), and plate to YPD+Hyg (see Media Recipes) plates to select transformants.
   b. Grow at 30°C for 1 day, then replica plate each transformation plate to a new YPD+Hyg plate to avoid lawn formation.
   c. Pick single colonies from transformation plate after an additional 2 days of growth and save picked colonies in 96-well plate in a final concentration of 17% glycerol.
   d. Verify selected colonies for growth on YPD+Hyg and lack of growth on YPD+Nat (see Media Recipes).
   e. Also verify for growth on SC-URA+Gal plates (see Media Recipes) after mating to tester strain 'HR296-1-A1' (genotype in Appendix, strain available upon request).

3' Yeast iSeq Barcode Library:

To generate strains for the 3' yeast barcode library follow the same protocol as the 5' yeast barcode library protocol above with the following modifications:

- For the PCR in Step #1, use the primers P22 and P12 and the 3' Plasmid BC Library as starting DNA.
- For Step #2a, transform into the starting yeast strain 'HR27' (genotype in Appendix, strain available upon request).
- For Step #2e, use tester strain 'MIA31' (genotype in Appendix, strain available upon request), to test for functional barcode construct.

Crossing Yeast iSeq Barcode Library Strains with Deletion Strains of Interest

Obtaining *NatMX* marked gene deletion strains carrying the 3' iSeq BC construct

1. Preparing strains and performing matings:
    a. Streak out each *NatMX* marked gene deletion MATa strain for single colonies on YPD from -80°C glycerol stocks.
    b. Streak out 3 strains from the 3' yeast barcode library from the -80°C glycerol stocks per *NatMX* marked deletion strain streaked above. (Note: more BC strains can be used if more BC replicate strains are desired).
    c. To mate, overlay 3 unique iSeq barcode strains with each *NatMX* marked gene deletion strain on YPD using a sterile toothpick. Grow at room temperature overnight.

2. Selecting and sporulating diploids:
    a. Replica mating plates to YPD+Nat+Hyg (see Media Recipes), grow overnight at 30°C to select for diploids.
    b. Transfer selected diploids from each successful mating to 5 mL YPD using sterile wooden stick and grow rotating overnight at 30°C.
    c. If desired, save each diploid strain in 17% glycerol in 96-well plate in -80°C.
    d. Transfer 250 uL overnight culture to 1.7 mL eppie, spin, and wash with 500 uL sterile H_2O.
    e. Re-suspend in 2 mL Supplemented Sporulation Media (see Media Recipes) and place spinning at 25°C for 5 days.

3. Selecting haploids and verifying successful selection
    a. Spin 400 uL sporulated cells down, re-suspend in 20 uL sterile H_2O, pipet to "MATα/Single-Barcode/Single-Deletion Selection Plates" (see Media Recipes) and spread cells over 1/8 of plate using sterile toothpick.
    b. Grow plates at 30°C for 3-4 days and pick single colonies.
    c. Save each selected haploid in 17% glycerol in 96-well plate in -80°C.
    d. For each selected haploid, extract DNA (to use to identify iSeq barcode sequence) using the following steps:
        i. Use sterile pipet tip to transfer cells from yeast colony to 50 uL colony lysis buffer (recipe on next page) in 1.7 mL eppie.
        ii. Incubate at 37°C for 1 hour.
        iii. Incubate at 95°C for 10 min.
        iv. Vortex 15 sec.
        v. Spin at 13,000 rpm for 1 min.
        vi. Use 1 ul of the supernatant per PCR rxn.
    e. If desired, the DNA barcode located at the gene deletion site for each strain can be verified via PCR and Sanger sequencing (see Winzeler et al., 1999 and Smith et al., 2009).
    f. If desired, verify selection of correct mating type and ploidy (suggested for at least a subset of selected strains).

4. Identifying BC sequence
    a. Perform the following PCR to amplify the iSeq BC associated with each *NatMX* marked gene deletion:

        Amount of each reagent per tube:
        i. 12.5 uL OneTaq 2X MM (NEB, #M0482S)
        ii. 0.5 uL Forward Primer (10uM stock of P18, see Appendix for sequence)
        iii. 0.5 uL Reverse Primer (10uM stock of P27, see Appendix for sequence)
        iv. 0.5 uL DNA
        v. 11 uL H_2O

Cycling conditions (perform 30 cycles for steps ii-iv):

i. 94°C, 30 sec
ii. 94°C, 20 sec
iii. 50°C, 30 sec
iv. 68°C, 45 sec
v. 68°C, 5 min
vi. 4°C, Inf

b. Digest excess primers by adding 2 uL ExoSAP IT (Affimetrix, #78200) to 5 uL PCR reaction and incubating at 37°C for 15 min, then 80°C for 15 min.
c. Send for Sanger sequencing using P18 and then align the Sanger sequence to the sequence in Appendix to determine iSeq barcode sequence(s) associated with each gene deletion.

Note: Alternatively, these matings, sporulations, and selections steps could be done on pre-arrayed deletion and barcode strains using automated robotic pinnings.

Obtaining *KanMX* marked gene deletion strains carrying the 5' iSeq BC construct

To generate haploid strains carrying the *KanMX* marked deletion and a 5' iSeq BC construct, follow the same protocol as the *NatMX/*3' iSeq BC construct haploids above, with the following modifications:

- In Step #1, streak out *KanMX* marked deletion strains (should also be of MATa mating type), and 5' yeast barcode library strains.
- In Step #2a use YPD+G418+Hyg in selection plates rather than YPD+Nat+Hyg.
- In Step #3a use "MATa/Single-Barcode/Single-Deletion Selection Plates" (see Media Recipes).
- In Step #4a, use the P19 and P28 primers to amplify the iSeq BC, and P19 in Step #4c.

Recipe for Colony Lysis Buffer:

22.5 uL Tween-20
22.5 uL NP-40
250 uL 1 M KCl
50 uL 1 M Tris-HCl pH 8.3
5 mg Lyticase (Sigma, #L4025-250KU)
→up to 5 mL with sterile H_2O

Crossing Yeast Strains Carrying Compatible iSeq Single Barcode Constructs

1. Preparing strains and performing matings
   a. Streak *KanMX* marked gene deletion strains carrying the 5' iSeq BC construct for single colonies on YPD from frozen stock.
   b. Streak *NatMX* marked gene deletion strains carrying the 3' iSeq BC construct for single colonies on YPD from frozen stock.
   c. Take single colony for each of strain and spread to line on YPD using sterile toothpick and grow at 30°C overnight.
   d. Mate all possible combinations of *KanMX* marked strains by *NatMX* marked strains by cross stamping and growing on YPD overnight at room temperature (Note: these mating steps, as well as subsequent steps, could be automated by using a pinning robot).

2. Selecting diploids and inducing recombination at iSeq barcode locus
   a. Replica mating plates to YPD+Nat+G418 (see Media Recipes) and grow at 30°C overnight.
   b. To induce recombination at iSeq barcode locus, replica plates from previous step to SC+Gal-URA (see Media Recipes), and grow at 30°C for 2 days.
   c. For each strain from previous step, take streak using sterile wooden stick, inoculate 1 mL YPD in 5 mL disposable tube (Falcon, #352063), and grow at 30°C overnight.
   d. If desired, save each diploid strain in 17% glycerol in 96-well plate in -80°C.
   e. Transfer 125uL overnight culture to eppie and spin at 2500 rpm for 3 min.
   d. Re-suspend in 1mL Supplemented Sporulation Media (see Media Recipes) and place spinning in 5 mL disposable test tube (Falcon, #352063) at 25°C for 3 days.

3. Digesting diploids with zymolyase (modified from [Herman and Rine 1997]) and selecting haploids
   a. Transfer 50 uL sporulated cells to 1.7mL eppie.
   b. Spin 8,000 rpm for 1 min and remove supernatant.
   c. Re-suspend in 1 mL softening buffer (see recipe below), and incubate 30°C for 36 min.
   d. Spin at 8,000 rpm for 1 min and remove supernatant.
   e. Re-suspend in 200 uL spheroplasting buffer (see recipe below), add 2 uL 30 mg/mL 100T zymolyase (US Biological Sciences, #Z1004-25mg), and incubate 30°C for 3 hr.
   f. Spin 8,000 rpm for 1 min, re-suspend in 400 uL 0.5% Triton X-100 and vortex for 15 sec.
   g. Plate 30 uL to "MATα/Double-Barcode/Double-Deletion Selection Plates" (see Media Recipes).
   h. After 3-4 days, pick single colony of each strain and save in 17% glycerol in 96-well plate in -80°C.
   i. If desired, for a subset of strains verify mating type.
   j. If desired, for subset of strains, extract DNA and verify iSeq double barcode sequence using the following PCR, exoSAP, and send for Sanger sequencing with P19 (sequence of product in Appendix):

      Amount of each reagent per tube:
      vi. 12.5 uL OneTaq 2X MM (NEB, #M0482S)
      vii. 0.5 uL Forward Primer (10uM stock of P18, see Appendix for sequence)
      viii. 0.5 uL Reverse Primer (10uM stock of P19, see Appendix for sequence)
      ix. 0.5 uL DNA
      x. 11 uL H_2O

      Cycling conditions (perform 30 cycles for steps ii-iv):
      vii. 94°C, 30 sec
      viii. 94°C, 20 sec
      ix. 50°C, 30 sec
      x. 68°C, 45 sec
      xi. 68°C, 5 min
      xii. 4°C, Inf

Softening buffer: 10 mM dithiothreitol, 100 mM Tris-SO_4, pH 9.4
Spheroplasting buffer: 2.1 M sorbitol, 10 mM potassium phosphate, pH 7.2

Pooled Growth

Pooling mutants from frozen stocks for aliquots to inoculate pooled cultures:

1. Frog strains from 96-well plates into 1.2 mL liquid YPD in 2 mL 96-deep-well plates (Fisher, #12-565-605) using sterile metal pinning tool, cover in sterile velvet and foil/saran wrap.
2. Grow at 30°C until saturated, about 2-3 days.
3. Pool the saturated strains in a large, sterile beaker.
4. Shake in incubator for 20 min at 230 rpm to mix thoroughly.
5. Take cellular concentration using a Beckman Coulter Counter.
6. Concentrate cell mixture 3-fold by spinning in 50 mL tubes and removing supernatant.
7. Aliquot to tubes in 17% glycerol for starting cultures:
    a. Add 1mL cells + 340 uL 50% glycerol per orange top tube (Corning, 430289), mix well, and store at -80°C for future inocula.

Pooling WT control strains:

1. Streak the following control strains carrying iSeq Double Barcode system and deletions of dubious ORFS for single colonies on YPD and grow overnight at 30°C:
    a. "WT" control strains: 'ddHap53', 'ddHap54', 'ddHap58', 'ddHap59', 'ddHap363', 'ddHap364', 'ddHap369', 'ddHap370' (genotypes in Appendix, strains available upon request).
2. For each strain, use a single colony to inoculate 4 cultures of 5 mL YPD, and grow rotating overnight at 30°C.
3. Pool all 32 cultures into a sterile 500 mL flask.
4. Shake in incubator for 20 min at 230 rpm to mix thoroughly.
5. Take cellular concentration using a Beckman Coulter Counter.
6. Concentrate cell mixture 3-fold by spinning in 50 mL tubes and removing supernatant.
7. Aliquot to tubes in 17% glycerol for starting cultures:
    a. Add 1mL culture + 340 uL 50% glycerol per orange top tube (Corning, 430289), mix well, and store at -80°C for future inocula.

YPD Batch Culture Protocol:

For each replicate culture (at least three are recommended):

1. Inoculate 100 mL YPD in 500 mL flask (without baffles) with 1.125×10^9 cells:
    a. Cell mixture should be made by mixing the two pools generated above, WT and mutant, at a ratio of 50:50.
2. Grow shaking at 230 rpm at 30°C for 24 hours
3. Pre-heat 87.5 mL fresh YPD in 500 mL flask at 30°C for 1 hour before transfer.
4. After the 24 hours of growth, transfer 12.5 mL saturated culture to the 87.5 mL pre-heated YPD and place shaking in incubator at 230 rpm at 30°C for 24 hours.
5. With remaining culture, set aside 1 mL to:
    a. Take cellular concentration with Beckman Coulter Counter.
    b. Perform serial dilutions and platings to YPD to verify cellular concentration.
    c. Use microscope to observe culture for contamination.
6. Split remaining culture (~86 mL) into two 50 mL conical tubes (Corning, #430291) and re-suspend in 5 mL sorbitol solution (recipe below). Save cells in solution at -20°C for DNA extraction.
7. Repeat steps 3-7 for desired number of transfers (at least four time points are recommended).

Sorbitol solution: 0.9 M sorbitol, 0.1 M EDTA, 0.1 M Tris-HCl

Barcode Sequencing and Analysis

DNA Spooling

1. Thaw 5 ml cells/sorbitol solution (stored in 50 mL conical tube) at room temperature.
2. Add 2 uL of 30 mg/mL 100T zymolyase (US Biological Sciences, #Z1004-25mg) and 5ul β-mercaptoethanol to thawed cells
3. Incubate at 37°C for 1-3 hours. Monitor the digest by microscopy every 30-60 minutes and stop digest when 40-50% of cells are spheroplasted (i.e. they appear to lyse with addition on 10% SDS).
4. When ready, spin spheroplasted cells gently at 1500 rpm for 7 minutes and re-suspend in 5 ml Tris/EDTA solution (recipe below).
5. Add 500 uL 10% SDS, invert tube gently several times to mix. Solution should become viscose as cells lyse.
6. Incubate 20 min at 65°C in a water bath.
7. Add 2 ml 5 M Potassium Acetate, and shake gently to mix. Set on ice for at least 30 min.
8. Spin 10 min at 4°C at 10,000 rpm. Pour supernatant into a new 50 ml conical through a sterile cheesecloth.
9. Precipitate DNA with 15 ml 100% ethanol for 5 min at room temperature.
10. Spin at 4°C for 10 min at 10,000 rpm.
11. Pour off supernatant, air-dry pellet, re-suspend in 500 uL TE (see recipe below), and transfer to a 2 mL eppie.
12. Add 50 uL of 10 mg/ml RNase A and incubate for 15 min at 65°C.
13. Precipitate DNA by adding 40 uL of 3 M Sodium Acetate and 1 mL 100% ethanol, and mix gently by inversion.
14. Spool DNA with glass rod (Fisher, #13-707-46), dry, and re-suspend in 500 uL TE (see recipe below) in a new 2 mL eppie.
15. Precipitate again by adding 40 uL of 3M Sodium Acetate and 1mL 100% ethanol.
16. Spool again, dry pellet, and re-suspend in 200 uL EB (10 mM Tris-Cl, pH 8.5) in 1.7 mL eppie.
17. Incubate at 65°C for up to 2 hours to help with re-suspension.
18. Spin to pellet cellular debris and measure DNA concentration in supernatant with Qubit (Thermo Fisher, #Q33216).
19. Store at -20°C for future use.

Recipes:
Tris/EDTA Solution: 2.5 mL 1M Tris-HCl, pH 7.5 + 2 mL 0.5 M EDTA, pH 8.0 + 45.5 mL H_2O
TE: 0.5 mL 1M Tris-HCl, pH 7.5 + 0.1 mL 0.5 M EDTA, pH 8.0 + 49.4 mL H_2O

2-Step PCR to Generate Sequencing Libraries

1. Thaw purified DNA, spin to pellet cellular debris, and dilute to a concentration of 50 ng/uL in EB (10 mM Tris-Cl, pH 8.5)
2. Perform the following PCR in each of 24 tubes:

   Amount of each reagent per tube:
   i. 25 uL OneTaq 2X MM (NEB, #M0482S)
   ii. 1 uL Forward Primer (10 uM stock of P44, see Appendix for sequence)
   iii. 1 uL Reverse Primer (10 uM stock of P37, see Appendix for sequence)
   iv. 12 uL DNA (50 ng/uL from Step #1)
   v. 11 uL H_2O

   Cycling conditions (perform 3 cycles for steps ii-iv):
   i. 94°C, 10 min
   ii. 94°C, 3 min
   iii. 55°C, 1 min
   iv. 68°C, 1 min
   v. 68°C, 5 min
   vi. 4°C, Inf

3. Purify 6 PCR reactions per column from the QIAquick PCR Purification Kit (QIAGEN, #28106), a total of 4 columns, eluting each in 50 uL EB (10 mM Tris-Cl, pH 8.5).

4. Perform the following PCR in each of 12 tubes:

   Amount of each reagent per tube:
   i. 25 uL 2X PrimeSTAR Max (Clontech, #R045A)
   ii. 1 uL Forward Primer (10 uM stock of PE1, see Appendix for sequence)
   iii. 1 uL Reverse Primer (10 uM stock of PE2, see Appendix for sequence)
   iv. 15 uL DNA (Purified PCR rxn from Step #3)
   v. 8 uL H_2O

   Cycling conditions (perform 24 cycles for steps ii-iv):
   i. 98°C, 2 min
   ii. 98°C, 10 sec
   iii. 69°C, 15 sec
   iv. 72°C, 15 sec
   v. 72°C, 1 min
   vi. 4°C, Inf

5. Purify all 12 PCR reactions into 1 PCR purification column, eluting in 50 uL EB (10 mM Tris-Cl, pH 8.5)
6. Use E-gel (Thermo Fisher, #G6465 and #G6610-02) to purify band of prepared library (~350 bp).
7. Pool reactions carrying unique multiplexing tags (added in PCR in Step #2) to be sequenced on the same Illumina run.
8. Quantify DNA concentration with Qubit (Thermo Fisher, #Q33216).
9. Use Bioanalyzer (Agilent 2100) to validate DNA concentration and quantify size distribution of library.
10. Sequence using Illumina DNA sequencing platform (i.e. HiSeq 2000, paired-end 100 bp reads).

Data Analysis

We input (1) the fastq files from each Illumina run, (2) the sequences of the multiplexing tags added in the first PCR, and, (3) a list of known double barcode sequences for the strains in the pooled culture, to custom R and Python scripts which outputs counts of each double barcode for each de-multiplexed sample. These scripts are available upon request. Custon R scripts used to estimate fitness and interaction scores from these counts are also available upon request.

Media Recipes:

YPD (only add agar if making plates)
For 500 mL:
- 5g Yeast Extract (Difco) (Fisher, #DF0127-17-9)
- 10g Bacto Peptone (Difco) (Fisher, #DF0118-17-0)
- 10g Bacto Agar (Difco) (Fisher, #DF0140-01-0)

→up to 475 mL with water
→autoclave and when cool add 25 mL filter sterilized 40% Dextrose (Fisher, #DF0155-07-6) and pour plates

YPD+Hyg (Final concentration: 100ug/mL)
Same as YPD, adding 3 mL of 50 mg/mL Hygromycin B Solution (Fisher, #MT-30-240-CR) when cool

YPD+Nat (Final concentration: 100ug/mL)
Same as YPD, adding 500 uL 100 mg/mL Nuorseothrocin (Jena Biosciences, #AB-101L) when cool

YPD+G418 (Final concentration: 200ug/mL)
Same as YPD, adding 500 uL 200 mg/mL G418 stock when cool

SC-URA+Gal
For 500 mL:
- 3.35g Yeast Nitrogen Base w/o amino acids (Fisher, #DF0919-15-3)
- 10g Bacto Agar (Difco) (Fisher, #DF0140-01-0)

→up to 450 mL with water
→autoclave and when cool add 50 mL of 20% Galactose and 0.7g of the following bulk mixture of amino acids: 1g Adenine sulfate, 4g L-Tryptophan, 1g L-Histidine-HCl, 1g L-Arginine-HCl, 1g L-Methionine, 3g L-Tyrosine, 4g L-Leucine, 4g L-Isoleucine, 3g L-Lysine-HCl, 2.5g L-Phenylalanine, 5g L-Glutamic acid, 5g L-Aspartic acid, 7.5g L-Valine, 10g L-Threonine, 20g L-Serine
→pour plates

Supplemented Sporulation Media:
For 200 mL:
- 20 mL 10% Potassium Acetate
- 2 mL 0.5% Zinc Acetate
- 2 mL 2 mg/mL Uracil
- 0.8 mL 5 mg/mL L-Histidine-HCl
- 1.2 mL 10 mg/mL L-Leucine
- Up to 200 mL sterilized H_2O

MATα/Single-Barcode/Single-Deletion Selection Plates
For 500 mL total:
- 10g Bacto Agar (Difco) (Fisher, #DF0140-01-0)

→bring up to 350 mL with H_2O
→autoclave and when cool add the following:
- 50 mL of 17g/L filter sterilized stock of yeast nitrogen base w/o amino acids and ammonium sulfate (Fisher, #DF0335-08-8)
- 16.67 mL of 30 g/L filter sterilized stock of monosodium glutamic acid
- 25 mL 40% Dextrose
- 3 mL Hygromycin B (50 mg/mL stock)
- 5 mL Canavanine (6 mg/mL stock)
- 0.54g of the following bulk mixture of amino acids: 1g L-Methionine, 3g L-Tyrosine, 4g L-Isoleucine, 2.5g L-Phenylalanine, 5g L-Glutamic acid, 5g L-Aspartic acid, 7.5g L-Valine, 10g L-Threonine, 20g L-Serine
- 5 mL ea of: Uracil (2 mg/mL stock), L-Tryptophan (5 mg/mL stock), L-Histidine-HCl (5 mg/mL stock)
- 500 uL Nuorseothrocin (100 mg/mL stock)

→up to 500 mL with sterile H_2O and pour plates

MATa/Single-Barcode/Single-Deletion Selection Plates
For 500 mL total:
- 10g Bacto Agar (Difco) (Fisher, #DF0140-01-0)

→bring up to 350 mL with H_2O
→autoclave and when cool add the following:
- 50 mL of 17g/L filter sterilized stock of yeast nitrogen base w/o amino acids and ammonium sulfate (Fisher, #DF0335-08-8)
- 16.67 mL of 30 g/L filter sterilized stock of monosodium glutamic acid
- 25 mL 40% Dextrose
- 3 mL Hygromycin B (50 mg/mL stock)
- 5 mL Canavanine (6 mg/mL stock)
- 0.54g of the following bulk mixture of amino acids: 1g L-Methionine, 3g L-Tyrosine, 4g L-Isoleucine, 2.5g L-Phenylalanine, 5g L-Glutamic acid, 5g L-Aspartic acid, 7.5g L-Valine, 10g L-Threonine, 20g L-Serine
- 5 mL ea of: Uracil (2 mg/mL stock), L-Tryptophan (5 mg/mL stock), L-Leucine (10 mg/mL stock)
- 500 uL G418 (200 mg/mL stock)

→up to 500 mL with sterile H_2O and pour plates

MATα/Double-Barcode/Double-Deletion Selection Plates
For 500 mL total:
- 10g Bacto Agar (Difco) (Fisher, #DF0140-01-0)

→bring up to 350 mL with H_2O
→autoclave and when cool add the following:
- 50 mL of 17g/L filter sterilized stock of yeast nitrogen base w/o amino acids and ammonium sulfate (Fisher, #DF0335-08-8)
- 16.67 mL of 30 g/L filter sterilized stock of monosodium glutamic acid
- 25 mL 40% Dextrose
- 500 ul Nuorseothrocin (100 mg/mL stock)
- 500 ul G418 (200 mg/mL stock)
- 0.54g of the following bulk mixture of amino acids: 1g L-Methionine, 3g L-Tyrosine, 4g L-Isoleucine, 2.5g L-Phenylalanine, 5g L-Glutamic acid, 5g L-Aspartic acid, 7.5g L-Valine, 10g L-Threonine, 20g L-Serine
- 5mL L-Histidine-HCl (5 mg/mL stock)
- 5mL L-Tryptophan (5 mg/mL stock)

→up to 500 mL with sterile H_2O and pour plates

Appendix

Primer Sequences

P7: 5′GCCGAAATTGCCAGGATCAGG3′
P8:
5′CCAGCTGGTACCNNNNNAANNNNNTTNNNNNTTNNNNNATAACTTCGTATAGCATACATTATACGAACGGTAGGC
GCGCCGGCCGCAAAT3′
P9:
5′CCAGCTGGTACCNNNNNAANNNNNAANNNNNTTNNNNNATAACTTCGTATAATGTATGCTATACGAACGGTAGGC
GCGCCGGCCGCAAAT3′
P10: 5′GTTCTTTGCTTTTTTTCCCCAACGACGTCGAACACATTAGTCCTACGCACTTAACTTCGCATCTG3′
P12: 5′GCTTGCGCTAACTGCGAACAGAGTGCCCTATGAAATAGGGGAATGCGCACTTAACTTCGCATCTG3′
P18: 5′CCCGCAGAGTACTGCAATTT3′
P19: 5′TGCACGAAAAGCAAACAAAC3′
P22: 5′CAACCTGAAGTCTAGGTCCCTATT3′
P27: 5′GCGCCTACCGTTCGTATAAT3′
P28: 5′CGCCTACCGTTCGTATAGCA3′
P44:
5’ACACTCTTTCCCTACACGACGCTCTTCCGATCTNNNNNNNNTGACCATTAATATGGACTAAAGGAGGCTTTT3′

Note for P44: sequence in red is the multiplexing tag and primers with different tag sequences can be ordered for use on different DNA samples, to allow for pooling multiple time points on the same sequencing lane. (Other 6 bp tags previously used include: GCCAAT, ATCACG, CAGATC, GGCTAC, and TAGCTT)

P37:
5’CTCGGCATTCCTGCTGAACCGCTCTTCCGATCTNNNNNNNNTATATACGCTCGAATTCAAGCTTAGATCTGATA3′

Note for P37: sequence in red is the multiplexing tag and primers with different tag sequences can be ordered for use on different DNA samples, to allow for pooling multiple time points on the same sequencing lane. (Other 6 bp tags previously used include: CGCTCT, GAGACG, ACTAGC, TGAGCT, CTGCTA, GCGTAC)

PE1: 5′CAAGCAGAAGACGGCATACGAGATCGGTCTCGGCATTCCTGCTGAACCGCTCTTCCGATCxT3′
PE2: 5′AATGATACGGCGACCACCGAGATCTACACTCTTTCCCTACACGACGCTCTTCCGATCxT3′

Strains:

HR28: *MATα ura3Δ0 leu2Δ0 his3Δ1 lys2Δ0* YBR209WΔ*::Gal-Cre-NatMX[W] CAN1Δ::MFA1pr-HIS3-MFα1pr-LEU2*

Sequence at YBR209W (future iSeq BC locus) in HR28:

Key:
NNNN = Gal promoter
NNNN = Cre
NNNN = CYC1 Terminator
NNNN = *NatMX*
NNNN = YBR209W genomic sequence

TGGGAGCTTGATGACTTGTGGAAATCAATCCAGTCTTGGATTGACCAACCTAACGATATTTCAGCAGTTGT
ATCGGAACTAACTGTCATTTTTTAATCAAAACTGAAATTTATTGTGACTATAAAAAAGATATTTCTATTTT
TTATCTTTTATATTAATGTAAATTGAAATTTGGTGTATATCAGCTGCTCCACTGATATATTAATATAAGCT
TATAAAAACTTGAGCCTATATGATCCTAGAAAAAAAAAGCTTAGCTAAGAGAGCCTGATATTCAGAAGTGA
GCGAGTATACAAATCTCACACCTCATCTGCTCTAGTGTTTAAATACAAACATTCAATTTCCAACAACGTCT
AACAAAGCTCGATTCAAATTAAGAAGCAGGAAAAAGCAAAACATCTGCGTGTCTTATCTCAATGGCTTGCG
CTAACTGCGAACAGAGTGCCCTATGAAATAGGGGAATGGTTCTTTGCTTTTTTTCCCCAACGACGTCGAAC
ACATTAGTCCTAAGCTAGCtagtacggattagaagccgccgagcgggtgacagccctccgaaggaagactc
tcctccgtgcgtcctcgtcttcaccggtcgcgttcctgaaacgcagatgtgcctcgcgccgcactgctccg
aacaataaagattctacaatactagcttttatggttatgaagaggaaaaattggcagtaacctggccccac
aaaccttcaaatgaacgaatcaaattaacaaccataggatgataatgcgattagtttttttagccttatttc
tggggtaattaatcagcgaagcgatgatttttgatctattaacagatatataaatgcaaaaactgcataac

cactttaactaatactttcaacattttcggtttgtattacttcttattcaaatgtaataaaagtatcaaca
aaaaattgttaatatacctctatactttaacgtcaaggagaaaaaaccccggattctagaactagtggatc
ccccgggctgcaggaattcgatatcaagcttatcgataccgtcgaggggcagagccgatcctgtacacttt
acttaaaaccattatctgagtgttaaatgtccaatttactgaccgtacaccaaaatttgcctgcattaccg
gtcgatgcaacgagtgatgaggttcgcaagaacctgatggacatgttcagggatcgccaggcgttttctga
gcatacctggaaaatgcttctgtccgtttgccggtcgtgggcggcatggtgcaagttgaataaccggaaat
ggtttcccgcagaacctgaagatgttcgcgattatcttctatatcttcaggcgcgcggtctggcagtaaaa
actatccagcaacatttgggccagctaaacatgcttcatcgtcggtccgggctgccacgaccaagtgacag
caatgctgtttcactggttatgcggcggatccgaaaagaaaacgttgatgccggtgaacgtgcaaaacagg
ctctagcgttcgaacgcactgatttcgaccaggttcgttcactcatggaaaatagcgatcgctgccaggat
atacgtaatctggcatttctggggattgcttataacaccctgttacgtatagccgaaattgccaggatcag
ggttaaagatatctcacgtactgacggtgggagaatgttaatccatattggcagaacgaaaacgctggtta
gcaccgcaggtgtagagaaggcacttagcctgggggtaactaaactggtcgagcgatggatttccgtctct
ggtgtagctgatgatccgaataactacctgttttgccgggtcagaaaaaatggtgttgccgcgccatctgc
caccagccagctatcaactcgcgccctggaagggatttttgaagcaactcatcgattgatttacggcgcta
aggatgactctggtcagagatacctggcctggtctggacacagtgcccgtgtcggagccgcgcgagatatg
gcccgcgctggagtttcaataccggagatcatgcaagctggtggctggaccaatgtaaatattgtcatgaa
ctatatccgtaccctggatagtgaaacaggggcaatggtgcgcctgctggaagatggcgattagccattaa
cgcgtaaatgattgctataattatttgatatttatggtgacatatgagaaaggatttcaacatcgacggaa
aatatgtagtgctgtctgtaagcactaatattcagtcgccagccgtcattgtcactgtaaagctgagcgat
agaatgcctgatattgactcaatatccgttgcgtttcctgtcaaaagtatgcgtagtgctgaacatttcgt
gatgaatgccaccgaggaagaagcacggcgcggttttgctaaagtgatgtctgagtttggcgaactcttgg
gtaaggttggaattgtcgacctcgagtcatgtaattagttatgtcacgcttacattcacgccctcccccca
catccgctctaaccgaaaaggaaggagttagacaacctgaagtctaggtccctatttattttttttatagtt
atgttagtattaagaacgttatttatatttcaaatttttctttttttttctgtacagacgcgtgtacgcatg
taacattatactgaaaaccttgcttgagaaggttttgggacgctcgaaggctttaatttgcggccGGCGCG
CCAGATCTGTTTAGCTTGCCTcGTCCCCGCCGGGTCACCCGGCCAGCGACATGGAGGCCCAGAATACCCTC
CTTGACAGTCTTGACGTGCGCAGCTCAGGGGCATGATGTGACTGTCGCCCGTACATTTAGCCCATACATCC
CCATGTATAATCATTTGCATCCATACATTTTGATGGCCGCACGGCGCGAAGCAAAAATTACGGCTCCTCGC
TGCAGACCTGCGAGCAGGGAAACGCTCCCCTCACAGACGCGTTGAATTGTCCCCACGCCGCGCCCCTGTAG
AGAAATATAAAAGGTTAGGATTTGCCACTGAGGTTCTTCTTTCATATACTTCCTTTTAAAATCTTGCTAGG
ATACAGTTCTCACATCACATCCGAACATAAACAACCATGGGTACCACTCTTGACGACACGGCTTACCGGTA
CCGCACCAGTGTCCCGGGGGACGCCGAGGCCATCGAGGCACTGGATGGGTCCTTCACCACCGACACCGTCT
TCCGCGTCACCGCCACCGGGGACGGCTTCACCCTGCGGGAGGTGCCGGTGGACCCGCCCCTGACCAAGGTG
TTCCCCGACGACGAATCGGACGACGAATCGGACGACGGGGAGGACGGCGACCCGGACTCCCGGACGTTCGT
CGCGTACGGGGACGACGGCGACCTGGCGGGCTTCGTGGTCGTCTCGTACTCCGGCTGGAACCGCCGGCTGA
CCGTCGAGGACATCGAGGTCGCCCCGGAGCACCGGGGGCACGGGGTCGGGCGCGCGTTGATGGGGCTCGCG
ACGGAGTTCGCCCGCGAGCGGGGCGCCGGGCACCTCTGGCTGGAGGTCACCAACGTCAACGCACCGGCGAT
CCACGCGTACCGGCGGATGGGGTTCACCCTCTGCGGCCTGGACACCGCCCTGTACGACGGCACCGCCTCGG
ACGGCGAGCAGGCGCTCTACATGAGCATGCCCTGCCCCTAATCAGTACTGACAATAAAAAGATTCTTGTTT
TCAAGAACTTGTCATTTGTATAGTTTTTTTATATTGTAGTTGTTCTATTTTAATCAAATGTTAGCGTGATT
TATATTTTTTTTCGCCTCGACATCATCTGCCATGTTGCCTTTGAGGAGACTGCAGCAAGAAAAAAAGTTCA
ATAAGGATTGCAAAAAAAAAAAGAAGCTCGCACTCAGGATCGAACTAAGGACCAACAGATTTGCAATCTGC
TGCGCTACCACTGCGCCATACGAGCTTTCTATTACATGTGGAAGACTAATATAGATAGTATAGTCAATTA
TACGAAGTAGTGCCAGCCCCACATATAAGAGAACGGTGGAATATTTGAACTGTATGCAAAGAAGTTTCAGC
GGGGACCTTTATTTTAGAAAAAGCGAATAAATGTCGCTCATTTGTTTAAACGGTATTTGTTGGACTGAGGG
TTGCTTCCATACGGAAATGCTAACGAAGAGGATGTGCTGAATTTTGCTATGTTTGGAATGGGCTCCTCCAT
CTGGGAATGATAATCATTTTTATTATTGTGTGTTCAAATACACTTACACATCTGTTTTTTCGTGGTAAGGA
ATCCGAG

HR27: *MATα ura3Δ0 leu2Δ0 his3Δ1 lys2Δ0* YBR209WΔ*::Gal-Cre-NatMX[C] CAN1Δ::MFA1pr-HIS3-MFα1pr-LEU2*

Sequence at YBR209W (future iSeq BC locus HR27):

Key:

NNNN = Gal promoter
NNNN = Cre
NNNN = CYC1 Terminator
NNNN = *NatMX*
NNNN = YBR209W genomic sequence

CACCTTGACAGGAAAAGACGCCCTTTGTTGGCTTTAGGCCAATCAGGTTGTTCAAGGCGGCTGGGACTCTA
CCAGAACCTGCTGTATCAGTACCCAATGCAATTGGTACGATACCTCTGGCGACCACCGATGCTGACCCAGC
GGAGGAACCACCAGATACATGCTCTTTGCTAAAAGCGCAAGGTGTTTTCCCATATGGAGACCGTGTGCCGA
CTAATCCTGTGGCAAATTGGTCCAAGTTTGTCTTACCCACGATTATCGCACCTGCATTTCTTAGTAGTTCT
ACTACTTTAGAGTCTTTGGAAGGCTCATATGCAAAGGATGGACATGCAGCGGTGGTGGGTAGACCTCTAAC
GTCGATGTTGTCCTTAACAGCAATAGGGACACCGTAGAGAGGTAGAGTTTCTTTATTTTCTCTGCTCTTTA
AAATTTGGAATTGGTGCAGTAAATTTTCCTTTGAAATTAGCGATATCCACGCATTATCGACTGGCGCAACA
TTTTGAGAGTCTAGTAGTGATTCAAGAAGCCTTAGTGAAGCCTGGGAGCTTGATGACTTGTGGAAATCAAT
CCAGTCTTGGATTGACCAACCTAACGATATTTCAGCAGTTGTATCGGAACTAACTGTCATTTTTTAATCAA
AACTGAAATTTATTGTGACTATAAAAAAGATATTTCTATTTTTTATCTTTTATATTAATGTAAATTGAAAT
TTGGTGTATATCAGCTGCTCCACTGATATATTAATATAAGCTTATAAAAACTTGAGCCTATATGATCCTAG
AAAAAAAAAGCTTAGCTAAGAGAGCCTGATATTCAGAAGTGAGCGAGTATACAAATCTCACACCTCATCTG
CTCTAGTGTTTAAATACAAACATTCAATTTCCAACAACGTCTAACAAAGCTCGATTCAAATTAAGAAGCAG
GAAAAAGCAAAACATCTGCGTGTCTTATCTCAATGGCTTGCGCTAACTGCGAACAGAGTGCCCTATGAAAT
AGGGGAATGCGCACTTAACTTCGCATCTGGGCAGATGATGTCGAGGCGAAAAAAAATATAAATCACGCTAA
CATTTGATTAAAATAGAACAACTACAATATAAAAAAACTATACAAATGACAAGTTCTTGAAAACAAGAATC
TTTTTATTGTCAGTACTGATTAGGGGCAGGGCATGCTCATGTAGAGCGCCTGCTCGCCGTCCGAGGCGGTG
CCGTCGTACAGGGCGGTGTCCAGGCCGCAGAGGGTGAACCCCATCCGCCGGTACGCGTGGATCGCCGGTGC
GTTGACGTTGGTGACCTCCAGCCAGAGGTGCCCGGCGCCCCGCTCGCGGGCGAACTCCGTCGCGAGCCCCA
TCAACGCGCGCCCGACCCCGTGCCCCCGGTGCTCCGGGGCGACCTCGATGTCCTCGACGGTCAGCCGGCGG
TTCCAGCCGGAGTACGAGACGACCACGAAGCCCGCCAGGTCGCCGTCGTCCCCGTACGCGACGAACGTCCG
GGAGTCCGGGTCGCCGTCCTCCCCGTCGTCCGATTCGTCGTCCGATTCGTCGTCGGGGAACACCTTGGTCA
GGGGCGGGTCCACCGGCACCTCCCGCAGGGTGAAGCCGTCCCCGGTGGCGGTGACGCGGAAGACGGTGTCG
GTGGTGAAGGACCCATCCAGTGCCTCGATGGCCTCGGCGTCCCCCGGGACACTGGTGCGGTACCGGTAAGC
CGTGTCGTCAAGAGTGGTACCCATGGTTGTTTATGTTCGGATGTGATGTGAGAACTGTATCCTAGCAAGAT
TTTAAAAGGAAGTATATGAAAGAAGAACCTCAGTGGCAAATCCTAACCTTTTATATTTCTCTACAGGGGCG
CGGCGTGGGGACAATTCAACGCGTCTGTGAGGGGAGCGTTTCCCTGCTCGCAGGTCTGCAGCGAGGAGCCG
TAATTTTTGCTTCGCGCCGTGCGGCCATCAAAATGTATGGATGCAAATGATTATACATGGGGATGTATGGG
CTAAATGTACGGGCGACAGTCACATCATGCCCCTGAGCTGCGCACGTCAAGACTGTCAAGGAGGGTATTCT
GGGCCTCCATGTCGCTGGCCGGGTGACCCGGCGGGGACgAGGCAAGCTAAACAGATCTGGCGCGCCggccg
caaattaaagccttcgagcgtcccaaaaccttctcaagcaaggttttcagtataatgttacatgcgtacac
gcgtctgtacagaaaaaaaagaaaaatttgaaatataaataacgttcttaatactaacataactataaaaa
aataaatagggacctagacttcaggttgtctaactccttccttttcggttagagcggatgtgggggaggg
cgtgaatgtaagcgtgacataactaattacatgactcgaggtcgacaattccaaccttacccaagagttcg
ccaaactcagacatcactttagcaaaccgcgccgtgcttcttcctcggtggcattcatcacgaaatgttc
agcactacgcatacttttgacaggaaacgcaacggatattgagtcaatatcaggcattctatcgctcagct
ttacagtgacaatgacggctggcgactgaatattagtgcttacagacagcactacatattttccgtcgatg
ttgaaatcctttctcatatgtcaccataaatatcaaataattatagcaatcatttacgcgttaatggctaa
tcgccatcttccagcaggcgcaccattgcccctgtttcactatccagggtacggatatagttcatgacaat
atttacattggtccagccaccagcttgcatgatctccggtattgaaactccagcgcgggccatatctcgcg
cggctccgacacgggcactgtgtccagaccaggccaggtatctctgaccagagtcatccttagcgccgtaa
atcaatcgatgagttgcttcaaaaatcccttccagggcgcgagttgatagctggctggtggcagatggcgc
ggcaacaccatttttctgacccggcaaaacaggtagttattcggatcatcagctacaccagagacggaaa
tccatcgctcgaccagtttagttacccccaggctaagtgccttctctacacctgcggtgctaaccagcgtt
ttcgttctgccaatatggattaacattctcccaccgtcagtacgtgagatatctttaaccctgatcctggc
aatttcggctatacgtaacagggtgttataagcaatccccagaaatgccagattacgtatatcctggcagc
gatcgctattttccatgagtgaacgaacctggtcgaaatcagtgcgttcgaacgctagagcctgttttgca
cgttcaccggcatcaacgttttcttttcggatccgccgcataaccagtgaaacagcattgctgtcacttgg
tcgtggcagcccggaccgacgatgaagcatgtttagctggcccaaatgttgctggatagtttttactgcca

gaccgcgcgcctgaagatatagaagataatcgcgaacatcttcaggttctgcgggaaaccatttccggtta
ttcaacttgcaccatgccgcccacgaccggcaaacggacagaagcattttccaggtatgctcagaaaacgc
ctggcgatccctgaacatgtccatcaggttcttgcgaacctcatcactcgttgcatcgaccggtaatgcag
gcaaattttggtgtacggtcagtaaattggacatttaacactcagataatggttttaagtaaagtgtacag
gatcggctctgcccctcgacggtatcgataagcttgatatcgaattcctgcagcccgggggatccactagt
tctagaatccgggtttttctccttgacgttaaagtatagaggtatattaacaattttttgttgatactt
ttattacatttgaataagaagtaatacaaaccgaaaatgttgaaagtattagttaaagtggttatgcagtt
tttgcatttatatatctgttaatagatcaaaaatcatcgcttcgctgattaattaccccagaaataaggct
aaaaaactaatcgcattatcatcctatggttgttaatttgattcgttcatttgaaggtttgtggggccagg
ttactgccaatttttcctcttcataaccataaaagctagtattgtagaatctttattgttcggagcagtgc
ggcgcgaggcacatctgcgtttcaggaacgcgaccggtgaagacgaggacgcacggaggagagtcttcctt
cggagggctgtcacccgctcggcggcttctaatccgtactaGCTAGCTAAGGTTGAGCATTACGTATGATA
TGTAGGACTAATGTGTTCGACGTCGTTGGGGAAAAAAAGCAAAGAACATGTTGCCTTTGAGGAGACTGCAG
CAAGAAAAAAAGTTCAATAAGGATTGCAAAAAAAAAAAGAAGCTCGCACTCAGGATCGAACTAAGGACCAA
CAGATTTGCAATCTGCTGCGCTACCACTGCGCCATACGAGCTTTCTATTACATGTGGGAAGACTAATATAG
ATAGTATAGTCAATTATACGAAGTAGTGCCAGCCCCACATATAAGAGAACGGTGGAATATTTGAACTGTAT
GCAAAGAAGTTTCAGCGGGGACCTTTATTTTAGAAAAAGCGAATAAATGTCGCTCATTTGTTTAAACGGTA
TTTGTTGGACTGAGGGTTGCTTCCATACGGAAATGCTAACGAAGAGGATGTGCTGAATTTTGCTATGTTTG
GAATGGGCTCCTCCATCTGGGAATGATAATCATTTTTATTATTGTGTGTTCAAATACACTTACACATCTGT
TTTTTCGTGGTAAGGAATCCGAGAATCACATGAGGTTTGGGATAGTTGCTGGCAATCAATGAATGACATCA
GATAAAACGAAAGGAGAAATAATATGTTGATAATTAGAGGTTAAAAATTAGTATTAATGAAGAAATAATTA
CTGATCTTCTTATACTAAATAAGAGAGGTATATAAAACACACGCCGATTGGTCATATTAATCATGACCAAT
ATAATAGTGATTCCGGTAGTTACTATACATTGATGTGACGACTCATATTCCTCATATATGTACCTACCATA
ACATGTTCAACTAATAGGTCTTTAACACAGCTTCAGTATTGTCTGAGCTTCTCGTTTAACATTCCTTCTGC
AATAGGCGCAATCACACTTAAACGTATACGAGTTGTACATTAATATACGATGTAAGCATTGAATTGTTACC
ATAGCAACTCATGTCACTATTAATTACTCTCGTTCCAACATAATATTATTATGGAGTAATATCTATTCCCT
TTCGTGGATTC

HR296-1-A1: *MATa ura3*Δ0 *leu2*Δ0 *his3*Δ1 *met15*Δ0 YBR209WΔ*::(3' iSeq BC Construct) CAN1*Δ::*MFA1pr-HIS3-MFα1pr-LEU2*

MIA31: *MATa ura3*Δ0 *leu2*Δ0 *his3*Δ1 YFR054CΔ::*KanMX* YBR209WΔ*::(5' iSeq BC Construct) CAN1*Δ::*MFA1pr-HIS3-MFα1pr-LEU2*

ddHap53: *MATα ura3*Δ0 *leu2*Δ0 *his3*Δ1 YHR095WΔ::*NatMX* YFR054CΔ::*KanMX* YBR209WΔ::(*iSeq DBC Construct) CAN1*Δ::*MFA1pr-HIS3-MFα1pr-LEU2*

ddHap54: *MATα ura3*Δ0 *leu2*Δ0 *his3*Δ1 YHR095WΔ::*NatMX* YFR054CΔ::*KanMX* YBR209WΔ::(*iSeq DBC Construct) CAN1*Δ::*MFA1pr-HIS3-MFα1pr-LEU2*

ddHap58: *MATα ura3*Δ0 *leu2*Δ0 *his3*Δ1 YHR095WΔ::*NatMX* YFR054CΔ::*KanMX* YBR209WΔ::(*iSeq DBC Construct) CAN1*Δ::*MFA1pr-HIS3-MFα1pr-LEU2*

ddHap59: *MATα ura3*Δ0 *leu2*Δ0 *his3*Δ1 YHR095WΔ::*NatMX* YFR054CΔ::*KanMX* YBR209WΔ::(*iSeq DBC Construct) CAN1*Δ::*MFA1pr-HIS3-MFα1pr-LEU2*

ddHap363: *MATα ura3*Δ0 *leu2*Δ0 *his3*Δ1 YHR095WΔ::*KanMX* YFR054CΔ::*NatMX* YBR209WΔ::(*iSeq DBC Construct) CAN1*Δ::*MFA1pr-HIS3-MFα1pr-LEU2*

ddHap364: *MATα ura3*Δ0 *leu2*Δ0 *his3*Δ1 YHR095WΔ::*KanMX* YFR054CΔ::*NatMX* YBR209WΔ::(*iSeq DBC Construct) CAN1*Δ::*MFA1pr-HIS3-MFα1pr-LEU2*

ddHap369: *MATα ura3*Δ0 *leu2*Δ0 *his3*Δ1 YHR095WΔ::*KanMX* YFR054CΔ::*NatMX* YBR209WΔ::(*iSeq DBC Construct) CAN1*Δ::*MFA1pr-HIS3-MFα1pr-LEU2*

ddHap370: *MATα ura3*Δ0 *leu2*Δ0 *his3*Δ1 YHR095WΔ::*KanMX* YFR054CΔ::*NatMX* YBR209WΔ::(*iSeq DBC Construct) CAN1*Δ::*MFA1pr-HIS3-MFα1pr-LEU2*

Plasmids

M207/pBAR1

Key:
NNNN = Gal promoter
NNNN = Cre
NNNN = CYC1 Terminator
NNNN = *NatMX*
NNNN = P7 binding site
NNNN = P8 binding site

GAACGCGGCCGCCAGCTGAAGCTTCGTACGCTGCAGGTCGACGGATCCCCGGGTTAATTAAGGGCCCACAAGGTTTT
GCATTGAGGATAGTATAGAAGCAAGAATCATTGAATTACAGGAAAAAAAGGCAAATATGATTCATGCTACAATAAAC
CAAGATGAAGCTGCCATTAGCAGACTAACGCCAGCTGATTTACAGTTCTTATTCAATAACTAATATTTTATTCTCTT
ATTATATATTATTCTCGGAGTTTTTAAGTGACATCACCCGAAAAGAAGCTAAGTCTTTCTCCTAATTCATATTTAAT
TATTGTACATGGACATATCATACGTAATGCTCAACCTTAGCTAGCtagtacggattagaagccgccgagcgggtgac
agccctccgaaggaagactctcctccgtgcgtcctcgtcttcaccggtcgcgttcctgaaacgcagatgtgcctcgc
gccgcactgctccgaacaataaagattctacaatactagcttttatggttatgaagaggaaaaattggcagtaacct
ggccccacaaaccttcaaatgaacgaatcaaattaacaaccataggatgataatgcgattagttttttagccttatt
tctggggtaattaatcagcgaagcgatgatttttgatctattaacagatatataaatgcaaaaactgcataaccact
ttaactaatactttcaacattttcggtttgtattacttcttattcaaatgtaataaaagtatcaacaaaaaattgtt
aatatacctctatactttaacgtcaaggagaaaaaaccccggattctagaactagtggatcccccgggctgcaggaa
ttcgatatcaagcttatcgataccgtcgagggggcagagccgatcctgtacactttacttaaaaccattatctgagtg
ttaaatgtccaatttactgaccgtacaccaaaatttgcctgcattaccggtcgatgcaacgagtgatgaggttcgca
agaacctgatggacatgttcagggatcgccaggcgttttctgagcatacctggaaaatgcttctgtccgtttgccgg
tcgtgggcggcatggtgcaagttgaataaccggaaatggtttcccgcagaacctgaagatgttcgcgattatcttct
atatcttcaggcgcgcggtctggcagtaaaaactatccagcaacatttgggccagctaaacatgcttcatcgtcggt
ccgggctgccacgaccaagtgacagcaatgctgtttcactggttatgcggcggatccgaaaagaaaacgttgatgcc
ggtgaacgtgcaaaacaggctctagcgttcgaacgcactgatttcgaccaggttcgttcactcatggaaaatagcga
tcgctgccaggatatacgtaatctggcatttctggggattgcttataacaccctgttacgtatagccgaaattgcca
ggatcagggttaaagatatctcacgtactgacggtgggagaatgttaatccatattggcagaacgaaaacgctggtt
agcaccgcaggtgtagagaaggcacttagcctgggggtaactaaactggtcgagcgatggatttccgtctctggtgt
agctgatgatccgaataactacctgttttgccgggtcagaaaaaatggtgttgccgcgccatctgccaccagccagc
tatcaactcgcgccctggaagggatttttgaagcaactcatcgattgatttacggcgctaaggatgactctggtcag
agatacctggcctggtctggacacagtgcccgtgtcggagccgcgcgagatatggcccgcgctggagtttcaatacc
ggagatcatgcaagctggtggctggaccaatgtaaatattgtcatgaactatatccgtaccctggatagtgaaacag
gggcaatggtgcgcctgctggaagatggcgattagccattaacgcgtaaatgattgctataattatttgatatttat
ggtgacatatgagaaaggatttcaacatcgacggaaaatatgtagtgctgtctgtaagcactaatattcagtcgcca
gccgtcattgtcactgtaaagctgagcgatagaatgcctgatattgactcaatatccgttgcgtttcctgtcaaaag
tatgcgtagtgctgaacatttcgtgatgaatgccaccgaggaagaagcacggcgcggttttgctaaagtgatgtctg
agtttggcgaactcttgggtaaggttggaattgtcgacctcgagtcatgtaattagttatgtcacgcttacattcac
gccctccccccacatccgctctaaccgaaaaggaaggagttagacaacctgaagtctaggtccctatttattttttt
atagttatgttagtattaagaacgttatttatatttcaaatttttcttttttttctgtacagacgcgtgtacgcatg
taacattatactgaaaaccttgcttgagaaggttttgggacgctcgaaggctttaatttgcggccGGCGCGCCAGAT
CTGTTTAGCTTGCCTcGTCCCCGCCGGGTCACCCGGCCAGCGACATGGAGGCCCAGAATACCCTCCTTGACAGTCTT
GACGTGCGCAGCTCAGGGGCATGATGTGACTGTCGCCCGTACATTTAGCCCATACATCCCCATGTATAATCATTTGC
ATCCATACATTTTGATGGCCGCACGGCGCGAAGCAAAAATTACGGCTCCTCGCTGCAGACCTGCGAGCAGGGAAACG
CTCCCCTCACAGACGCGTTGAATTGTCCCCACGCCGCGCCCCTGTAGAGAAATATAAAAGGTTAGGATTTGCCACTG
AGGTTCTTCTTTCATATACTTCCTTTTAAAATCTTGCTAGGATACAGTTCTCACATCACATCCGAACATAAACAACC
ATGGGTACCACTCTTGACGACACGGCTTACCGGTACCGCACCAGTGTCCCGGGGGACGCCGAGGCCATCGAGGCACT
GGATGGGTCCTTCACCACCGACACCGTCTTCCGCGTCACCGCCACCGGGGACGGCTTCACCCTGCGGGAGGTGCCGG
TGGACCCGCCCCTGACCAAGGTGTTCCCCGACGACGAATCGGACGACGAATCGGACGACGGGGAGGACGGCGACCCG
GACTCCCGGACGTTCGTCGCGTACGGGGACGACGGCGACCTGGCGGGCTTCGTGGTCGTCTCGTACTCCGGCTGGAA
CCGCCGGCTGACCGTCGAGGACATCGAGGTCGCCCCGGAGCACCGGGGGCACGGGGTCGGGCGCGCGTTGATGGGGC
TCGCGACGGAGTTCGCCCGCGAGCGGGGCGCCGGGCACCTCTGGCTGGAGGTCACCAACGTCAACGCACCGGCGATC
CACGCGTACCGGCGGATGGGGTTCACCCTCTGCGGCCTGGACACCGCCCTGTACGACGGCACCGCCTCGGACGGCGA
GCAGGCGCTCTACATGAGCATGCCCTGCCCCTAATCAGTACTGACAATAAAAAGATTCTTGTTTTCAAGAACTTGTC

ATTTGTATAGTTTTTTTATATTGTAGTTGTTCTATTTTAATCAAATGTTAGCGTGATTTATATTTTTTTTCGCCTCG
ACATCATCTGCCCAGATGCGAAGTTAAGTGCGCAGAAAGTAATATCATGCGTCAATCGTATGTGAATGCTGGTCGCT
ATACTGCTGTCGATTCGATACTAACGCCGCCATCCAGTGTCGAAAACGAGCTCCATTAGTGAGTAACTCTGTGATAT
CTCTCTATAATTAGCAGTTTTTCACTGAAATTCCAGGAAAGGTAATAAACTCAGATTTTTTTTTATACTATTGGCTG
CTTGTTACTTATATATCTTGAACTTCTCCCAGCGGGTCTTCAAATACATTTGGGCGATGTTCATGTTCATTAGGCAG
GTATTTCGACATTGAGTCACACGCGAAAAACCGCCGGAATTTTTTATGTAATTGCAAGTGGAATTCCGCTGGCAAAA
CTATTGGGCCCGTTAACCTGCATTAATGAATCGGCCAACGCGCGGGGAGAGGCGGTTTGCGTATTGGGCGCTCTTCC
GCTTCCTCGCTCACTGACTCGCTGCGCTCGGTCGTTCGGCTGCGGCGAGCGGTATCAGCTCACTCAAAGGCGGTAAT
ACGGTTATCCACAGAATCAGGGGATAACGCAGGAAAGAACATGTGAGCAAAAGGCCAGCAAAAGGCCAGGAACCGTA
AAAAGGCCGCGTTGCTGGCGTTTTTCCATAGGCTCCGCCCCCCTGACGAGCATCACAAAAATCGACGCTCAAGTCAG
AGGTGGCGAAACCCGACAGGACTATAAAGATACCAGGCGTTTCCCCCTGGAAGCTCCCTCGTGCGCTCTCCTGTTCC
GACCCTGCCGCTTACCGGATACCTGTCCGCCTTTCTCCCTTCGGGAAGCGTGGCGCTTTCTCAATGCTCACGCTGTA
GGTATCTCAGTTCGGTGTAGGTCGTTCGCTCCAAGCTGGGCTGTGTGCACGAACCCCCCGTTCAGCCCGACCGCTGC
GCCTTATCCGGTAACTATCGTCTTGAGTCCAACCCGGTAAGACACGACTTATCGCCACTGGCAGCAGCCACTGGTAA
CAGGATTAGCAGAGCGAGGTATGTAGGCGGTGCTACAGAGTTCTTGAAGTGGTGGCCTAACTACGGCTACACTAGAA
GGACAGTATTTGGTATCTGCGCTCTGCTGAAGCCAGTTACCTTCGGAAAAAGAGTTGGTAGCTCTTGATCCGGCAAA
CAAACCACCGCTGGTAGCGGTGGTTTTTTTGTTTGCAAGCAGCAGATTACGCGCAGAAAAAAAGGATCTCAAGAAGA
TCCTTTGATCTTTTCTACGGGGTCTGACGCTCAGTGGAACGAAAACTCACGTTAAGGGATTTTGGTCATGAGATTAT
CAAAAAGGATCTTCACCTAGATCCTTTTAAATTAAAAATGAAGTTTTAAATCAATCTAAAGTATATATGAGTAAACT
TGGTCTGACAGTTACCAATGCTTAATCAGTGAGGCACCTATCTCAGCGATCTGTCTATTTCGTTCATCCATAGTTGC
CTGACTCCCCGTCGTGTAGATAACTACGATACGGGAGGGCTTACCATCTGGCCCCAGTGCTGCAATGATACCGCGAG
ACCCACGCTCACCGGCTCCAGATTTATCAGCAATAAACCAGCCAGCCGGAAGGGCCGAGCGCAGAAGTGGTCCTGCA
ACTTTATCCGCCTCCATCCAGTCTATTAATTGTTGCCGGGAAGCTAGAGTAAGTAGTTCGCCAGTTAATAGTTTGCG
CAACGTTGTTGCCATTGCTACAGGCATCGTGGTGTCACGCTCGTCGTTTGGTATGGCTTCATTCAGCTCCGGTTCCC
AACGATCAAGGCGAGTTACATGATCCCCCATGTTGTGCAAAAAAGCGGTTAGCTCCTTCGGTCCTCCGATCGTTGTC
AGAAGTAAGTTGGCCGCAGTGTTATCACTCATGGTTATGGCAGCACTGCATAATTCTCTTACTGTCATGCCATCCGT
AAGATGCTTTTCTGTGACTGGTGAGTACTCAACCAAGTCATTCTGAGAATAGTGTATGCGGCGACCGAGTTGCTCTT
GCCCGGCGTCAATACGGGATAATACCGCGCCACATAGCAGAACTTTAAAAGTGCTCATCATTGGAAAACGTTCTTCG
GGGCGAAAACTCTCAAGGATCTTACCGCTGTTGAGATCCAGTTCGATGTAACCCACTCGTGCACCCAACTGATCTTC
AGCATCTTTTACTTTCACCAGCGTTTCTGGGTGAGCAAAAACAGGAAGGCAAAATGCCGCAAAAAAGGGAATAAGGG
CGACACGGAAATGTTGAATACTCATACTCTTCCTTTTTCAATATTATTGAAGCATTTATCAGGGTTATTGTCTCATG
AGCGGATACATATTTGAATGTATTTAGAAAAATAAACAAATAGGGGTTCCGCGCACATTTCCCCGAAAAGTGCCACC
TGACGTCTAAGAAACCATTATTATCATGACATTAACCTATAAAAATAGGCGTATCACGAGGCCCTTTCGTCTCGCGC
GTTTCGGTGATGACGGTGAAAACCTCTGACACATGCAGCTCCCGGAGACGGTCACAGCTTGTCTGTAAGCGGATGCC
GGGAGCAGACAAGCCCGTCAGGGCGCGTCAGCGGGTGTTGGCGGGTGTCGGGGCTGGCTTAACTATGCGGCATCAGA
GCAGATTGTACTGAGAGTGCACCATATGGACATATTGTCGTTAGAACGCGGCTACAATTAATACATAACCTTATGTA
TCATACACATACGATTTAGGTGACACTATA

<u>M272</u>

Key:

NNNN = Gal promoter
NNNN = Truncated Cre
NNNN = CYC1 Terminator
NNNN = 3' *URA3*
NNNN = *HygMX*
NNNN = Artificial intron terminator

GAACGCGGCCGCCAGCTGAAGCTTCGTACGCTGCAGGTCGACGGATCCCCGGGTTAATTAAGGGCCCAATAGTTTTG
CCAGCGGAATTCCACTTGCAATTACATAAAAAATTCCGGCGGTTTTTCGCGTGTGACTCAATGTCGAAATACCTGCC
TAATGAACATGAACATCGCCCAAATGTATTTGAAGACCCGCTGGGAGAAGTTCAAGATATATAAGTAACAAGCAGCC
AATAGTATAAAAAAAAATCTGAGTTTATTACCTTTCCTGGAATTTCAGTGAAAAACTGCTAATTATAGAGAGATATC
ACAGAGTTACTCACTAATGGCTAGCtagtacggattagaagccgccgagcgggtgacagccctccgaaggaagactc
tcctccgtgcgtcctcgtcttcaccggtcgcgttcctgaaacgcagatgtgcctcgcgccgcactgctccgaacaat
aaagattctacaatactagcttttatggttatgaagaggaaaaattggcagtaacctggccccacaaaccttcaaat
gaacgaatcaaattaacaaccataggatgataatgcgattagttttttagccttatttctggggtaattaatcagcg
aagcgatgatttttgatctattaacagatatataaatgcaaaaactgcataaccactttaactaatactttcaacat
tttcggtttgtattacttcttattcaaatgtaataaaagtatcaacaaaaaattgttaatatacctctatactttaa
cgtcaaggagaaaaaaccccggattctagaactagtggatcccccgggctgcaggaattcgatatcaagcttatcga
ttgatttacggcgctaaggatgactctggtcagagatacctggcctggtctggacacagtgcccgtgtcggagccgc
gcgagatatggcccgcgctggagtttcaataccggagatcatgcaagctggtggctggaccaatgtaaatattgtca
tgaactatatccgtaAcctggatagtgaaacaggggcaatggtgcgcctgctggaagatggcgattagccattaacg
cgtaaatgattgctataattatttgatatttatggtgacatatgagaaaggatttcaacatcgacggaaaatatgta
gtgctgtctgtaagcactaatattcagtcgccagccgtcattgtcactgtaaagctgagcgatagaatgcctgatat
tgactcaatatccgttgcgtttcctgtcaaaagtatgcgtagtgctgaacatttcgtgatgaatgccaccgaggaag
aagcacggcgcggttttgctaaagtgatgtctgagtttggcgaactcttgggtaaggttggaattgtcgacctcgag
tcatgtaattagttatgtcacgcttacattcacgccctccccccacatccgctctaaccgaaaaggaaggagttaga
caacctgaagtctaggtccctatttatttttttatagttatgttagtattaagaacgttatttatatttcaaatttt
tctttttttttctgtacagacgcgtgtacgcatgtaacattatactgaaaaccttgcttgagaaggttttgggacgct
cgaaggctttaatttgcggccGGCGCGCCCTTAAGCAGGAGGGTACCGATATCAGATCTAAGCTTGAATTCGAATTT
TTACTAACAAATGGTATTATTTATAAcagatcttgactgatttttccatggagggcacagttaagccgctaaaggca
ttatccgccaagtacaattttttactcttcgaagacagaaaatttgctgacattggtaatacagtcaaattgcagta
ctctgcgggtgtatacagaatagcagaatgggcagacattacgaatgcacacggtgtggtgggcccaggtattgtta
gcggtttgaagcaggcggcagaagaagtaacaaaggaacctagaggccttttgatgttagcagaattgtcatgcaag
ggctccctatctactggagaatatactaagggtactgttgacattgcgaagagcgacaaagattttgttatcggctt
tattgctcaaagagacatgggtggaagagatgaaggttacgattggttgattatgacacccggtgtgggtttagatg
acaagggagacgcattgggtcaacagtatagaaccgtggatgatgtggtctctacaggatctgacattattattgtt
ggaagaggactatttgcaaagggaagggatgctaaggtagagggtgaacgttacagaaaagcaggctgggaagcata
tttgagaagatgcggccagcaaaactaaaaaactgtattataagtaaatgcatgtatactaaactcacaaattagag
cttcaatttaattatatcagttattaccGGTCACCCGGCCAGCGACATGGAGGCCCAGAATACCCTCCTTGACAGTC
TTGACGTGCGCAGCTCAGGGGCATGATGTGACTGTCGCCCGTACATTTAGCCCATACATCCCCATGTATAATCATTT
GCATCCATACATTTTGATGGCCGCACGGCGCGAAGCAAAAATTACGGCTCCTCGCTGCAGACCTGCGAGCAGGGAAA
CGCTCCCCTCACAGACGCGTTGAATTGTCCCCACGCCGCGCCCCTGTAGAGAAATATAAAAGGTTAGGATTTGCCAC
TGAGGTTCTTCTTTCATATACTTCCTTTTAAAATCTTGCTAGGATACAGTTCTCACATCACATCCGAACATAAACAA
CCATGGGTAAAAAGCCTGAACTCACCGCGACGTCTGTCGAGAAGTTTCTGATCGAAAAGTTCGACAGCGTCTCCGAC
CTGATGCAGCTCTCGGAGGGCGAAGAATCTCGTGCTTTCAGCTTCGATGTAGGAGGGCGTGGATATGTCCTGCGGGT
AAATAGCTGCGCCGATGGTTTCTACAAAGATCGTTATGTTTATCGGCACTTTGCATCGGCCGCGCTCCCGATTCCGG
AAGTGCTTGACATTGGGGAATTCAGCGAGAGCCTGACCTATTGCATCTCCCGCCGTGCACAGGGTGTCACGTTGCAA
GACCTGCCTGAAACCGAACTGCCCGCTGTTCTGCAGCCGGTCGCGGAGGCCATGGATGCGATCGCTGCGGCCGATCT
TAGCCAGACGAGCGGGTTCGGCCCATTCGGACCGCAAGGAATCGGTCAATACACTACATGGCGTGATTTCATATGCG
CGATTGCTGATCCCCATGTGTATCACTGGCAAACTGTGATGGACGACACCGTCAGTGCGTCCGTCGCGCAGGCTCTC
GATGAGCTGATGCTTTGGGCCGAGGACTGCCCCGAAGTCCGGCACCTCGTGCACGCGGATTTCGGCTCCAACAATGT
CCTGACGGACAATGGCCGCATAACAGCGGTCATTGACTGGAGCGAGGCGATGTTCGGGGATTCCCAATACGAGGTCG
CCAACATCTTCTTCTGGAGGCCGTGGTTGGCTTGTATGGAGCAGCAGACGCGCTACTTCGAGCGGAGGCATCCGGAG
CTTGCAGGATCGCCGCGGCTCCGGGCGTATATGCTCCGCATTGGTCTTGACCAACTCTATCAGAGCTTGGTTGACGG
CAATTTCGATGATGCAGCTTGGGCGCAGGGTCGATGCGACGCAATCGTCCGATCCGGAGCCGGGACTGTCGGGCGTA
CACAAATCGCCCGCAGAAGCGCGGCCGTCTGGACCGATGGCTGTGTAGAAGTACTCGCCGATAGTGGAAACCGACGC

CCCAGCACTCGTCCGAGGGCAAAGGAATAATCAGTACTGACAATAAAAAGATTCTTGTTTTCAAGAACTTGTCATTT
GTATAGTTTTTTTATATTGTAGTTGTTCTATTTTAATCAAATGTTAGCGTGATTTATATTTTTTTTCGCCTCGACAT
CATCTGCCCAGATGCGAAGTTAAGTGCGCAGAAAGTAATATCATGCGTCAATCGTATGTGAATGCTGGTCGCTATAC
TGCTGTCGATTCGATACTAACGCCGCCATCCAGTGTCGAAAACGAGCTCTAAGGTTGAGCATTACGTATGATATGTC
CATGTACAATAATTAAATATGAATTAGGAGAAAGACTTAGCTTCTTTTCGGGTGATGTCACTTAAAAACTCCGAGAA
TAATATATAATAAGAGAATAAAATATTAGTTATTGAATAAGAACTGTAAATCAGCTGGCGTTAGTCTGCTAATGGCA
GCTTCATCTTGGTTTATTGTAGCATGAATCATATTTGCCTTTTTTTCCTGTAATTCAATGATTCTTGCTTCTATACT
ATCCTCAATGCAAAACCTTGTGGGCCCGTTAACCTGCATTAATGAATCGGCCAACGCGCGGGGAGAGGCGGTTTGCG
TATTGGGCGCTCTTCCGCTTCCTCGCTCACTGACTCGCTGCGCTCGGTCGTTCGGCTGCGGCGAGCGGTATCAGCTC
ACTCAAAGGCGGTAATACGGTTATCCACAGAATCAGGGGATAACGCAGGAAAGAACATGTGAGCAAAAGGCCAGCAA
AAGGCCAGGAACCGTAAAAAGGCCGCGTTGCTGGCGTTTTTCCATAGGCTCCGCCCCCCTGACGAGCATCACAAAAA
TCGACGCTCAAGTCAGAGGTGGCGAAACCCGACAGGACTATAAAGATACCAGGCGTTTCCCCCTGGAAGCTCCCTCG
TGCGCTCTCCTGTTCCGACCCTGCCGCTTACCGGATACCTGTCCGCCTTTCTCCCTTCGGGAAGCGTGGCGCTTTCT
CAATGCTCACGCTGTAGGTATCTCAGTTCGGTGTAGGTCGTTCGCTCCAAGCTGGGCTGTGTGCACGAACCCCCCGT
TCAGCCCGACCGCTGCGCCTTATCCGGTAACTATCGTCTTGAGTCCAACCCGGTAAGACACGACTTATCGCCACTGG
CAGCAGCCACTGGTAACAGGATTAGCAGAGCGAGGTATGTAGGCGGTGCTACAGAGTTCTTGAAGTGGTGGCCTAAC
TACGGCTACACTAGAAGGACAGTATTTGGTATCTGCGCTCTGCTGAAGCCAGTTACCTTCGGAAAAAGAGTTGGTAG
CTCTTGATCCGGCAAACAAACCACCGCTGGTAGCGGTGGTTTTTTTGTTTGCAAGCAGCAGATTACGCGCAGAAAAA
AAGGATCTCAAGAAGATCCTTTGATCTTTTCTACGGGGTCTGACGCTCAGTGGAACGAAAACTCACGTTAAGGGATT
TTGGTCATGAGATTATCAAAAAGGATCTTCACCTAGATCCTTTTAAATTAAAAATGAAGTTTTAAATCAATCTAAAG
TATATATGAGTAAACTTGGTCTGACAGTTACCAATGCTTAATCAGTGAGGCACCTATCTCAGCGATCTGTCTATTTC
GTTCATCCATAGTTGCCTGACTCCCCGTCGTGTAGATAACTACGATACGGGAGGGCTTACCATCTGGCCCCAGTGCT
GCAATGATACCGCGAGACCCACGCTCACCGGCTCCAGATTTATCAGCAATAAACCAGCCAGCCGGAAGGGCCGAGCG
CAGAAGTGGTCCTGCAACTTTATCCGCCTCCATCCAGTCTATTAATTGTTGCCGGGAAGCTAGAGTAAGTAGTTCGC
CAGTTAATAGTTTGCGCAACGTTGTTGCCATTGCTACAGGCATCGTGGTGTCACGCTCGTCGTTTGGTATGGCTTCA
TTCAGCTCCGGTTCCCAACGATCAAGGCGAGTTACATGATCCCCCATGTTGTGCAAAAAAGCGGTTAGCTCCTTCGG
TCCTCCGATCGTTGTCAGAAGTAAGTTGGCCGCAGTGTTATCACTCATGGTTATGGCAGCACTGCATAATTCTCTTA
CTGTCATGCCATCCGTAAGATGCTTTTCTGTGACTGGTGAGTACTCAACCAAGTCATTCTGAGAATAGTGTATGCGG
CGACCGAGTTGCTCTTGCCCGGCGTCAATACGGGATAATACCGCGCCACATAGCAGAACTTTAAAAGTGCTCATCAT
TGGAAAACGTTCTTCGGGGCGAAAACTCTCAAGGATCTTACCGCTGTTGAGATCCAGTTCGATGTAACCCACTCGTG
CACCCAACTGATCTTCAGCATCTTTTACTTTCACCAGCGTTTCTGGGTGAGCAAAAACAGGAAGGCAAAATGCCGCA
AAAAAGGGAATAAGGGCGACACGGAAATGTTGAATACTCATACTCTTCCTTTTTCAATATTATTGAAGCATTTATCA
GGGTTATTGTCTCATGAGCGGATACATATTTGAATGTATTTAGAAAAATAAACAAATAGGGGTTCCGCGCACATTTC
CCCGAAAAGTGCCACCTGACGTCTAAGAAACCATTATTATCATGACATTAACCTATAAAAATAGGCGTATCACGAGG
CCCTTTCGTCTCGCGCGTTTCGGTGATGACGGTGAAAACCTCTGACACATGCAGCTCCCGGAGACGGTCACAGCTTG
TCTGTAAGCGGATGCCGGGAGCAGACAAGCCCGTCAGGGCGCGTCAGCGGGTGTTGGCGGGTGTCGGGGCTGGCTTA
ACTATGCGGCATCAGAGCAGATTGTACTGAGAGTGCACCATATGGACATATTGTCGTTAGAACGCGGCTACAATTAA
TACATAACCTTATGTATCATACACATACGATTTAGGTGACACTATA

M264/pBAR3

Key:

NNNN = Gal promoter

NNNN = Truncated Cre

NNNN = CYC1 Terminator

NNNN = 5' *URA3*

NNNN = *HygMX*

NNNN = Artificial intron start

GAACGCGGCCGCCAGCTGAAGCTTCGTACGCTGCAGGTCGACGGATCCCCGGGTTAATTAAGGCCCACAAGGTTTT
GCATTGAGGATAGTATAGAAGCAAGAATCATTGAATTACAGGAAAAAAAGGCAAATATGATTCATGCTACAATAAAC
CAAGATGAAGCTGCCATTAGCAGACTAACGCCAGCTGATTTACAGTTCTTATTCAATAACTAATATTTTATTCTCTT
ATTATATATTATTCTCGGAGTTTTAAGTGACATCACCCGAAAAGAAGCTAAGTCTTTCTCCTAATTCATATTTAAT
TATTGTACATGGACATATCATACGTAATGCTCAACCTTAGCTAGCtagtacggattagaagccgccgagcgggtgac
agccctccgaaggaagactctcctccgtgcgtcctcgtcttcaccggtcgcgttcctgaaacgcagatgtgcctcgc
gccgcactgctccgaacaataaagattctacaatactagcttttatggttatgaagaggaaaaattggcagtaacct
ggccccacaaaccttcaaatgaacgaatcaaattaacaaccataggatgataatgcgattagttttttagccttatt
tctggggtaattaatcagcgaagcgatgatttttgatctattaacagatatataaatgcaaaaactgcataaccact
ttaactaatactttcaacattttcggtttgtattacttcttattcaaatgtaataaaagtatcaacaaaaaattgtt
aatatacctctatactttaacgtcaaggagaaaaaaccccggattctagaactagtggatccccgggctgcaggaa
ttcgatatcaagcttatcgattgatttacggcgctaaggatgactctggtcagagatacctggcctggtctggacac
agtgcccgtgtcggagccgcgcgagatatggcccgcgctggagtttcaataccggagatcatgcaagctggtggctg
gaccaatgtaaatattgtcatgaactatatccgtaAcctggatagtgaaacaggggcaatggtgcgcctgctggaag
atggcgattagccattaacgcgtaaatgattgctataattatttgatatttatggtgacatatgagaaaggatttca
acatcgacggaaaatatgtagtgctgtctgtaagcactaatattcagtcgccagccgtcattgtcactgtaaagctg
agcgatagaatgcctgatattgactcaatatccgttgcgtttcctgtcaaaagtatgcgtagtgctgaacatttcgt
gatgaatgccaccgaggaagaagcacggcgcggttttgctaaagtgatgtctgagtttggcgaactcttgggtaagg
ttggaattgtcgacctcgagtcatgtaattagttatgtcacgcttacattcacgccctccccccacatccgctctaa
ccgaaaaggaaggagttagacaacctgaagtctaggtccctatttattttttttatagttatgttagtattaagaacg
ttatttatatttcaaatttttcttttttttctgtacagacgcgtgtacgcatgtaacattatactgaaaaccttgct
tgagaaggttttgggacgctcgaaggctttaatttgcggccGGCGCGCCCTTAAGCAGGAGGGTACCGATATCGGAT
CCGTCGACAAAAGCCTCCTTTAGTCCATATTAACAtacatccacatgtgttttagtaaacaaatttgggacctaa
tgcttcaactaactccagtaattccttggtggtacgaacatccaatgaagcacacaagtttgtttgcttttcgtgca
tgatattaaatagcttggcagcaacaggactaggatgagtagcagcacgttccttatatgtagctttcgacatgatt
tatcttcgtttcctgcaggtttttgttctgtgcagttgggttaagaatactgggcaatttcatgtttcttcaacact
acatatgcgtatatataccaatctaagtctgtgctccttccttcgttcttccttCTGTTCGGAGATTACCGAATCGG
TCACCCGGCCAGCGACATGGAGGCCCAGAATACCCTCCTTGACAGTCTTGACGTGCGCAGCTCAGGGGCATGATGTG
ACTGTCGCCCGTACATTTAGCCCATACATCCCCATGTATAATCATTTGCATCCATACATTTTGATGGCCGCACGGCG
CGAAGCAAAAATTACGGCTCCTCGCTGCAGACCTGCGAGCAGGGAAACGCTCCCCTCACAGACGCGTTGAATTGTCC
CCACGCCGCGCCCCTGTAGAGAAATATAAAAGGTTAGGATTTGCCACTGAGGTTCTTCTTTCATATACTTCCTTTTA
AAATCTTGCTAGGATACAGTTCTCACATCACATCCGAACATAAACAACCATGGGTAAAAAGCCTGAACTCACCGCGA
CGTCTGTCGAGAAGTTTCTGATCGAAAAGTTCGACAGCGTCTCCGACCTGATGCAGCTCTCGGAGGGCGAAGAATCT
CGTGCTTTCAGCTTCGATGTAGGAGGGCGTGGATATGTCCTGCGGGTAAATAGCTGCGCCGATGGTTTCTACAAAGA
TCGTTATGTTTATCGGCACTTTGCATCGGCCGCGCTCCCGATTCCGGAAGTGCTTGACATTGGGGAATTCAGCGAGA
GCCTGACCTATTGCATCTCCCGCCGTGCACAGGGTGTCACGTTGCAAGACCTGCCTGAAACCGAACTGCCCGCTGTT
CTGCAGCCGGTCGCGGAGGCCATGGATGCGATCGCTGCGGCCGATCTTAGCCAGACGAGCGGGTTCGGCCCATTCGG
ACCGCAAGGAATCGGTCAATACACTACATGGCGTGATTTCATATGCGCGATTGCTGATCCCCATGTGTATCACTGGC
AAACTGTGATGGACGACACCGTCAGTGCGTCCGTCGCGCAGGCTCTCGATGAGCTGATGCTTTGGGCCGAGGACTGC
CCCGAAGTCCGGCACCTCGTGCACGCGGATTTCGGCTCCAACAATGTCCTGACGGACAATGGCCGCATAACAGCGGT
CATTGACTGGAGCGAGGCGATGTTCGGGGATTCCCAATACGAGGTCGCCAACATCTTCTTCTGGAGGCCGTGGTTGG
CTTGTATGGAGCAGCAGACGCGCTACTTCGAGCGGAGGCATCCGGAGCTTGCAGGATCGCCGCGGCTCCGGGCGTAT
ATGCTCCGCATTGGTCTTGACCAACTCTATCAGAGCTTGGTTGACGGCAATTTCGATGATGCAGCTTGGGCGCAGGG
TCGATGCGACGCAATCGTCCGATCCGGAGCCGGGACTGTCGGGCGTACACAAATCGCCCGCAGAAGCGCGGCCGTCT
GGACCGATGGCTGTGTAGAAGTACTCGCCGATAGTGGAAACCGACGCCCCAGCACTCGTCCGAGGGCAAAGGAATAA
TCAGTACTGACAATAAAAAGATTCTTGTTTTCAAGAACTTGTCATTTGTATAGTTTTTTTATATTGTAGTTGTTCTA
TTTTAATCAAATGTTAGCGTGATTTATATTTTTTTTCGCCTCGACATCATCTGCCCAGATGCGAAGTTAAGTGCGCA
GAAAGTAATATCATGCGTCAATCGTATGTGAATGCTGGTCGCTATACTGCTGTCGATTCGATACTAACGCCGCCATC
CAGTGTCGAAAACGAGCTCCATTAGTGAGTAACTCTGTGATATCTCTCTATAATTAGCAGTTTTTCACTGAAATTCC

AGGAAAGGTAATAAACTCAGATTTTTTTTTATACTATTGGCTGCTTGTTACTTATATATCTTGAACTTCTCCCAGCG
GGTCTTCAAATACATTTGGGCGATGTTCATGTTCATTAGGCAGGTATTTCGACATTGAGTCACACGCGAAAAACCGC
CGGAATTTTTTATGTAATTGCAAGTGGAATTCCGCTGGCAAAACTATTGGGCCCGTTAACCTGCATTAATGAATCGG
CCAACGCGCGGGGAGAGGCGGTTTGCGTATTGGGCGCTCTTCCGCTTCCTCGCTCACTGACTCGCTGCGCTCGGTCG
TTCGGCTGCGGCGAGCGGTATCAGCTCACTCAAAGGCGGTAATACGGTTATCCACAGAATCAGGGGATAACGCAGGA
AAGAACATGTGAGCAAAAGGCCAGCAAAAGGCCAGGAACCGTAAAAAGGCCGCGTTGCTGGCGTTTTTCCATAGGCT
CCGCCCCCCTGACGAGCATCACAAAAATCGACGCTCAAGTCAGAGGTGGCGAAACCCGACAGGACTATAAAGATACC
AGGCGTTTCCCCCTGGAAGCTCCCTCGTGCGCTCTCCTGTTCCGACCCTGCCGCTTACCGGATACCTGTCCGCCTTT
CTCCCTTCGGGAAGCGTGGCGCTTTCTCAATGCTCACGCTGTAGGTATCTCAGTTCGGTGTAGGTCGTTCGCTCCAA
GCTGGGCTGTGTGCACGAACCCCCCGTTCAGCCCGACCGCTGCGCCTTATCCGGTAACTATCGTCTTGAGTCCAACC
CGGTAAGACACGACTTATCGCCACTGGCAGCAGCCACTGGTAACAGGATTAGCAGAGCGAGGTATGTAGGCGGTGCT
ACAGAGTTCTTGAAGTGGTGGCCTAACTACGGCTACACTAGAAGGACAGTATTTGGTATCTGCGCTCTGCTGAAGCC
AGTTACCTTCGGAAAAAGAGTTGGTAGCTCTTGATCCGGCAAACAAACCACCGCTGGTAGCGGTGGTTTTTTTGTTT
GCAAGCAGCAGATTACGCGCAGAAAAAAAGGATCTCAAGAAGATCCTTTGATCTTTTCTACGGGGTCTGACGCTCAG
TGGAACGAAAACTCACGTTAAGGGATTTTGGTCATGAGATTATCAAAAAGGATCTTCACCTAGATCCTTTTAAATTA
AAAATGAAGTTTTAAATCAATCTAAAGTATATATGAGTAAACTTGGTCTGACAGTTACCAATGCTTAATCAGTGAGG
CACCTATCTCAGCGATCTGTCTATTTCGTTCATCCATAGTTGCCTGACTCCCCGTCGTGTAGATAACTACGATACGG
GAGGGCTTACCATCTGGCCCCAGTGCTGCAATGATACCGCGAGACCCACGCTCACCGGCTCCAGATTTATCAGCAAT
AAACCAGCCAGCCGGAAGGGCCGAGCGCAGAAGTGGTCCTGCAACTTTATCCGCCTCCATCCAGTCTATTAATTGTT
GCCGGGAAGCTAGAGTAAGTAGTTCGCCAGTTAATAGTTTGCGCAACGTTGTTGCCATTGCTACAGGCATCGTGGTG
TCACGCTCGTCGTTTGGTATGGCTTCATTCAGCTCCGGTTCCCAACGATCAAGGCGAGTTACATGATCCCCCATGTT
GTGCAAAAAAGCGGTTAGCTCCTTCGGTCCTCCGATCGTTGTCAGAAGTAAGTTGGCCGCAGTGTTATCACTCATGG
TTATGGCAGCACTGCATAATTCTCTTACTGTCATGCCATCCGTAAGATGCTTTTCTGTGACTGGTGAGTACTCAACC
AAGTCATTCTGAGAATAGTGTATGCGGCGACCGAGTTGCTCTTGCCCGGCGTCAATACGGGATAATACCGCGCCACA
TAGCAGAACTTTAAAAGTGCTCATCATTGGAAAACGTTCTTCGGGGCGAAAACTCTCAAGGATCTTACCGCTGTTGA
GATCCAGTTCGATGTAACCCACTCGTGCACCCAACTGATCTTCAGCATCTTTTACTTTCACCAGCGTTTCTGGGTGA
GCAAAAACAGGAAGGCAAAATGCCGCAAAAAAGGGAATAAGGGCGACACGGAAATGTTGAATACTCATACTCTTCCT
TTTTCAATATTATTGAAGCATTTATCAGGGTTATTGTCTCATGAGCGGATACATATTTGAATGTATTTAGAAAAATA
AACAAATAGGGGTTCCGCGCACATTTCCCCGAAAAGTGCCACCTGACGTCTAAGAAACCATTATTATCATGACATTA
ACCTATAAAAATAGGCGTATCACGAGGCCCTTTCGTCTCGCGCGTTTCGGTGATGACGGTGAAAACCTCTGACACAT
GCAGCTCCCGGAGACGGTCACAGCTTGTCTGTAAGCGGATGCCGGGAGCAGACAAGCCCGTCAGGGCGCGTCAGCGG
GTGTTGGCGGGTGTCGGGGCTGGCTTAACTATGCGGCATCAGAGCAGATTGTACTGAGAGTGCACCATATGGACATA
TTGTCGTTAGAACGCGGCTACAATTAATACATAACCTTATGTATCATACACATACGATTTAGGTGACACTATA

Sequences:

PCR product of P18+P27 in *NatMX/*3' iSeq barcode construct haploids

Key:
NNNN = iSeq 3' Barcode
NNNN = P18 binding site
NNNN = lox66 sequence

```
cccgcagagtactgcaatttgactgtattaccaatgtcagcaaattttctgtcttcgaagagtaaaaaattgtactt
ggcggataatgcctttagcggcttaactgtgccctccatggaaaaatcagtcaagatctgTTATAAATAATACCATT
TGTTAGTAAAAATTCGAATTCAAGCTTAGATCTGATATCGGTACCNNNNNAANNNNNTTNNNNNTTNNNNNATAACT
TCGTATAGCATACATTATACGAACGGTAGGCGC
```

PCR product of P19+P28 in *KanMX/*5' iSeq barcode construct haploids

Key:
NNNN = iSeq 5' Barcode
NNNN = P19 binding site
NNNN = lox71 sequence

```
tgcacgaaaagcaaacaaacttgtgtgcttcattggatgttcgtaccaccaaggaattactggagttagttgaagca
ttaggtcccaaaatttgtttactaaaaacacatgtggatgtaTGTTAATATGGACTAAAGGAGGCTTTTGTCGACGG
ATCCGATATCGGTACCNNNNNAANNNNNAANNNNNTTNNNNNATAACTTCGTATAATGTATGCTATACGAACGGTAG
GCG
```

PCR product of P18+P19 Double Deletion/iSeq Double Barcode haploids

NNNN = iSeq Barcodes
NNNN = P19 binding site
NNNN = loxP sequence

```
tgcacgaaaagcaaacaaacttgtgtgcttcattggatgttcgtaccaccaaggaattactggagttagttgaagca
ttaggtcccaaaatttgtttactaaaaacacatgtggatgtaTGTTAATATGGACTAAAGGAGGCTTTTGTCGACGG
ATCCGATATCGGTACCNNNNNAANNNNNAANNNNNTTNNNNNATAACTTCGTATAATGTATGCTATACGAAGTTATN
NNNNAANNNNNAANNNNNTTNNNNNGGTACCGATATCAGATCTAAGCTTGAATTCGAATTTTTACTAACAAATGGTA
TTATTTATAAcagatcttgactgatttttccatggagggcacagttaagccgctaaaggcattatccgccaagtaca
atttttactcttcgaagacagaaaatttgctgacattggtaatacagtcaaattgcagtactctgcggg
```

References

Gietz, R. D. and Schiestl, R. H. (2007). High-efficiency yeast transformation using the LiAc/SS carrier DNA/PEG method. Nature protocols. *2*, 31-34.

Herman, P. K. and Rine, J. (1997). Yeast spore germination: a requirement for Ras protein activity during re-entry into the cell cycle. The EMBO Journal. *16*, 6171-6181.

Smith, A. M., Heisler, L. E., Mellor, J., Kaper, F., Thompson, M. J., Chee, M., et al. (2009). Quantitative phenotyping via deep barcode sequencing. Genome research. *19*, 1836-1842.

Winzeler, E. A., Shoemaker, D. D., Astromoff, A., Liang, H., Anderson, K., Andre, B., et al. (1999). Functional characterization of the S. cerevisiae genome by gene deletion and parallel analysis. Science. *285*, 901-906.
