# Supplementary material for: iSeq: A New Double-Barcode Method for Detecting Dynamic Genetic Interactions in Yeast
Source: G3 (Bethesda). 2016 Nov 7;7(1):143–53. doi: 10.1534/g3.116.034207 (PMC5217104; doi:10.1534/g3.116.034207)
Supplement: Supplementary file 6 [file 143FileS2.pdf]

Supplemental Figure Legends:

Figure S1. A) Relative frequency trajectories for all conditions and replicates. Each line is an individual double barcode strain. Colors indicate the fitness estimate of each strain. **B,C)** Scatter plots of fitnesses **(B)** and interaction scores **(C)** between each of three biological replicates in each of three experimental conditions. Each point is an individual strain.

Figure S2. Scatter plot of the mean and the standard deviation of fitness estimates made in three replicate cultures in YPD (**A**), YPD 37°C (**B**) and YPEG (**C**). Each point is an individual strain. Spearman's *rho* and *P*-value are given on the plots.

Figure S3. Scatter plot of iSeq interaction score estimates and s-scores reported by Collins *et. al.* 2007 (**A**) and interaction scores reported in Costanzo *et. al.* 2010 (**B**). Each point is a gene pair. Error bars on our measurements represent the standard deviation across 4-8 strains carrying the corresponding gene deletions. In panel **B**, two scores were published for a subset of gene pairs. Error bars on Costanzo *et. al.* data are the standard deviations reported on the estimates.

Figure S4. Spot assays for F0 strains from the 5 double gene deletion genotypes that were whole-genome sequenced. Overnight cultures were inoculated from single colonies and diluted to $1x10^8$ cells/mL. Four additional serial dilutions (1:10) were performed, and 3ul of each dilution was plated to either YPD or the media used to select haploid double deletion strains carrying the iSeq double barcode (1.7g/L Difco yeast nitrogen base without amino acids and ammonium sulfate, 1g/L monosodium glutamic acid, 2% glucose, Nat, G418, –Ura, –Lys, –Leu, –Ade, and –Arg). The number of cells plated (columns), and the whole chromosome duplication events identified in each strain (rows), are labeled on each panel.

Supplemental Table Titles:

Table S1. Published genetic interactions for each of the 36 double gene deletion genotypes used in this study. Quantitative scores are either an SGA score (ε), or an S score (*s*). See references for sources of reported interactions. All published interactions were found in the BioGRID repository.
Table S2. (Separate excel file) Fitness and interaction score estimates for each individual strain in each replicate of each experimental condition.
Table S3. (Separate excel file) Expanded summary of whole genome sequencing data.
Table S4. (Separate excel file) Interaction score estimates called as significant using 95% confidence intervals.
Table S5. (Separate excel file) Sequencing counts for each strain, at each time point, in each culture, and each strain's doubling time and fitness estimates from Optical Density growth curve fitness assay.
