# Supplementary material for: iSeq: A New Double-Barcode Method for Detecting Dynamic Genetic Interactions in Yeast
Source: G3 (Bethesda). 2016 Nov 7;7(1):143–53. doi: 10.1534/g3.116.034207 (PMC5217104; doi:10.1534/g3.116.034207)
Supplement: Supplementary file 7 [file 143TableS1.pdf]

Table S1

Gene Pair	Type	Publi-cations	Published Quantitative GI Scores	Published Non-Quantitative GIs
arp6Δsin3Δ	Negative	4	ε = -0.4711[1]<br>s = -9.0381[2]	Synthetic Lethal[3]<br>Synthetic Growth Defect[4]
sin3Δsnt1Δ	Negative	3	ε = -0.3606[1]<br>s = -7.4923[2]<br>s = -3.8016[5]	
arp6Δdep1Δ	Negative	3	ε = -0.3964[1]<br>s = -9.2601[2]	Synthetic Lethality[6]
arp6Δsap30Δ	Negative	5	ε = -0.4597[1]<br>s = -12.3508[2]	Synthetic Lethality[6]<br>Synthetic Growth Defect[3,4]
arp6Δsds3Δ	Negative	3	ε = -0.367[1]<br>s = -10.7073[2]	Synthetic Growth Defect[4]
arp6Δrpd3Δ	Negative	4	ε = -0.412[1]<br>s = -6.631[2]	Synthetic Lethal[3]<br>Synthetic Growth Defect[4]
arp6Δpho23Δ	Negative	5	ε = -0.4046[1]<br>s = -11.7012[2]	Synthetic Growth Defect[3,4,6]
dep1Δsnt1Δ	Negative	3	ε = -0.3668[1]<br>s = -12.3968[2]	Synthetic Lethality[6]
arp6Δsnt1Δ	Negative	2	ε = -0.3884[1]	Synthetic Growth Defect[4]
snt1Δsds3Δ	Negative	3	ε = -0.2734[1]<br>s = -8.2462[2]<br>s = -8.389[5]	
snt1Δdgk1Δ	Negative	1	ε = -0.3551[1]	
snt1Δpho23Δ	Negative	3	ε = -0.3436[1]<br>s = -3.947[2]<br>s = -3.5671[5]	
snt1Δrpd3Δ	Negative	2	ε = -0.3372[1]	Phenotypic Enhancement[7]
sap30Δsnt1Δ	Negative	2	ε = -0.1983[1]<br>s = -6.7316[2]	
sin3Δsds3Δ	Positive	1	ε = 0.1777[1]	

Table S1

Gene Pair	Type	Publi-cations	Published Quantitative GI Scores	Published Non-Quantitative GIs
*dgk1*Δ*sin3*Δ	Positive	1		Phenotypic Suppression[7]
*sin3*Δ*pho23*Δ	Positive	3	s = 2.9662[5]<br>s = 2.0226[8]	Positive Genetic[9]
*sin3*Δ*rpd3*Δ	Positive	2	s = 4.6737[2]	Positive Genetic[9]
*sin3*Δ*sap30*Δ	Positive	3	ε = 0.1894[1]<br>s = 2.1593[10]	Positive Genetic[9]
*dep1*Δ*rpd3*Δ	Positive	1	s = 3.0745[2]	
*rpd3*Δ*pho23*Δ	Positive	1		Positive Genetic[9]
*dgk1*Δ*sds3*Δ	Positive	1	ε = 0.2087[1]	
*sap30*Δ*sds3*Δ	Positive	2	ε = 0.1995[1]<br>s = 2.7149[5]	
*dep1*Δ*pho23*Δ	Positive	1	ε = 0.1659[1]	
*sds3*Δ*pho23*Δ	Positive	2	ε = 0.3153[1]<br>s = 2.8751[5]	
*dep1*Δ*sds3*Δ	Positive	1	ε = 0.4403[1]	
*sds3*Δ*rpd3*Δ	Positive	2	ε = 0.3473[1]	Phenotypic Suppression[11]
*sap30*Δ*pho23*Δ	Positive	2	s = 2.532[5]	Positive Genetic[9]
*rpd3*Δ*sap30*Δ	Positive	1		Positive Genetic[9]
*sin3*Δ*dep1*Δ	None			
*dep1*Δ*sap30*Δ	None			
*dgk1*Δ*dep1*Δ	None			
*dgk1*Δ*arp6*Δ	None			
*dgk1*Δ*pho23*Δ	None			
*dgk1*Δ*sap30*Δ	None			
*dgk1*Δ*rpd3*Δ	None			

Source Publications:

1. Costanzo, M. *et al.* The genetic landscape of a cell. *Science* **327,** 425–431 (2010).

Table S1

2. Collins, S. R. *et al.* Functional dissection of protein complexes involved in yeast chromosome biology using a genetic interaction map. *Nature* **446,** 806–810 (2007).
3. Lin, Y.-Y. *et al.* A comprehensive synthetic genetic interaction network governing yeast histone acetylation and deacetylation. *Genes Dev.* **22,** 2062–2074 (2008).
4. Krogan, N. J. *et al.* A Snf2 family ATPase complex required for recruitment of the histone H2A variant Htz1. *Mol. Cell* **12,** 1565–1576 (2003).
5. Zheng, J. *et al.* Epistatic relationships reveal the functional organization of yeast transcription factors. *Mol. Syst. Biol.* **6,** 420 (2010).
6. Tong, A. H. Y. *et al.* Global mapping of the yeast genetic interaction network. *Science* **303,** 808–813 (2004).
7. Beltrao, P. *et al.* Evolution of phosphoregulation: comparison of phosphorylation patterns across yeast species. *PLoS Biol.* **7,** e1000134 (2009).
8. Bandyopadhyay, S. *et al.* Rewiring of genetic networks in response to DNA damage. *Science* **330,** 1385–1389 (2010).
9. Breslow, D. K. *et al.* A comprehensive strategy enabling high-resolution functional analysis of the yeast genome. *Nat. Methods* **5,** 711–718 (2008).
10. Wilmes, G. M. *et al.* A genetic interaction map of RNA-processing factors reveals links between Sem1/Dss1-containing complexes and mRNA export and splicing. *Mol. Cell* **32,** 735–746 (2008).
11. Torres-Machorro, A. L. & Pillus, L. Bypassing the requirement for an essential MYST acetyltransferase. *Genetics* **197,** 851–863 (2014).
