# Supplementary material for: iSeq: A New Double-Barcode Method for Detecting Dynamic Genetic Interactions in Yeast
Source: G3 (Bethesda). 2016 Nov 7;7(1):143–53. doi: 10.1534/g3.116.034207 (PMC5217104; doi:10.1534/g3.116.034207)
Supplement: Supplementary file 10 [file 143TableS3.docx]

**Table S3.** Expanded summary of whole genome sequencing data. (.xlsx, 89 KB)

http://www.g3journal.org/lookup/suppl/doi:10.1534/g3.116.034207/-/DC1/TableS3.xlsx
