# Supplementary material for: iSeq: A New Double-Barcode Method for Detecting Dynamic Genetic Interactions in Yeast
Source: G3 (Bethesda). 2016 Nov 7;7(1):143–53. doi: 10.1534/g3.116.034207 (PMC5217104; doi:10.1534/g3.116.034207)
Supplement: Supplementary file 12 [file 143TableS4.docx]

**Table S4.** Interaction score estimates called as significant using 95% confidence intervals. (.xlsx, 58 KB)

http://www.g3journal.org/lookup/suppl/doi:10.1534/g3.116.034207/-/DC1/TableS4.xlsx
