# Supplementary material for: iSeq: A New Double-Barcode Method for Detecting Dynamic Genetic Interactions in Yeast
Source: G3 (Bethesda). 2016 Nov 7;7(1):143–53. doi: 10.1534/g3.116.034207 (PMC5217104; doi:10.1534/g3.116.034207)
Supplement: Supplementary file 14 [file 143TableS5.docx]

**Table S5.** Sequencing counts for each strain, at each time point, in each culture, and each

strain’s doubling time and fitness estimates from Optical Density growth curve fitness assay. (.xlsx, 245 KB)

http://www.g3journal.org/lookup/suppl/doi:10.1534/g3.116.034207/-/DC1/TableS5.xlsx
